# METTL5 regulates SEPHS2-mediated selenoprotein synthesis to promote multiple myeloma survival and progression

**DOI:** 10.1038/s41419-025-07904-6

**Published:** 2025-08-02

**Authors:** Junyao Jiang, Fangmin Zhong, Zuomiao Xiao, Fangyi Yao, Jing Liu, Meiyong Li, Huan Zeng, Yuxiang Qiu, Jing Zhang, Haibin Zhang, Shuqi Li, Ting huang, Wenli Feng, Zhenglan Huang, Bo Huang, Xiaozhong Wang

**Affiliations:** 1 Jiangxi Province Key Laboratory of Immunology and Inflammation, Jiangxi Provincial Clinical Research Center for Laboratory Medicine, Department of Clinical Laboratory, The Second Affiliated Hospital, Jiangxi Medical College, Nanchang University, Nanchang, Jiangxi P. R. China; 2 Department of Clinical Laboratory, The Affiliated Ganzhou Hospital of Nanchang University, Ganzhou, Jiangxi P. R. China; 3 Department of Blood Transfusion, The Second Affiliated Hospital, Jiangxi Medical College, Nanchang University, Nanchang, Jiangxi P. R. China; 4 Department of Clinical Hematology, Key Laboratory of Laboratory Medical Diagnostics Designated By Ministry of Education, School of Laboratory Medicine, Chongqing Medical University, Chongqing, P. R. China

**Keywords:** Cancer epidemiology, Apoptosis

## Abstract

The abnormal expression of m^6^A methyltransferase is a significant factor in the occurrence and progression of tumors. The 18S ribosomal RNA (rRNA) m^6^A methyltransferase Methyltransferase-like 5 (METTL5) is upregulated in various cancers, leading to adverse prognosis by abnormally regulating protein translation in tumor cells. However, the functionality and molecular mechanisms of METTL5 in the progression of multiple myeloma (MM) remain unknown. In this study, we demonstrate that the expression of METTL5 in the bone marrow (BM) of newly diagnosed MM patients is significantly higher than in healthy individuals and patients in remission following treatment. Importantly, we found that MM patients with upregulated METTL5 expression had a poorer prognosis. Additionally, we show that METTL5 plays a key role in promoting MM progression both in vitro and in an orthotopical xenograft model. Mechanistically, the depletion of METTL5 expression mediates a decrease in overall translation efficiency and selenium metabolism-related signaling pathway levels. We further revealed that the reduction in selenophosphate synthetase 2 (SEPHS2) translation efficiency mediated by METTL5 depletion can lead to diminished synthesis of selenoproteins and increased reactive oxygen species (ROS), thereby inducing apoptosis in MM. Salvianolic acid C (SAC) was identified as a potential METTL5 inhibitor, demonstrating significant pro-apoptotic effects during the treatment of MM both in vitro and in vivo. In summary, our research highlights the critical role of METTL5 in the progression of MM cells. Our data indicate METTL5’s function is to influence the overall translation efficiency and reprogram selenium metabolism to inhibit apoptosis. Therefore, SAC may be an ideal candidate drug for suppressing MM progression.

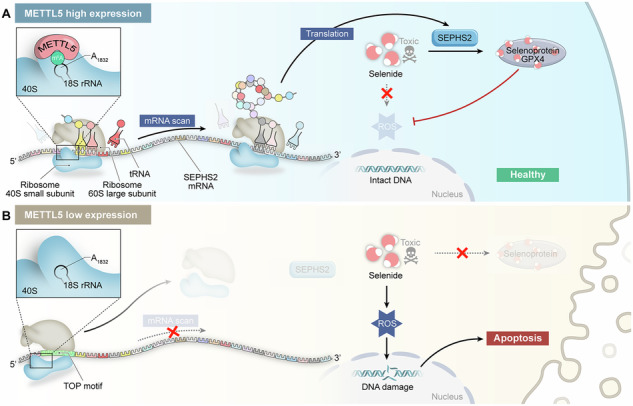

## Introduction

Multiple myeloma (MM) is the second most common hematological cancer originating from bone marrow B cells [1]. The immunoglobulin secreted by clonal plasma cells can inflict damage to multiple organs. Predominantly, MM patients exhibit symptoms such as anemia, hypercalcemia, bone destruction, and renal dysfunction [2]. Presently, treatments incorporating monoclonal antibodies, immunomodulatory drugs, proteasome inhibitors, and stem cell transplantation serve to improve the prognosis for MM patients [3]. However, relapse following treatment and onset of refractory disease remain significant challenges in managing MM [4, 5]. As such, advancing the development of new treatment strategies is critical to prolonging patient survival.

N6-methyladenosine (m^6^A) is the most prevalent internal chemical modification in eukaryotic RNA. Accumulating evidence reveals that m6A modification plays a pivotal role in controlling RNA processing, splicing, nucleation, translation, and stability; factors that are integral to the development of many diseases, including cancer [6]. Methyltransferase-like 5 (METTL5) is a methyltransferase located in the nucleolus, where ribosome biosynthesis occurs, that is responsible for m^6^A modification [7]. Its primary function lies in modifying the A_1832_ site of 18S rRNA with m^6^A, thus affecting the maturity of 18S rRNA and subsequently influencing the global translation of mRNA by ribosomes [8]. At present, research related to METTL5 is relatively scarce. Existing studies demonstrate that high expression of METTL5 plays an instrumental role in lipid metabolism and the pathogenesis of liver cancer [9]. Further, high METTL5 expression is closely linked to malignant proliferation in breast cancer and a poor prognosis in gastric cancer [10]. Bioinformatics research suggests an association between high METTL5 expression and chemotherapy resistance in non-small cell lung cancer, as well as resistance to PD-1 immunotherapy [11]. Consequently, abnormal expression of METTL5 may constitute a significant pathogenic mechanism in numerous cancer types and thus deserves further investigation.

Selenium, an essential trace element for human health, is implicated in various pathophysiological conditions [12]. A multitude of studies highlight selenium’s crucial role in preventing oxidative DNA damage and promoting DNA repair, hinting at its potential significance in cancer development [13, 14]. Selenophosphate synthetase 2 (SEPHS2), a selenophosphate synthetase, catalyzes the conversion of selenite and adenosine triphosphate (ATP) into selenophosphate. Both inorganic and organic selenium are predominantly reduced to selenite in the intestine following ingestion [13]. Previous studies have proposed that selenite exhibits toxicity towards tumor cells and requires conversion into selenophosphate by SEPHS2, which is further transformed into the rare amino acid selenocysteine to foster cell growth [15]. This transformation is vital for selenium-dependent proteins such as glutathione peroxidase and thioredoxin reductase [16]. Hence, SEPHS2 likely plays a pivotal role in maintaining the homeostasis of tumor cells, and could potentially serve as a target for cancer treatment [17].

In this investigation, we found that METTL5 levels in MM patients were elevated and correlated with a negative prognosis. Diminished METTL5 suppressed the tumorigenesis and progression of MM (Supplementary Table 1). Mechanistically, METTL5-mediated modification of 18S rRNA m6 at A^1832^ controlled the global translation of mRNA by modulating its recognition by the 40S ribosomal subunit. Following impairment of METTL5, the translation efficiency of SEPHS2 declines, leading to a hindrance in selenium metabolism that triggers oxidative stress and DNA damage, ultimately leading to cell apoptosis. Our findings highlight how METTL5 regulates gene expression and disease progression during MM, and provide a molecular foundation for the development of therapeutic strategies aimed at targeting this pathway to achieve better therapeutic responses for cancer patients.

## Material and methods

Please refer to supplementary Table 1 for information on the sources of all reagents used in this study.

### Clinical samples

A total of 22 fresh primary diagnosis and 11 complete treatment response bone marrow (BM) samples from patients with MM (20 male and 13 female, aged between 46 and 71 years old), and 15 healthy normal donor BM smears (8 male and 7 female, aged between 50 and 70 years old) treated at our hospital between January 2021 and October 2022 were obtained. MM cells were isolated by positive selection from fresh BM samples using CD138 MicroBeads (Miltenyi Biotec, Germany) and subjected to RNA and protein extraction. Purity of isolated CD38+ and CD138+ cells was determined by flow cytometry prior to downstream analyses. Informed consent was obtained from each individual. Ethical approval was obtained from the Research Ethics Committee of the Second Affiliated Hospital of Nanchang University (approval No. 2022-356).

### Plasmid and lentiviral transduction

For shRNA plasmids used in lentivirus-mediated interference, complementary sense and antisense oligonucleotides encoding shRNAs targeting METTL5 or SEPHS2 were synthesized, annealed, and cloned into the pRRLSIN-cPPT-U6-shRNA-SFFV-EGFP-SV40-puromycin vector (GV644, GeneChem, China) and hU6-MCS-CBh-gcGFP-IRES-puromycin vector (GV493, GeneChem, China). The related sequences of shRNAs are shown in supplementary Table 4. METTL5 and SEPHS2 expression plasmids were constructed by cloning the METTL5 and SEPHS2 genes into the Ubi-MCS-3FLAG-CBh-gcGFP-IRES-puromycin vector (GV492, GeneChem, China) and the Ubi-MCS-SV40-EGFP-IRES-Hygromycin (GV550, GeneChem, China) vector, respectively. Sequences of METTL5 and SEPHS2 target sequences and shRNAs are listed in Supplementary Table 4. METTL5 or SEPHS2 lentiviruses and their negative controls under the action of Hitrans B2 (GeneChem, China) were used to generate MM cell lines with stable knockdown or overexpression of METTL5 or SEPHS2. Subsequently, puromycin was used to select cells that had been successfully infected with the lentiviruses. Finally, the expression levels of METTL5 or SEPHS2 proteins were detected using RT-qPCR and Western blot analyses.

### RNA formaldehyde denaturing gel electrophoresis

To create a 1% agarose gel with a formaldehyde concentration of 2.2 M, RNA samples were combined with 10× denaturing buffer and incubated at 65 °C for 10 min. Subsequently, 10× RNA Glycerol Gel Loading Buffer and ethidium bromide (EtBr) (Solarbio, China) buffer were added into each of the sample wells. Electrophoresis was carried out in 1× 3-(N-Morpholino) propane-sulfonic acid (MOPS) buffer at 150 V for 30 min, followed by visualization of electrophoretic bands on a gel imager.

### 18S ribosomal RNA isolation

The 18S rRNA fragment of the indicated samples was isolated from an agarose gel following formaldehyde denaturation electrophoresis. Fragments were transferred to a nuclease-free Eppendorf tube, where RAD Buffer was added to dissolve the gel. Samples were incubated at 50 °C for 5 min. Subsequently, the dissolved samples were added to a Zymo-Spin™ IC Column for centrifugation, washed three times with RNA Prep Buffer, and finally resuspended in Diethyl dicarbonate (DEPC)-treated water.

### Quantitative analysis of m^6^A by LC–MS/MS

S1 nuclease (Takara, China) and Alkaline Phosphatase (Takara, China) buffers were added to 200 ng total RNA and 18S rRNA, respectively. Samples were incubated at 37 °C for 6 h prior to the performance of liquid chromatography tandem mass spectrometry (LC–MS/MS) detection of m^6^A. The sample extracts were analyzed using an ultra-performance liquid chromatography (UPLC)–MS/MS system (UPLC, ExionLC™, Qtrap 4500; ABSciex, USA). Samples were run on an Acquity UPLC HSS T3 column (1.8 μm × 2.1 mm, i.d., Waters, Made in Ireland) using a water: methanol solvent system at a flow rate of 0.25 mL/min at 40 °C. Samples were run using an injection volume of 4 μL using a 96:4 V/V at 0 and 2.5 min, 31:69 V/V at 2.7 and 6 min, 95:5 V/V at 6.2 and 6.7 min, 4:96 V/V at 6.9 and 7.4 min gradient program. Nucleosides were detected in positive electrospray ionization mode. The quantification of nucleosides was based on the nucleoside-to-base ion mass transitions of 282.1 to 150.1 for m6A, 268.1 to 136.1 for A, and 243.1 to 127.0 for thymidine (internal standard) using standard curves generated in the same batch of samples with pure nucleosides. This approach involves calculating the ratio *f* (*f* = As/Ai), where As represents the area of each component and Ai represents the peak area of the internal target. A standard curve equation was obtained through regression calculation, using the peak area ratio/weight over the concentration “C” of each nucleoside. The modification level of m^6^A in the sample (m^6^A/A) is given by the equation *f*_m6A_/*f*_A_. All experiments were repeated at least three times under identical conditions.

### Cell cycle, ROS, and apoptosis detection and analysis

For cell cycle, ROS, and apoptosis analyses, propidium iodide Solution (Biolegend, USA), CellROX® Deep Red Reagent (Thermo Fisher, USA) and the APC Annexin V Apoptosis Detection with PI Kit (Biolegend, USA) were used following the manufacturer’s instructions. After cell staining, cells were analyzed using a NovoCyte flow cytometer (D3080-3080, Agilent, USA). The data were analyzed using NovoExpress software Version 1.6.2 (Agilent, USA). All experiments were repeated at least three times under identical conditions.

### Orthotopical xenograft models

Animal experiments were carried out in accordance with the Institute of Biophysics, Chinese Academy of Science’s Policy on Care and Use of Laboratory Animals. All mouse studies were approved by the Animal Ethical and Welfare Committee of Nanchang University (NCULAE-20221031183). Female NOD.Cg-Prkdc^scid^Il2rg^em1Sm^°^c^ (NSG, RRID: IMSR_NM-NSG -003) mice aged 6–8 weeks old were purchased from Jiangsu GemPharmtech, Inc. (China). To ensure the reliability of the results, we conducted in vivo growth experiments (*n* = 4 in each group) and drug treatment experiments (*n* = 6 in each group) using at least three NSG mice per group. This approach was chosen to guarantee sufficient statistical power and reproducibility of the findings. Cells carrying a luciferase reporter were harvested and injected into NSG mice via tail vein injection (2 × 10^6^ cells transfected with lentivirus in 200 μL PBS; 5 × 10^6^ RPMI-8226-luc or RPMI-8226-METTL5^OE^-luc cells in 200 μL PBS). Two weeks post-cell injection, whole-body bioluminescence imaging (BLI) was used to affirm tumor formation of MM cells within mice. In the drug treatment experiments, the NSG mice were randomly assigned to different groups based on their in vivo BLI values, ensuring that the average BLI values of each group were approximately equal. This approach helps to minimize baseline differences and ensures the comparability of the groups, thereby enhancing the validity and reliability of the experimental results. Following tumorigenesis, mouse weight was logged every three days, and a weight fluctuation curve was charted. In vivo imaging was conducted weekly to track the infiltration of MM cells within mice. Upon reaching a moribund state (reduced activity and lethargy, fluffy and untidy fur coats, unresponsiveness to external stimuli, and an inability to quickly get up when laid on its side, etc.), mice were euthanized and survival time noted. Femur and tibia bones were harvested, and bone marrow was collected to prepare bone marrow smears for subsequent IHC detection. For drug-treated mouse models, liver, kidney, intestine, and lung tissues were collected for downstream H&E staining studies.

### Bioluminescence imaging (BLI)

For BLI measurements, mice received an intraperitoneal injection with sterile d-Luciferin (15 ml/kg). Then, 5 min later, mice were anesthetized and placed in positioned within the bioluminescence imaging system for comprehensive whole-body BLI analysis.

### High-throughput virtual screen for potential METTL5 inhibitors

For the target protein, the crystal structure obtained from the PDB database (https://www.rcsb.org/) was preprocessed by removing water molecules and miscellaneous atoms from the protein structure, protonation to add hydrogen atoms, and the use of the existing ligand SAM in the protein to define the binding site. Next, we added the prepared ligand small molecule, add CHARMm force field, and use Smart Minimizer algorithm to minimize its energy, the maximum number of steps is set to 2000, and the RMS gradient value is set to 0.01. A total of 12,654 minimized poses were generated. The Discovery Studio Libdock program was used to screen for molecular docking, with scoring based on Libdock score and the top 10 compounds with Libdock score (table) were selected for further analysis. Next, the ligand and protein needed for molecular docking were prepared using AutoDock Vina software (http://vina.scripps.edu/) for the top 10 compounds and the Vina tool in PyRx software (https://pyrx.sourceforge.io/) for docking. The affinity (kcal/mol) value represents the binding ability of the two.

### Statistical analysis

For comparisons between the two groups, continuous variables were analyzed using Student’s *t*-test, and categorical variables were analyzed using the chi-square test. A two-way analysis of variance (ANOVA) was used to compare multiple groups. Kaplan–Meier survival curves were plotted, and log-rank tests were used to analyze the data. GraphPad Prism 8 was used to derive *P* values, which are indicated as *P < 0.05, **P < 0.01, ***P < 0.001 and ****P < 0.0001. All analyses were based on the data obtained from at least three independent experiments, and the data are presented as the means ± standard deviations (SDs).

## Results

### METTL5 is upregulated and is a prognostic value in MM

**Fig. 1:**
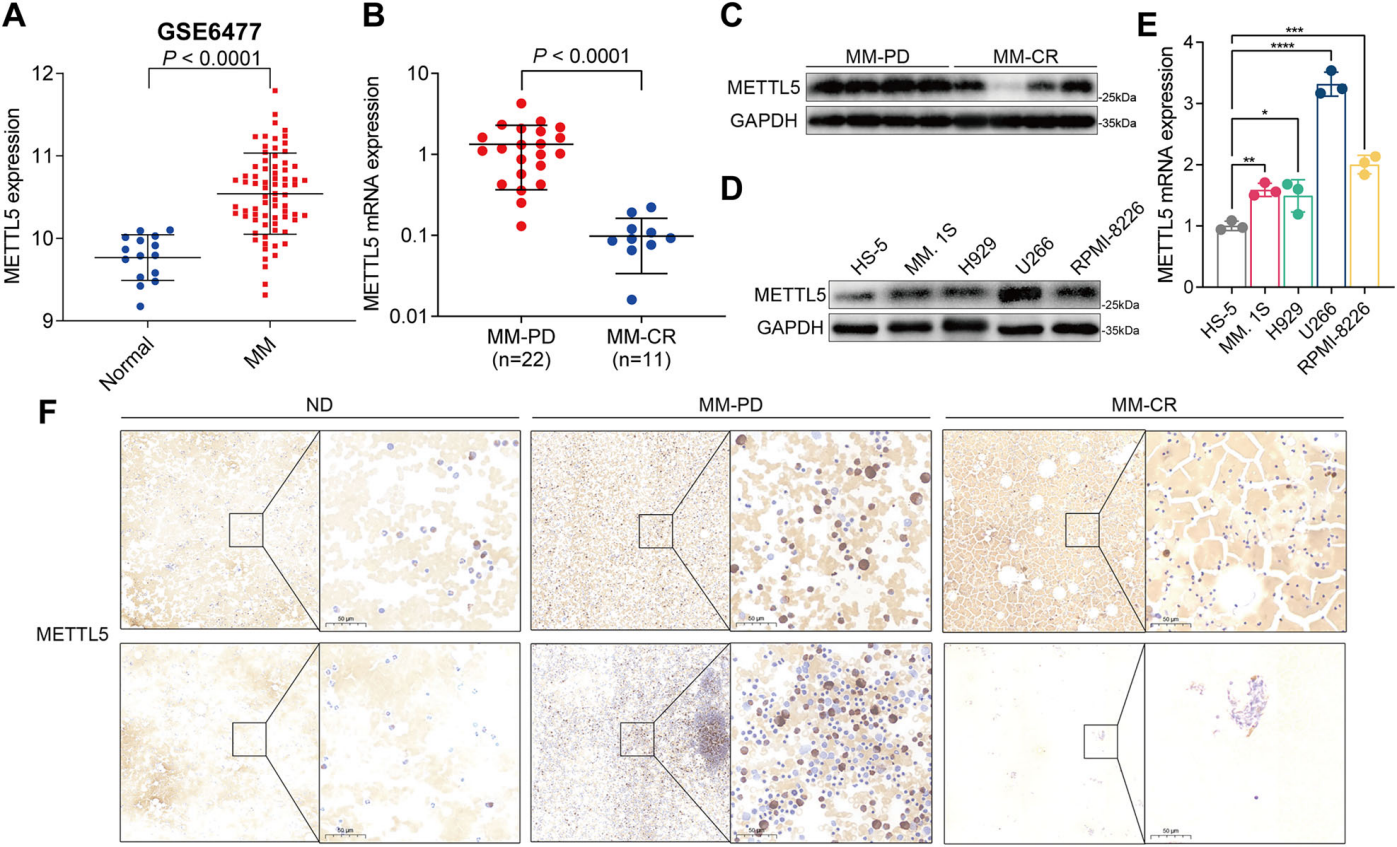
METTL5 overexpression is associated with a poor prognosis in MM patients. **A** METTL5 expression in the GSE6477 MM cohort (*n* = 15 in normal donor (ND) and *n* = 73 in MM groups). **B** METTL5 mRNA expression between MM-CR and MM-PD BM samples in our cohort (*n* = 22 in MM-PD and *n* = 11 in MM-CR). **C** METTL5 protein expression between MM-CR and MM-PD BM samples in our cohort (*n* = 4 in each group). **D**, **E** Protein (**D**) and mRNA (**E**) expression levels of METTL5 in BM stromal cells (HS-5) and MM cells. **F** Representative IHC staining images of METTL5 from ND (left), MM-PD (mid) and MM-CR (right) BM samples. Scale bars: left panels, 200 μm; right panels, 50 μm. Independent biological experiments were repeated at least three times, and the data are presented as the means ± SDs. Statistical differences are indicated by p-values of *P < 0.05, **P < 0.01, ***P < 0.001 and ****P < 0.0001.

To explore the potential function of m^6^A “writers” in MM, we analyzed the GSE6477 dataset of MM patients and normal donors (NDs) and found that METTL5 mRNA expression was significantly upregulated in MM patients compared to NDs (Fig. 1A, supplementary Fig. 1A–D). To further understand the importance of METTL5 during the progression of MM, we also assessed METTL5 expression in patients with monoclonal gammopathy of undetermined significance (MGUS) and smoldering MM (SMM), which are considered precancerous conditions of MM [1]. Strikingly, the abundance of METTL5 mRNA gradually increased as plasma cell lesions advanced in samples within the GSE6477 dataset (Supplementary Fig. 1E). Furthermore, high METTL5 expression in bone marrow (BM) correlated with poor overall survival (OS) (Supplementary Fig. 1F). Subsequently, we examined METTL5 expression in our MM cohort. As expected, both mRNA and protein expression levels of METTL5 were significantly elevated in MM primary diagnosis (MM-PD) patient BM samples compared to MM complete response (MM-CR) patient BM samples (Fig. 1B, C, Supplementary Fig. 1G). In comparison to normal bone marrow stromal cell HS-5, all MM cell lines demonstrated significantly elevated levels of METTL5 mRNA and protein (Fig. 1D, E). We selected the cell lines based on the mRNA and protein expression levels of METTL5 in MM cell lines. Specifically, we chose the “RPMI-8226” cell line, which exhibits moderate METTL5 expression, for both overexpression and knockdown studies. The H929 cell line, which has the lowest METTL5 expression, was selected for overexpression, while the U266 cell line, which has the highest METTL5 expression, was chosen for knockdown. Immunohistochemical (IHC) analysis of BM smears revealed that patients in the MM-PD group exhibited significantly higher METTL5 expression in the bone marrow compared to patients in the MM-CR group and the ND group (Fig. 1F). These data suggest that METTL5 expression in MM patients is markedly higher than in NDs, and its progressive increase in expression is closely associated with the progression of plasma cell diseases and a poor prognosis in MM patients.

### Upregulation of METTL5 accelerates the progression of MM

**Fig. 2:**
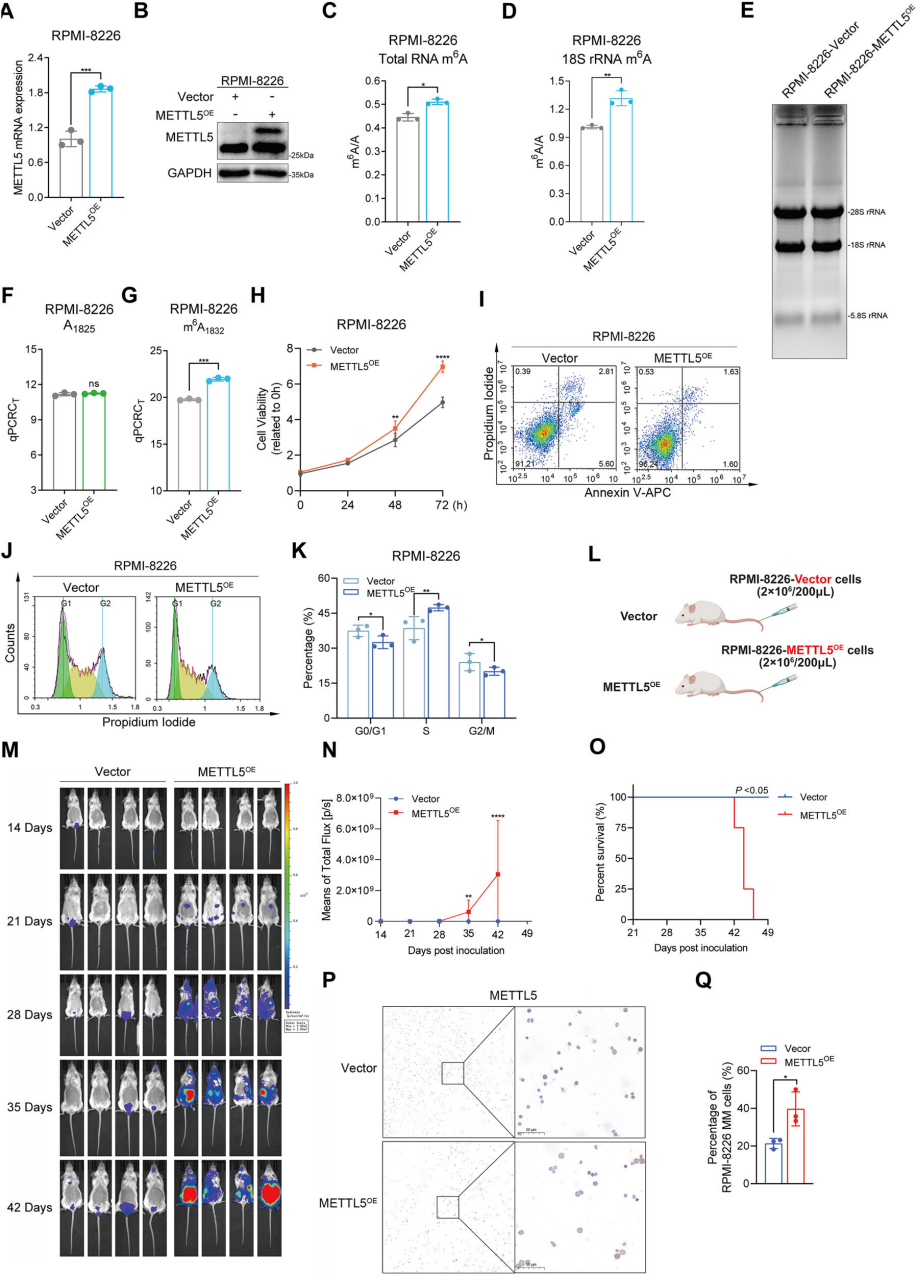
Overexpression of METTL5 accelerates the progression of MM. **A** The mRNA expression of *METTL5* in vector and METTL5-overxpressing RPMI-8226 cells. *n* = 3. **B** METTL5 protein expression in RPMI-8226 cells with or without METTL5 overexpression. **C**, **D** The m^6^A modification of total RNA (**C**) and 18S rRNA (**D**) in vector and METTL5-overexpressing RPMI-8226 cells was determined by LC–MS/MS. **E** RNA formaldehyde denaturing gel electrophoresis of RNAs from vector and METTL5-overxpressing RPMI-8226 cells. **F**, **G** Statistical quantification of the expression levels of A_1825_ (**F**) and m^6^A_1832_ (**G**) in 18S rRNA in RPMI-8226 cells with or without METTL5 overexpression. *n* = 3. **H** Growth curves of vector and METTL5-overexpression RPMI-8226 cells as evaluated by CCK-8 assay. *n* = 5. **I** Apoptosis analysis by flow cytometry of vector and METTL5-overexpressing RPMI-8226 cells. *n* = 3. **J**, **K** Cell cycle analysis (**J**) and its quantification (**K**) of vector and METTL5-overexpressing RPMI-8226 cells. *n* = 3. **L** An orthotopical xenograft model was established through the injection of RPMI-8226-vector and RPMI-8226-overexpression cells carrying a luciferase reporter into the tail vein of NSG mice (*n* = 5 in each group) (Created with BioRender.com.). **M**, **N** Whole-body bioluminescence imaging (BLI) (once per week) (**M**) and measurements (**N**) of vector and METTL5-overexpressing mice. **O** Survival curves for vector and METTL5^−^ overexpressing orthotopical xenograft models. **P**, **Q** Representative IHC staining images of METTL5 (**P**) and quantification of the RPMI-8226 MM cells (**Q**) in the bone marrow of vector and METTL5-overexpressing mice. Scale bars: left panels, 200 μm; right panels, 50 μm. Independent biological experiments were repeated at least three times, and the data are presented as the means ± SDs. Statistical differences are indicated by *p*-values of *P < 0.05, **P < 0.01, ***P < 0.001 and ****P < 0.0001.

To clarify the carcinogenic and cancer-promoting role of METTL5, we utilized an overexpression lentivirus to elevate the expression of METTL5 in RPMI-8226 and H929 cells, and conducted functional phenotype detection by qPCR and western blot analyses to confirm overexpression (Fig. 2A, B, supplementary Fig. 2A, B). LC–MS/MS is a key method for the quantitative detection of m^6^A. Indeed, LC–MS/MS detection demonstrated that overexpressing METTL5 increased m^6^A modification in RNA in MM cells, significantly raised the level of m^6^A modification in 18S rRNA, and did not alter the expression level of 18S rRNA (Fig. 2C–E, supplementary Fig. 2C–E). Additionally, high expression of METTL5 upregulated the m^6^A level the most at the A1832 site in 18S rRNA (Fig. 2F, G, supplementary Fig. 2F–K). Functionally, overexpression of METTL5 promoted the proliferation of MM cells and reduced cell apoptosis (Fig. 2H, I, supplementary Fig. 2L–O). Likewise, an increase in the proportion of S phase in cell cycle distribution also reflected the pro-carcinogenic effect of high expression of METTL5 (Fig. 2J, K, supplementary Fig. 2P, Q). To determine the functional role of METTL5 in MM in vivo, we constructed an orthotopical xenograft model by injecting METTL5-overexpressing and vector RPMI-8226 cells carrying luciferase through the tail vein (Fig. 2L) of NSG mice. Interestingly, MM cells in METTL5-overexpressing mice had a faster growth rate (Fig. 2M, N), rapid weight loss (supplementary Fig. 2R, S), and a shorter survival time (Fig. 2O) compared to control animals. Further, both the infiltration of MM cells into the bone marrow of mice carrying overexpressed METTL5 MM cells and the expression level of METTL5 in MM cells were significantly higher than those in vector mice (Fig. 2P, Q). Overall, these data support the essential functions of METTL5 in promoting MM malignant progression in vitro and in vivo.

### Loss of METTL5 inhibits MM progression in vitro and in vivo

**Fig. 3:**
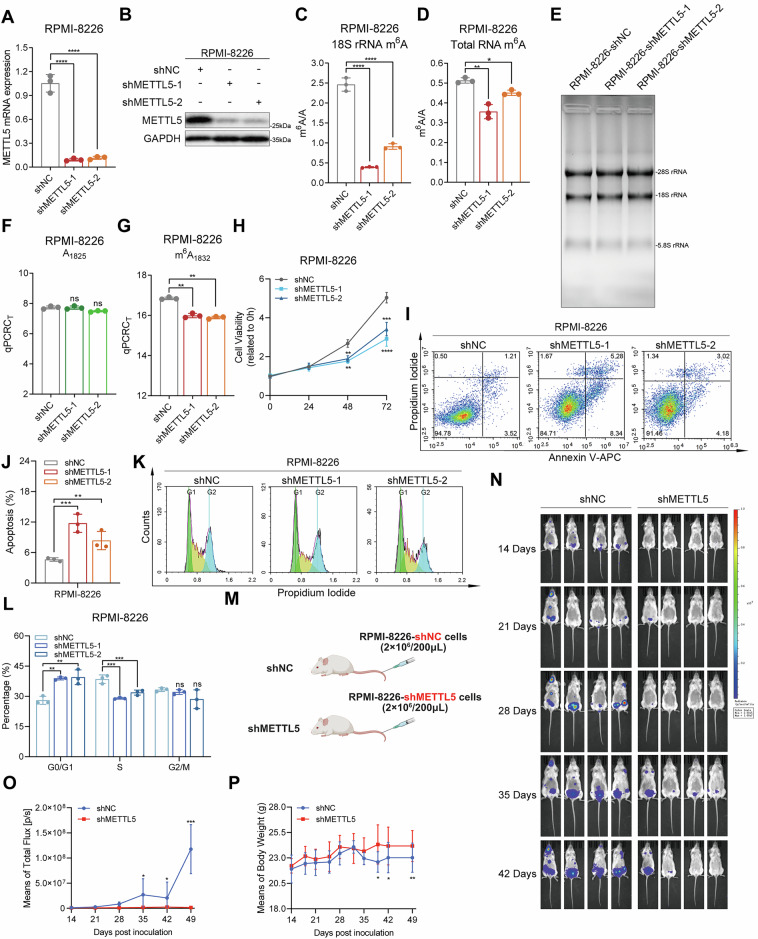
Depletion of METTL5 inhibits MM progression in vitro and vivo. **A** *METTL5* mRNA expression in RPMI-8226 cells with or without METTL5 knockdown. *n* = 3. **B** The protein expression of METTL5 in control and METTL5-knockdown RPMI-8226 cells. **C**, **D** The m^6^A modification of total RNA (**C**) and 18S rRNA (**D**) in control and METTL5-knockdown RPMI-8226 cells was determined by LC–MS/MS. **E** RNA formaldehyde denaturing gel electrophoresis of RNAs from control and METTL5-knockdown RPMI-8226 cells. **F**, **G** Statistical quantification of the expression levels of A_1825_ (**F**) and m^6^A_1832_ (**G**) in 18S rRNA in control and METTL5-knockdown RPMI-8226 cells. *n* = 3. **H** Growth curves of control and METTL5-knockdown RPMI-8226 cells as evaluated by CCK-8 assay. *n* = 5. **I**, **J** Apoptosis analysis (**I**) and its statistical quantification (**J**) of control and METTL5-knockdown RPMI-8226 cells. *n* = 3. **K**, **L** Cell cycle analysis (**K**) and its statistical quantification (**L**) of control and METTL5-knockdown RPMI-8226 cells. *n* = 3. **M** Diagram of control and shMETTL5 orthotopical xenograft model (*n* = 5 in each group) (Created with BioRender.com.). **N**, **O** Whole-body BLI (once per week) (**N**) and measurements (**O**) of control and shMETTL5 mice. **P** Body weight trends in control and shMETTL5-bearing mice. Independent biological experiments were repeated at least three times, and the data are presented as the means ± SDs. Statistical differences are indicated by *p*-values of *P < 0.05, **P < 0.01, ***P < 0.001 and ****P < 0.0001.

To further confirm whether METTL5 acts as an oncogene in MM, we silenced METTL5 expression in RPMI-8226 and U266 cells using two independent short hairpin RNAs (shRNAs) (Fig. 3A, B, Supplementary Fig. 3A, B). METTL5 knockdown reduced the level of m^6^A modification in total RNA and 18S rRNA (Fig. 3C–E, Supplementary Fig. 3C–E), and decreased the expression of m^6^A_1832_ (Fig. 3F, G, Supplementary Fig. 2F–K). In vitro, the depletion of METTL5 resulted in slower proliferation of MM cells and promoted apoptosis (Fig. 3H–J, Supplementary Fig. 3L–N). Cell cycle analysis revealed cell cycle arrest, further, the observed slowing of growth in METTL5 knockdown cells (Fig. 3K, L, Supplementary Fig. 3O, P). Next, we performed in vivo experiments utilizing METTL5 knockdown (shMETTL5-1) and control RPMI-8226 cells to construct MM blood tumor models (Fig. 3M). Compared to control mice, shMETTL5-1 cells grew slower in mice (Fig. 3N, O), had slower weight loss and a longer survival time (Fig. 3P, Supplementary Fig. 2Q). Additionally, compared with control mice, shMETTL5-1 bearing mice had fewer MM cells in their bone marrow, and reduced expression of METTL5 (Supplementary Fig. 3R, S). In summary, inhibiting METTL5 expression halted the malignant progression of MM both in vitro and in vivo. Therefore, targeting METTL5 could serve as a potential method for suppressing MM progression and disease severity.

### SEPHS2 is regulated by METTL5-mediated mRNA translation in MM cells

**Fig. 4:**
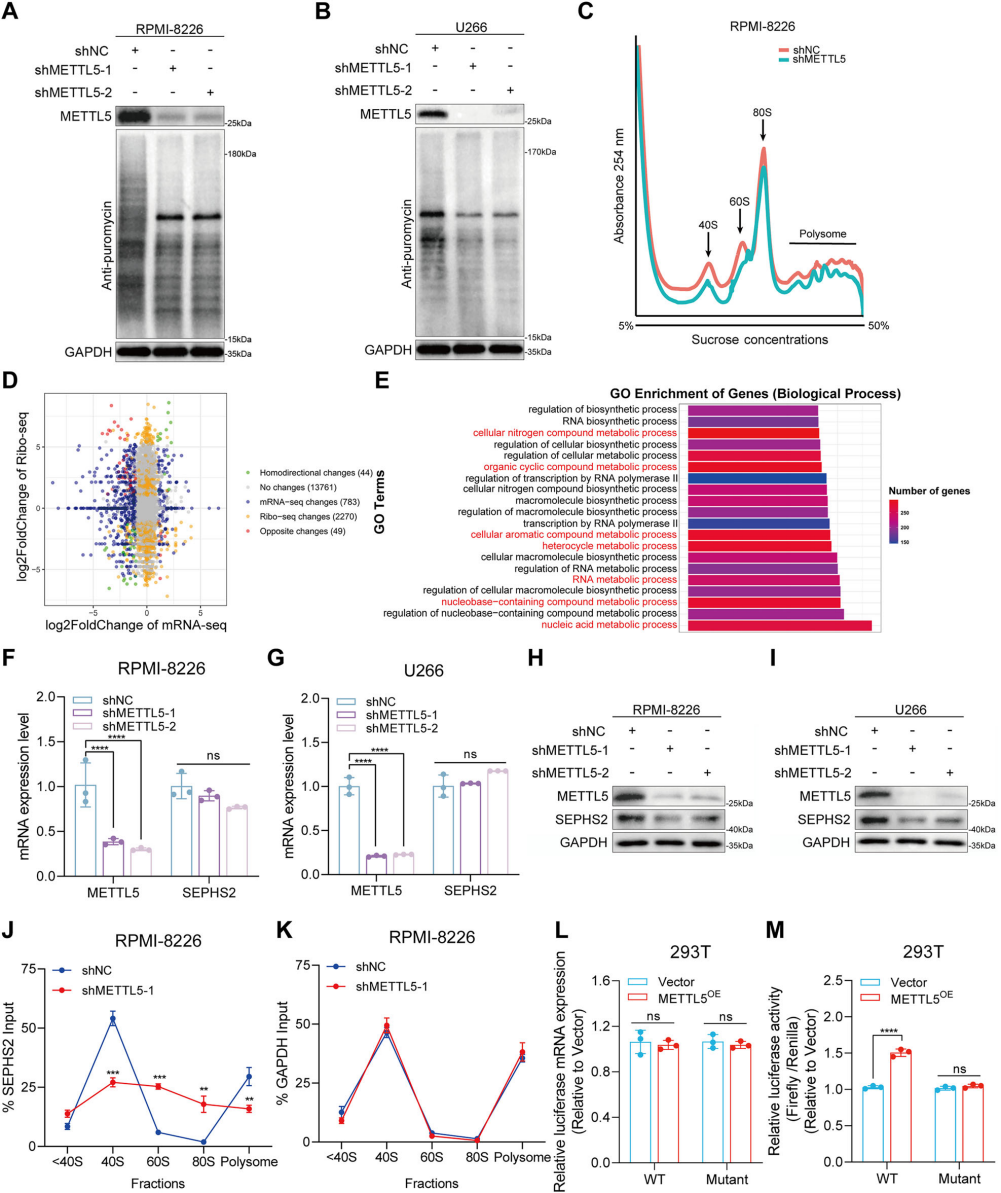
SEPHS2 is regulated by METTL5-mediated mRNA translation in MM cells. **A**, **B** Global translation efficiency of control and METTL5-knockdown RPMI-8226 (**A**) and U266 (**B**) cells was detected by SUnSET assay. **C** Global translation activity of control and METTL5-knockdown RPMI-8226 cells was analyzed by polysome profiling. **D** Scatter plots illustrating differential translation efficiency genes (DTEGs) from Ribo-seq combination RNA-seq analysis upon METTL5 knockdown in RPMI-8226 cells. **E** Biological process (BP) of gene ontology (GO) pathway enrichment analysis of DTEGs. Red: SEPHS2-mediated pathways. **F**, **G** *METTL5* and *SEPHS2* mRNA expression in RPMI-8226 (**F**) and U266 (**G**) cells with or without METTL5 knockdown. *n* = 3. **H**, **I** METTL5 and SEPHS2 protein expression in control and METTL5-knockdown RPMI-8226 (**H**) and U266 (**I**) cells. **J**, **K** *SEPHS2* (**J**) and *GAPDH* (**K**) mRNA expression levels in each fraction of polysome profiling were determined by RT-qPCR in RPMI-8226 cells with or without METTL5 knockdown. *n* = 3. **L** Relative luciferase mRNA expression of wild-type and mutant reporters in vector- or METTL5-overexpressing cells. *n* = 3. **M** Luciferase activity of reporters with wild-type or mutant 5′ UTR sequences transfected with vector or METTL5 overexpression plasmids. The relative luciferase activity in samples was determined by calculating the ratio of firefly luciferase activity over Renilla luciferase activity. *n* = 3. Independent biological experiments were repeated at least three times, and the data are presented as the means ± SDs. Statistical differences are indicated by *p*-values of **P < 0.01, ***P < 0.001 and ****P < 0.0001.

METTL5 primarily impacts the translation efficiency of total proteins by influencing m^6^A modification at the A1832 site of 18S rRNA [8]. As anticipated, the translation efficiency of total proteins in the RPMI-8226 and H929 cells overexpressing METTL5 was significantly increased compared to the control group (Supplementary Fig. 4A, B). Conversely, METTL5-deficient cells were notably less efficient at protein synthesis (Fig. 4A, B). Polysome profiling analysis showed an increase in the loss of polysomes and 40S monosomes in MM cells post METTL5 deletion (Fig. 4C, Supplementary Fig. 4C). 40S monosomes primarily influence mRNA scanning during translation initiation. Thus, our data indicate that reduced scanning efficiency of mRNA translated in 40S monosomes decreased protein translation efficiency in METTL5 knockdown cells.

To further define METTL5-mediated translation regulation and its molecular mechanism in MM progression, we performed transcriptome sequencing (RNA-seq) and ribosome profiling sequencing (Ribo-seq) in RPMI-8226 cells with or without METTL5 knockdown. Ribo-seq analysis indicated a decrease in ribosome-protected RNA fragments (RPFs) (Supplementary Fig. 4D), codon binding efficiency (Supplementary Fig. 4E) and the proportion of reads distributed at the 5′ UTR upon METTL5 depletion (Supplementary Fig. 4F). Cumulatively, these results indicate that METTL5 depletion significantly inhibits the translation efficiency of proteins in MM cells. Upon silencing METTL5 expression, changes were observed in the mRNA and RPFs of numerous genes (Fig. 4D). We conducted enrichment analysis of the biological process (BP) pathway of gene ontology (GO) for differential translation efficiency genes (DTEGs). Post METTL5 depletion, the metabolism of many macromolecular compounds was significantly inhibited, such as nucleic acid metabolic processes, regulation of nucleobase-containing compound metabolic processes, and nucleobase−containing compound metabolic processes. Interestingly, the metabolism of nucleic acids and nitrogen-containing compounds is closely related to the internal protein synthesis in MM cells, and is a key process affecting MM cell growth [18] (Fig. 4E). This suggests that METTL5 mediates specific mRNA translation efficiency to alter the metabolic processes of key macromolecules in cells that affect the progression of MM. Based on our analyses, SEPHS2 expression was most significantly reduced post METTL5 deletion, and was found to be involved in seven of the top 20 inhibited GO biological process pathways (Fig. 4E, Supplementary Table 6). Therefore, we suspect that SEPHS2 may act as a key gene for METTL5 that is regulated by METTL5-mediated translational mechanisms. The expression level of SEPHS2 in our orthotopical xenograft mouse model and in clinical BM samples was consistent with the METTL5 trend (Supplementary Fig. 4G–I). These results suggest that changes in the translation efficiency of specific gene proteins by METTL5 affect MM progression, and SEPHS2 may be the most critical downstream gene.

### METTL5-mediated 40S ribosome recognizes the 5′ TOP motif of SEPHS2 to promote its translation

Next, we aimed to further clarify the specific molecular mechanisms by which METTL5 regulates SEPHS2 translation. Consistent with sequencing results, SEPHS2 mRNA expression did not show significant changes regardless of whether METTL5 expression was silenced (Fig. 4F, G) or overexpressed (Supplementary Fig. 5A, B) in MM cells. However, the protein expression level of SEPHS2 was consistent with changes observed in METTL5 (Fig. 4H, I, Supplementary Fig. 5C, D). These results suggest that METTL5 primarily influences SEPHS2 expression at the level of translation rather than transcription. Polysome profiling is the gold standard for the detection of protein translation efficiency. In accordance with polysome profiling analysis results, the distribution of SEPHS2 in 40S ribosomes and polysomes significantly decreased in METTL5 depleted cells (Fig. 4J, Supplementary Fig. 5E). However, no significant difference was observed in the distribution of control gene GAPDH across each isolated component (Fig. 4K, supplementary Fig. 5F). The 5′UTR is a crucial area that impacts the translation process [19]. Compared with the control group, METTL5 depletion did not influence RNA coverage on the SEPHS2 mRNA track, but METTL5 knockdown led to reduced ribosome footprint occupancy on the SEPHS2 mRNA track (Supplementary Fig. 5G). This reduction was mainly concentrated at the 5′ UTR, aligning with the overall shift in the distribution of reads in exons (Supplementary Fig. 3F). Previous research has indicated that the 5′ terminal oligopyrimidine (5′ TOP) motif plays a critical role in stimulating cell growth by activating the translational machinery, and is also a key structure for the direct or indirect interactions of most methyltransferases with mRNA [20]. As anticipated, multiple 5′ TOP motif structures were identified in the 5′ UTR sequence of the SEPHS2 mRNA exon by query in the NCBI database. The results from the dual-luciferase reporter assay further confirmed that the 5′ TOP motif is a pivotal structure for METTL5 to facilitate SEPHS2 mRNA translation (Fig. 4L-M, supplementary Fig. 5H). Collectively, these findings suggest that METTL5 mediates 40S ribosome-specific scanning of the 5’ TOP motif in the 5’ UTR of SEPHS2, thereby initiating its translation and promoting SEPHS2 expression in MM cells.

### SEPHS2 mediates selenoprotein synthesis to regulate DNA damage-associated apoptosis in MM cells

**Fig. 5:**
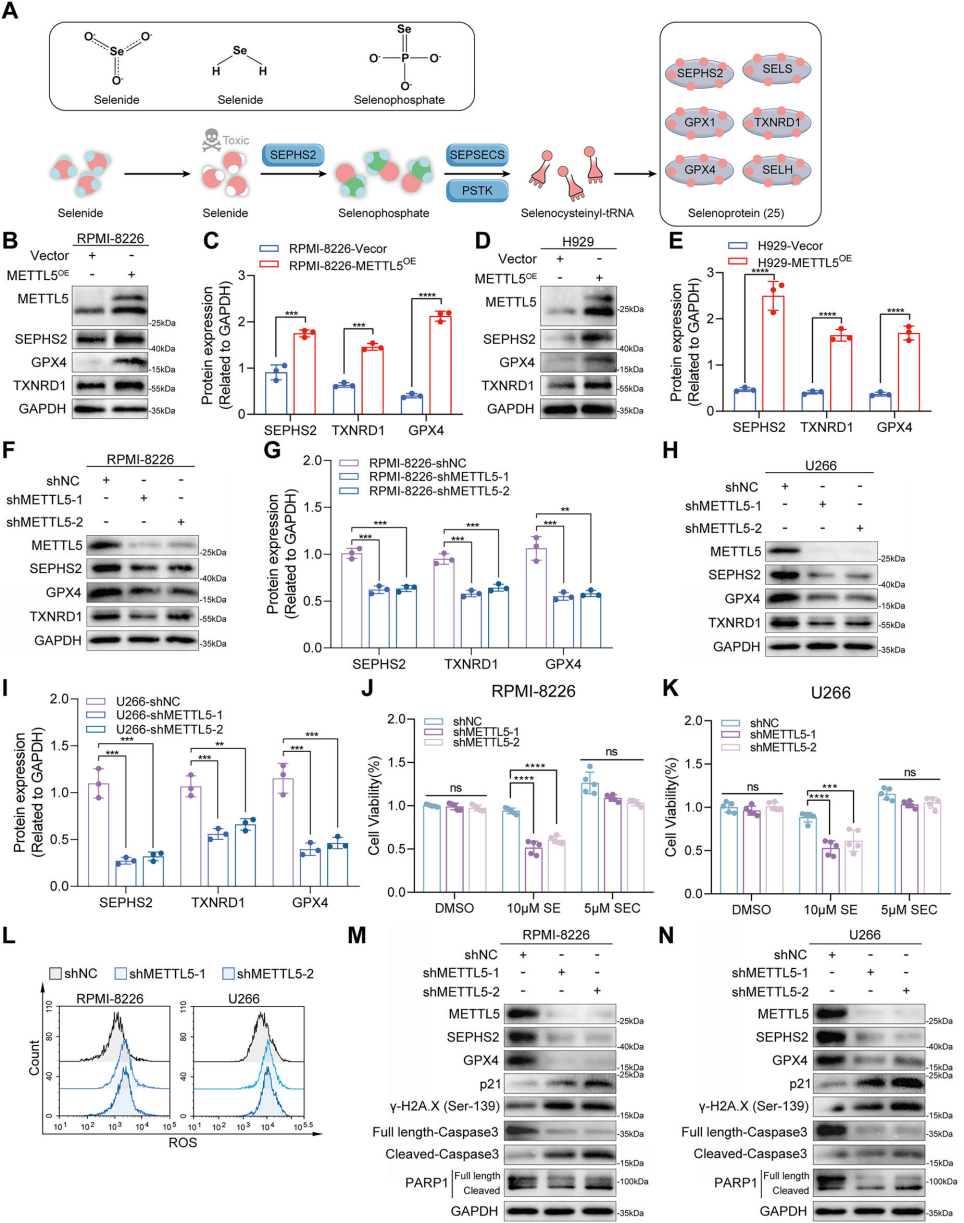
SEPHS2 mediates selenoprotein synthesis to regulate DNA damage-associated apoptosis in MM cells. **A** Diagram of selenium metabolism and selenoprotein synthesis. **B**–**E** The protein expression of METTL5, SEPHS2, GPX4, and TXNRD1 in vector and METTL5-overexpressing RPMI-8226 (**B**, **C**) and H929 (**D**, **E**) cells was analyzed by Western blot and quantitative analysis. (F–I) The protein expression of METTL5, SEPHS2, GPX4 and TXNRD1 in control and METTL5-knockdown RPMI-8226 (**F**, **G**) and U266 (**H**, **I**) cells was analyzed by Western blot and quantitative analysis. **J**, **K** Cell viability of control and METTL5-knockdown RPMI-8226 (**J**) and U266 (**K**) cells following treatment with DMSO, 10 nM selenite (SE), or 5 μM selenocysteine (SEC) for 48 h as evaluated by CCK-8 assay. *n* = 5. **L** Flow cytometry analysis of CellROX-stained control and METTL5-knockdown RPMI-8226 and U266 cells. **M**, **N** The protein expression of METTL5, SEPHS2, GPX4, cyclin-dependent kinase inhibitor p21, DNA damage marker γ-H2A.X (Ser139) and apoptosis-associated proteins caspase3 and PARP1 in control and METTL5-knockdown RPMI-8226 (**M**) and U266 (**N**) cells was analyzed by Western blot. Independent biological experiments were repeated at least three times, and the data are presented as the means ± SDs. Statistical differences are indicated by *p*-values of **P < 0.01, ***P < 0.001 and ****P < 0.0001.

Research has demonstrated that tumor cells exhibit robust selenium uptake capacity [21]. SEPHS2 is a vital enzyme involved in selenoprotein synthesis, capable of converting toxic selenite as substrate into non-toxic selenoprotein (Fig. 5A). Certain selenoproteins, such as glutathione peroxidase 4 (GPX4), have notable antioxidant effects and play a vital role in the homeostasis of tumor cells [22]. To determine whether METTL5 played a role in SEPHS2-mediated control of selenoproteins during MM, we measured protein levels of GPX4 and thioredoxin reductase 1 (TXNRD1) in control and METTL5-overexpressing MM cells. Upon METTL5 overexpression, the expression of SEPHS2 and downstream selenoproteins GPX4 and TXNRD1 increased (Fig. 5B–E). In contrast, SEPHS2, GPX4, and TXNRD1 were downregulated following METTL5 silencing (Fig. 5F–I). Consistent with previously published reports, SEPHS2 protein expression was diminished after METTL5 depletion and failed to alleviate the toxicity of sodium selenite (SE) to MM cells [21]. Addition of SEPHS2’s product, selenocysteine (SEC), did not adversely impact the growth of METTL5 knockdown MM cells, but rather promoted MM cell growth (Fig. 5J, K). These findings suggest that METTL5 fosters the progression of MM by mediating the activation of selenium metabolism pathways via enhanced SEPHS2 translation.

SEPHS2, involved in the synthesis of the selenoprotein GPX4, plays a critical role in regulating oxidative stress and maintaining intracellular redox homeostasis [23]. As anticipated, the silencing of METTL5 in MM cells triggered an increased oxidative stress response (Fig. 5L). DNA damage is considered the most significant cause of tumor cell death resulting from oxidative stress [24]. The depletion of METTL5 decreased the expression of SEPHS2 and GPX4. Increased DNA damage can lead to increased expression of cyclin-dependent kinase inhibitor p21 and DNA damage marker γ-H2A.X and induce cell apoptosis, leading to the activation of apoptotic proteins (caspase3, caspase7, poly (ADP-ribose) polymerase 1 (PARP1), etc.). As expected, the depletion of METTL5 elevated levels of γ-H2A.X, decreased caspase3 and PARP1 protein levels, and increased the cleaved protein (Fig. 5M, N). In conclusion, depletion of METTL5 leads to a decrease in SEPHS2 expression and subsequent selenoprotein synthesis, which promotes oxidative stress-mediated DNA damage and inhibits MM progression.

### Depletion of METTL5 inhibits MM progression through partial reduction of SEPHS2 and subsequent inhibition of selenium metabolism

**Fig. 6:**
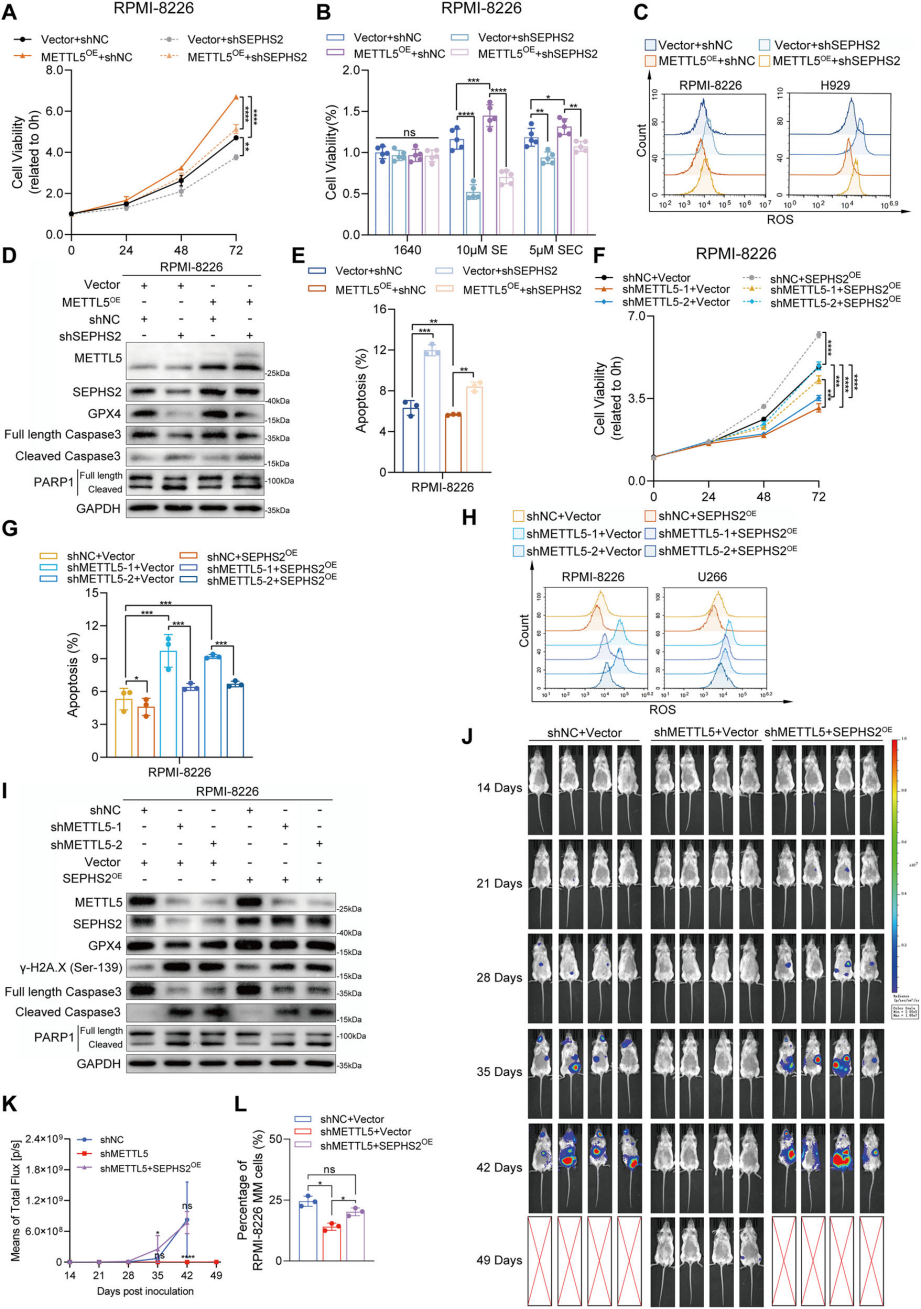
METTL5 promotes the progression of MM by regulating the expression of SEPHS2 and downstream selenium metabolism. **A** Growth curves of vector and METTL5-overexpressing RPMI-8226 cells with or without SEPHS2 knockdown as evaluated by CCK-8 assay. *n* = 5. **B** CellRox-stained vector and METTL5-overexpressing RPMI-8226 and H929 cells, with or without SEPHS2 knockdown, as determined by flow cytometry. *n* = 3. **C** Cell viability of vector and METTL5-overexpressing RPMI-8226 cells, with or without SEPHS2 knockdown, following treatment with DMSO, 10 nM SE or 5 μM SEC for 48 h as evaluated by CCK-8 assay. *n* = 5. **D** The protein expression of METTL5, SEPHS2, GPX4, γ-H2A.X (Ser139), caspase3, and PARP1 in vector and METTL5-overexpressing RPMI-8226 cells with or without SEPHS2 knockdown was analyzed by Western blot. **E** Statistical quantification of apoptosis in vector and METTL5-overexpressing RPMI-8226 cells with or without SEPHS2 knockdown. *n* = 3. **F** Growth curves of control and METTL5-depleted RPMI-8226 cells with or without SEPHS2 restoration as evaluated by CCK-8 assay. *n* = 5. **G** Statistical quantification of apoptosis in control and METTL5-depleted RPMI-8226 cells with or without SEPHS2 restoration. *n* = 3. **H** CellROX-stained control and METTL5-depleted RPMI-8226 and U266 cells with or without SEPHS2 restoration measured by flow cytometry. **I** The protein expression of METTL5, SEPHS2, GPX4, γ-H2A.X (Ser139), caspase3, and PARP1 in control and METTL5-depleted RPMI-8226 cells with or without SEPHS2 restoration was analyzed by Western blot. **J** Whole-body BLI (once per week) of shNC (native control)/vector, shMETTL5/vector, and shMETTL5/SEPHS2- overexpressing mice. **K** The measurements of whole-body BLI of shNC/vector, shMETTL5/vector, and shMETTL5/SEPHS2^OE^ mice. **L** Quantification of the RPMI-8226 MM cells for each group of orthotopical xenograft models. Independent biological experiments were repeated at least three times, and the data are presented as the means ± SDs. Statistical differences are indicated by *p*-values of *P < 0.05, **P < 0.01, ***P < 0.001 and ****P < 0.0001.

In an effort to further clarify the specific mechanism by which METTL5 influences MM progression via SEPHS2, we both reduced and increased SEPHS2 expression in MM cells overexpressing and silencing METTL5 and monitored changes in MM phenotypes. Specifically, we stably transfected METTL5-overexpressing RPMI-8226 and H929 MM cells using SEPHS2 interference or control sequences. As METTL5 facilitates translation, significant inhibition of SEPHS2 mRNA levels in METTL5-overexpressing cells only partially inhibited its protein expression as expected (Supplementary Fig. 6A–D). Similarly, overexpression of SEPHS2 in METTL5-knockdown MM cells did not achieve the same overexpression efficiency at the protein level as mRNA (Supplementary Fig. 7A–D). Silencing SEPHS2 partially, but not completely, inhibited cell growth (Fig. 6A, Supplemental Fig. 6E) and reduced cytotoxicity of SE (Fig. 6B, Supplementary Fig. 6F) and the oxidative stress response (Fig. 6C) caused by METTL5 overexpression. Further, depletion of SEPHS2 partially decreased the activity of the selenium metabolic pathway triggered by overexpression of METTL5, and promoted DNA damage, activated apoptotic proteins (Fig. 6D, Supplementary Fig. 6G), and promoted apoptosis (Fig. 6E, Supplementary Fig. 6H–J) in METTL5-overexpressing MM cells.

Next, we evaluated the effect of SEPHS2 overexpression in METTL5-knockdown MM cells. In vitro, restoring SEPHS2 expression in stably METTL5-depleted MM cells partially rescued the growth inhibition (Fig. 6F, Supplementary Fig. 7E) and increased apoptosis (Fig. 6G, Supplementary Fig. 7F–H) induced by METTL5 knockdown. Restoring SEPHS2 expression also elevated the activity of the selenium metabolism pathway in METTL5-knockdown MM cells (Supplementary Fig. 7I, J), and mitigated the oxidative stress response (Fig. 6H), DNA damage response, and reduced the activation of apoptotic proteins (Fig. 6I, Supplementary Fig. 7K). Consistently, restoration of SEPHS2 partially mitigated the effect of METTL5 depletion, resulting in a worsened MM burden (Fig. 6J, K) and weight loss (Supplementary Fig. 8B, C) in the orthotopical xenograft model (Supplementary Fig. 8A). Compared with the control group, the number of MM cells and the IHC staining of METTL5, SEPHS2, and GPX4 decreased in the bone marrow of mice receiving cells with METTL5 deletion. However, upon restoration of SEPHS2, the number of MM cells and the activities of SEPHS2 and GPX4 significantly increased, except for METTL5 expression (Fig. 6L and Supplementary Fig. 8D). In conclusion, our results suggest that depletion of METTL5 inhibits MM progression partially by suppressing SEPHS2-mediated selenium metabolism both in vivo and in vitro.

### Salvianolic acid C is a METTL5 inhibitor with significant cytotoxic effects on MM cells

**Fig. 7:**
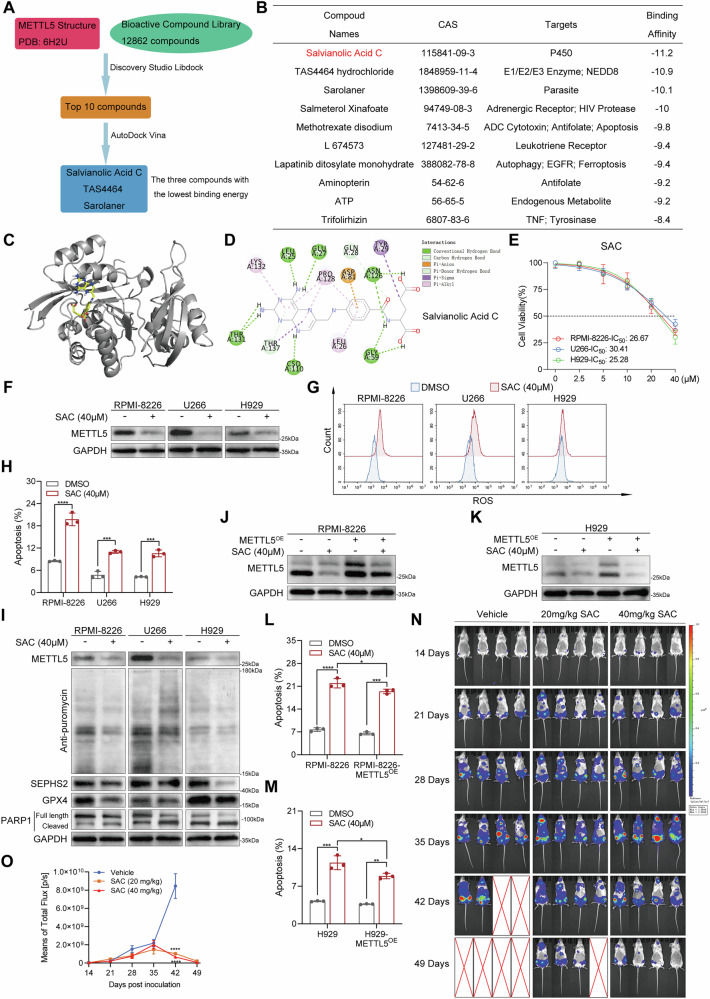
SAC is a potential METTL5 inhibitor with anti-MM effects in vitro and in vivo. **A** Diagram of the high-throughput virtual screening method used for METTL5 inhibitor identification. **B** The top 10 potential METTL5 inhibitors with the lowest binding energy. **C** 3D structure of SAC binding located within the active pocket of METTL5. **D** Interactions between SAC and Thr131, Lys132, Thr137, Leu25, Cso110, Glu27, Pro128, Leu26, Asp81, Gln28, Asn126, Gly59, and Tyr29 within the active pocket of METTL5. **E** IC_50_ values of SAC in U266, RPMI-8226, and H929 cells determined by CCK-8 assay. **F** Western blot analysis of METTL5 protein expression in U266, RPMI-8226, and H929 cells treated with either DMSO or 40 μM SAC for 48 h. **G** Flow cytometry analysis of CellROX-stained U266, RPMI-8226, and H929 cells treated with either DMSO or 40 μM SAC for 48 h. **H** Statistical quantification of apoptosis in U266, RPMI-8226, and H929 cells post-treatment with either DMSO or 40 μM SAC for 48 h. *n* = 3. **I** Western blot analysis of METTL5, SEPHS2, GPX4, and PARP1 protein levels, and global protein translation efficiency in U266, RPMI-8226, and H929 cells treated with either DMSO or 40 μM SAC for 48 h. **J**, **K** Western blot analysis of METTL5 protein expression in RPMI-8226 (**J**) and H929 (**K**) cells with or without METTL5 overexpression following treatment with either DMSO or 40 μM SAC for 48 h. **L**, **M** Statistical quantification of apoptosis in RPMI-8226 (**L**) and H929 (**M**) cells with or without METTL5 overexpression following treatment with either DMSO or 40 μM SAC for 48 h. *n* = 3. **N**, **O** Whole-body BLI (once per week) (**N**) and measurements (**O**) of orthotopical xenograft models receiving either SAC (20 mg/kg or 40 mg/kg) or vehicle control treatment. Independent biological experiments were repeated at least three times, and the data are presented as the means ± SDs. Statistical differences are indicated by *p*-values of *P < 0.05, **P < 0.01, ***P < 0.001 and ****P < 0.0001.

Given the key role of METTL5 in regulating apoptosis in MM, we attempted to identify small-molecule compounds targeting METTL5 to determine the clinical feasibility of targeting METTL5 as a treatment for MM. Subsequently, we used the METTL5 protein (PDB: 6H2U) (Supplementary Fig. 9A) downloaded from the Protein Data Bank (PDB) website (https://www.rcsb.org/) as the receptor and performed structure-based virtual screening with 12,862 compounds of the TargetMol Bioactive Compound Library (Fig. 7A). The docking result shows that among the 12654 compound conformations, 495775 docking conformations successfully docked into the protein pocket. We identified the most likely METTL5 inhibitors using Discovery Studio Libdock and AutoDock Vina, with the top 10 compounds ranked by binding power shown in Fig. 7B. Interestingly, salvianolic Acid C (SAC) had the highest binding power. Further, we analyzed the binding pattern of SAC and generated related 3D/2D figures, predicting that it binds to the active structural domain of METTL5 with 9 H-bonds, 7 Pi-bonds, and 2 H-pi-bonds (Fig. 7C, D).

Next, to define the effectiveness of SAC in inhibiting METTL5 expression and treating MM, MM cell lines were exposed to SAC treatment. As expected, SAC exhibited potent cytotoxicity when treating RPMI-8226, U266, and H929 MM cells (Fig. 7E). When SAC was directly added to MM cell cultures, the expression of METTL5 protein in MM cells was inhibited to varying degrees, along with similar changes in the expression of 18S rRNA m^6^A_1832_ (Fig. 7F, Supplementary Fig. 9B, C). Concurrently, SAC treatment also promoted an increase in ROS levels (Fig. 7G) and apoptosis (Fig. 7H, Supplementary Fig. 9D–F) in MM cells. Consistent with METTL5 knockdown, SAC treatment induced a significant decrease in global protein translation efficiency, as well as SEPHS2, GPX4, and full-length PARP1 protein levels in treated MM cells (Fig. 7I). To further ascertain the specificity of SAC’s targeting of METTL5, we performed rescue experiments through overexpression of METTL5. Strikingly, we found that overexpression of METTL5 post-SAC treatment could partially rescue the level of METTL5 protein (Fig. 7J, K) and 18S rRNA m^6^A_1832_ expression (Supplementary Fig. 9G–J), accompanied by a corresponding change in the protein expression of SEPHS2 and GPX4 (Supplementary Fig. 9K, L). SAC effectively suppresses the proliferation of METTL5-overexpressing MM cells (Supplementary Fig. 9M, N). Furthermore, overexpression of METTL5 also enhances the drug resistance of MM cells to SAC (Supplementary Fig. 9O). Overexpression of METTL5 also reduced the ROS levels and apoptosis induced by SAC treatment in RPMI-8226 and H929 cells (Fig. 7L, M, Supplementary Fig. 9P–R).

**Fig. 8:**
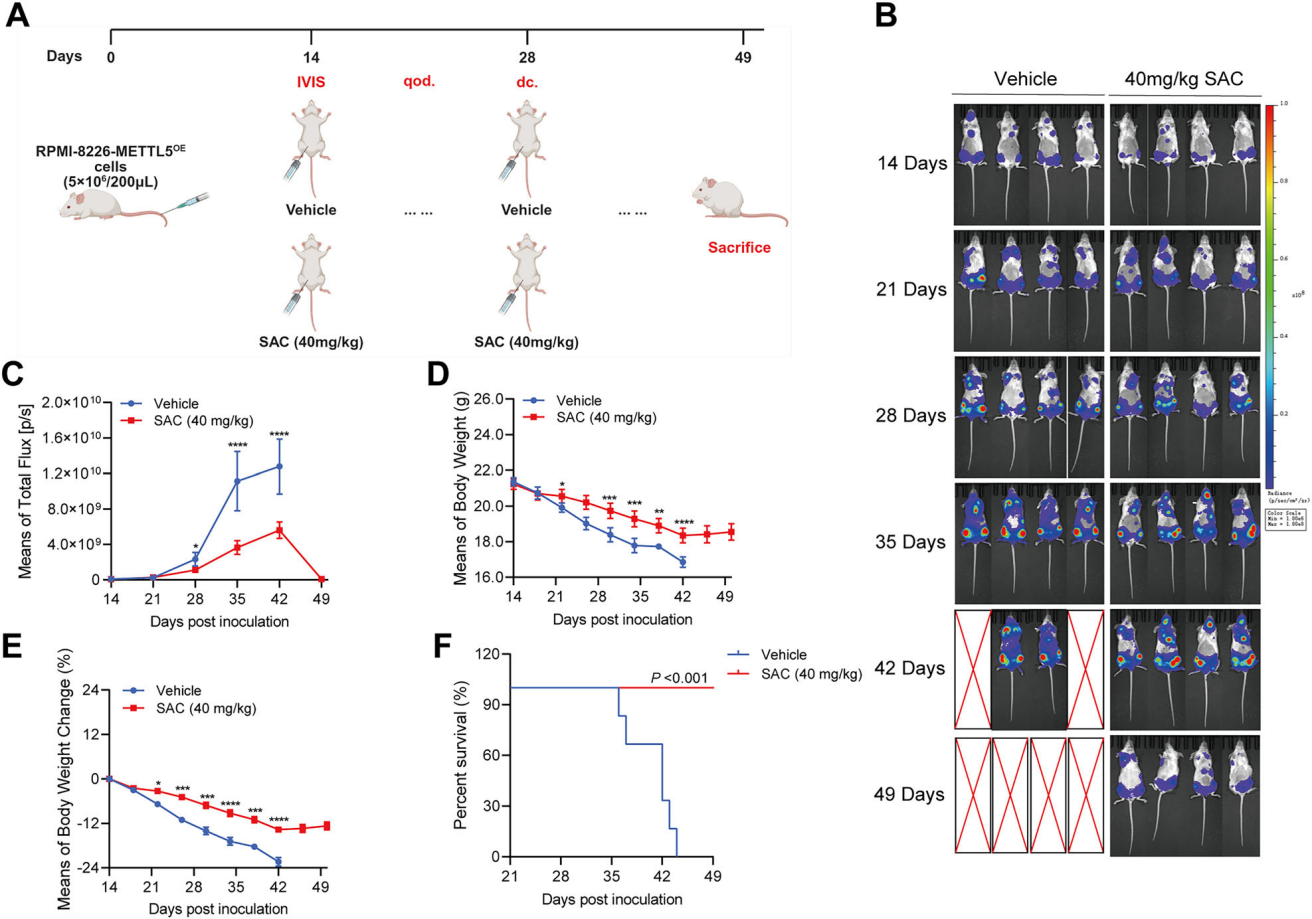
SAC can effectively inhibit the growth of MM with high expression of METTL5 in vivo. **A** An orthotopical xenograft model was established through the injection of RPMI-8226-METTL5^OE^-Luc cells into the tail vein of NSG mice (Created with BioRender.com.). **B**, **C** Whole-body BLI (once per week) (**B**) and measurements (**C**) of orthotopical xenograft models receiving either 40 mg/kg SAC or vehicle control treatment. **D**, **E** Body weight trends (**D**) and average body weight change trends (**E**) for each group of orthotopical xenograft models. **F** Survival curves for orthotopical xenograft models receiving either SAC (20 mg/kg or 40 mg/kg) or vehicle control treatment. Independent biological experiments were repeated at least three times, and the data are presented as the means ± SDs. Statistical differences are indicated by *p*-values of *P < 0.05, **P < 0.01, ***P < 0.001 and ****P < 0.0001.

Given the excellent efficacy of SAC in inducing MM cell death in vitro, we assessed the effectiveness of SAC targeting METTL5 in an orthotopical xenograft model of MM established by injecting RPMI-8226-Luc cells (Supplementary Fig. 10A). Utilizing BLI to observe the dynamic changes of tumor cells in vivo, we observed that SAC treatment markedly reduced MM burden in treated mice (Fig. 7N, O). Specifically, the most pronounced differences in MM burden appeared after 42 days, with treated animals having reduced weight loss (Supplementary Fig. 10B, C) and an improved overall survival rate (Supplementary Fig. 10D). IHC staining studies revealed that SAC treatment significantly suppressed the activity of METTL5, SEPHS2, and GPX4 in BM samples from treated mice compared to control animals (Supplementary Fig. 10E). H&E staining revealed no significant morphological alterations in the major organs of the mice (Supplementary Fig. 10F), indicating no overt toxicity associated with treatment. To further validate the specificity of SAC toward METTL5 in MM cells, we administered SAC in an in vivo model with high METTL5 expression (Fig. 8A). The results demonstrated that SAC significantly suppressed the growth of METTL5^OE^ MM cells in NSG mice and enhanced the survival of the treated animals (Fig. 8B–F). Overall, using SAC to target METTL5 could be an effective and safe strategy for MM treatment.

## Discussion

MM is a malignant plasma cell tumor that currently remains incurable [1]. Despite various treatments, including therapeutic drugs and transplantation for MM, poor efficacy leaves room for improvement in patient response rates [4]. New anti-MM therapies, including chimeric antigen receptor T-cell (CAR-T) therapy and bispecific antibodies, are currently under clinical investigation [25]. However, there is no targeted therapy approved for MM. N6-methyladenosine (m^6^A) is the most prevalent internal chemical modification in eukaryotic RNA [6]. Mounting evidence suggests that m^6^A “writers” are upregulated in cancer, leading to dysregulation of m^6^A modification to promote tumorigenesis, malignant progression, and poor prognosis [26, 27]. In this study, we identify that the m^6^A “writer” METTL5 is upregulated in MM and is associated with the onset and adverse prognosis of MM. Recent studies have reported the critical role of METTL5 in promoting the progression of various cancers, including gastric, liver, cholangiocarcinoma, and colorectal cancer [9, 28–30]. In line with other studies, our study found that METTL5 differs from other m^6^A “writers”. Specifically, METTL5 does not regulate m^6^A modifications of mRNA, but rather consistently promotes m^6^A modification at the A1832 site of 18S rRNA [31, 32]. Functional experiments demonstrated that upregulating and silencing METTL5 expression significantly promoted and inhibited MM progression in vivo and in vitro, respectively, further confirming that METTL5 is a key oncogene in MM.

Increasing evidence suggests that translation, rather than transcription and post-transcriptional modification, is the most crucial step in cellular biological processes within the central dogma [33]. Abnormal protein translation within tumor cells is common amongst various cancers [33, 34]. The decoding center is where the transfer RNA (tRNA) anticodon interacts with the mRNA codon during the translation process [33, 34]. Site m^6^A_1832_, located only a few bases away from this decoding center, can form non-classical base pairing with rRNA modification Cm1703 to bring the ribosomal RNA (rRNA) of the decoding center closer to the mRNA substrate [32, 35]. Moreover, m^6^A_1832_ can accelerate 40S ribosome maturation, promote 40S ribosome scanning of mRNA, facilitate translation initiation, enhanced mRNA stability, and increase translation efficiency [36–39]. In our study, we confirmed that METTL5 improves the overall protein translation efficiency in MM cells by promoting 18S rRNA m^6^A modification. Further, METTL5 deficiency resulted in a decrease in the distribution of the 40S ribosome, a reduction in the binding force between the codon and mRNA, and a decrease in copy number distribution closely related to translation in the 5′UTR. Ultimately, this led to inhibition of the translation process in MM cells. Enrichment analysis of DTEGs after METTL5 deficiency found that RNA and many macromolecule metabolisms in MM cells were inhibited. Interestingly, SEPHS2 was identified as one of the most significantly downregulated DTEGs in terms of translation efficiency and participates in multiple of the inhibited pathways. The 5′TOP motif is a key structure regulating the selective translation of mRNA in the 5′UTR, and the 40S ribosome can selectively stabilize mRNA with the 5′TOP motif, thereby improving its translation efficiency [36]. Similarly, this study confirmed that METTL5 enhances the m^6^A modification of 18S rRNA A1832, accelerates the 40S ribosome’s scanning of the 5′TOP motif of SEPHS2, increases its translation efficiency, and upregulates the protein levels of SEPHS2 in MM cells rather than the mRNA level. The above results indicate that METTL5 promotes the increased expression of SEPHS2 at the translational level by specifically regulating the recognition of SEPHS2 with a specific 5′TOP motif by the 40S ribosome.

Recent research has found that tumor cells, unlike healthy cells, exhibit a strong preference for selenium [15]. However, the toxicity of selenites promotes the death of tumor cells [40]. SEPHS2 is the rate-limiting enzyme that converts selenite to selenocysteine, a key substance in the synthesis of many selenium proteins such as glutathione peroxidase, thioredoxin reductase, and formate dehydrogenase [16]. These selenium proteins are crucial in maintaining cellular redox homeostasis and promoting cell growth [15, 17, 40]. Redox reactions participate in multiple metabolic pathways within cells [41, 42]. Our study found that the downregulation of SEPHS2 caused by METTL5 deficiency results in the blockage of SEPHS2-related metabolic pathways in MM cells, thus confirming this phenomenon. GPX4, one of the essential selenium proteins synthesized by SEPHS2, has been shown in numerous studies to be a key regulator of ferroptosis and an important molecule regulating oxidative stress [43]. GPX4 can inhibit the onset of oxidative stress and reduce DNA damage-related apoptosis [44, 45]. In this study, GPX4 also promotes MM progression through this mechanism. Further, we demonstrated via rescue experiments that METTL5 deficiency inhibits MM progression by partially reducing SEPHS2 and subsequent inhibition of selenium metabolism. The most common metabolic reprogramming, including glucose metabolism and lipid metabolism, plays a key role in the onset and development of cancer [46–48]. Conversely, research on selenium metabolism is less common [15, 21]. For the first time, our findings reveal that METTL5 promotes MM progression through SEPHS2-mediated selenium metabolic reprogramming. Notably, our study identified for the first time that selenoprotein and selenium metabolic synthesis are downstream metabolic pathways in which METTL5 functions.

Currently, induction of cell death is a pharmacological mechanism of many MM treatments [49]. For instance, the proteasome inhibitor bortezomib induces apoptosis in MM cells by promoting ubiquitin hydrolysis [18]. Suppressing protein synthesis at its source also presents a viable strategy for treating MM. Existing research has confirmed that inhibiting protein translation is highly effective against myeloma, which aligns with our findings that suppressing METTL5 and its mediated protein translation effectively curbs MM progression [50, 51]. Hence, targeting METTL5 for MM treatment is a highly promising approach, though no inhibitors targeting METTL5 are currently available. In an effort to further evaluate the potential clinical feasibility of targeting METTL5 as a treatment modality for MM, we successfully identified SAC as an inhibitor targeting METTL5 through structure-based virtual screening. SAC has now been applied in the treatment of other diseases, such as cerebral ischemic injury, SARS-CoV-2, and acute kidney injury; however, its application in cancer has not been reported [52–54]. We validated that SAC effectively inhibits the expression of METTL5 in MM cells and its mediated m^6^A modification at the A1832 of 18S rRNA, and reduces the global protein translation efficiency. In line with the results of our METTL5 knockdown, in vitro experiments showed that SAC induces DNA damage-related apoptosis in MM cells by targeting METTL5 to inhibit SEPHS2, and subsequently, the activity of the selenium metabolism pathway. This effect was more pronounced in MM cells with upregulated METTL5 expression. Consistently, SAC effectively kills MM cells in the orthotopical xenograft model, hinders BM infiltration of MM cells, and prolongs mouse survival time, especially with high concentrations of SAC. More importantly, SAC did not produce toxicity in mouse organs. Given the effectiveness and safety of SAC in vitro and in vivo in treating MM by targeting METTL5 to inhibit protein translation efficiency, we suggest that SAC may be a promising drug for treating MM patients, particularly those exhibiting high METTL5 expression. The utilization of SAC as a potential drug for MM targeted therapy would provide a new basis for this therapy in the field of cancer therapy.

## Conclusion

In summation, this study has decisively illustrated that METTL5 serves as a pivotal gene contributing to the survival and advancement of MM, thereby marking it as a potential target for MM treatment. SAC, acting as an inhibitor of METTL5, can efficaciously impede MM growth and stands as a potential medication for MM treatment, offering innovative concepts for targeted therapy in MM.

## Supplementary information

Supplemental Methods, Figures and Tables

original data

## Data Availability

GSE294608 can be acquired from the Gene Expression Omnibus (GEO) datasets (https://www.ncbi.nlm.nih.gov/geo/). The data supporting the findings of this study are available from the corresponding author upon reasonable request.

## Code availability

Scripts and codes are available upon request.
