# Supplementary material for: METTL5 regulates SEPHS2-mediated selenoprotein synthesis to promote multiple myeloma survival and progression
Source: Cell Death Dis. 2025 Aug 2;16(1):585. doi: 10.1038/s41419-025-07904-6 (PMC12316883; doi:10.1038/s41419-025-07904-6)
Supplement: Supplementary file 1 — Supplemental Methods, Figures and Tables [file 41419_2025_7904_MOESM1_ESM.docx]

Supplementary file for

**METTL5 regulates SEPHS2-mediated selenoprotein synthesis to promote multiple myeloma survival and progression**

Junyao Jiang^1^*, Fangmin Zhong^1^*, Zuomiao Xiao^2^*, Fangyi Yao^1^, Jing Liu^1^, Meiyong Li^1^, Huang Zeng^1^, Yuxiang Qiu^1^, Jing Zhang^1^, Haibin Zhang^1^, Shuqi Li^1^, Ting huang^3^, Wenli Feng^4^, Zhenglan Huang^4^, Bo Huang^1#^, Xiaozhong Wang^1#^

^1^Jiangxi Province Key Laboratory of Immunology and Inflammation, Jiangxi Provincial Clinical Research Center for Laboratory Medicine, Department of Clinical Laboratory, The Second Affiliated Hospital, Jiangxi Medical College, Nanchang University, Nanchang, Jiangxi, P. R. China.

^2^Department of Clinical Laboratory, The Affiliated Ganzhou Hospital of Nanchang University, Ganzhou, Jiangxi, P. R. China.

^3^Department of Blood Transfusion, The Second Affiliated Hospital, Jiangxi Medical College, Nanchang University, Nanchang, Jiangxi, P. R. China.

^4^Department of Clinical Hematology, Key Laboratory of Laboratory Medical Diagnostics Designated By Ministry of Education, School of Laboratory Medicine, Chongqing Medical University, Chongqing, P. R. China.

*Junyao Jiang, Fangmin Zhong and Zuomiao Xiao contributed equally to this work.

Correspondence

Xiaozhong Wang, Jiangxi Province Key Laboratory of Immunology and Inflammation, Jiangxi Provincial Clinical Research Center for Laboratory Medicine, Department of Clinical Laboratory, The Second Affiliated Hospital, Jiangxi Medical College, Nanchang University, Nanchang 330000, Jiangxi, P. R. China.

Email: wangxiaozhong@ncu.edu.cn.

Bo Huang, Jiangxi Province Key Laboratory of Immunology and Inflammation, Jiangxi Provincial Clinical Research Center for Laboratory Medicine, Department of Clinical Laboratory, The Second Affiliated Hospital, Jiangxi Medical College, Nanchang University, Nanchang 330000, Jiangxi, P. R. China.

Email: 764019522@qq.com.

**Supplementary Material and Methods**

**Cell culture**

Human NCI-H929 (RRID: CVCL_1600), RPMI-8226 (RRID: CVCL_0014), U266 (RRID: CVCL_0566), MM1.S (RRID: CVCL_8792), HS-5 (RRID: CVCL_3720) and 293T (RRID: CVCL_0063) cells were purchased from the American Type Culture Collection (ATCC, USA). NCI-H929, RPMI-8226, U266 and MM1.S cells were cultured in RPMI-1640 (Gibco, USA) media, HS-5 and 293T cells were cultured in DMEM (Gibco, USA) media supplemented with 10% fetal bovine serum (Gibco, USA). Cells were grown in a 5% CO2 cell culture incubator (Thermo Scientific, USA) at 37°C. The cell lines used in this study were authenticated by STR profiling within the past 6 months. Regular mycoplasma testing using PCR-based assays confirmed no contamination.

**RNA isolation, reverse transcription, and RT-qPCR**

Total RNA was isolated using RNAiso Plus reagent (Takara, China), and reverse transcribed using the PrimeScript™ RT reagent Kit with gDNA Eraser (Perfect Real Time) (Takara, China) following the manufacturer’s protocol. Real-time quantitative PCRs (qPCR) were performed using TB Green® Premix Ex Taq™ II (Tli RNaseH Plus) (Takara, China). To calculate fold changes in expression of the indicated genes, the comparative CT method (2^−ΔΔCt^) was used, with GAPDH utilized as the loading control. Primer sequences are provided in supplemental Table 3.

**Western blot**

Total protein from BM samples and cultured cells was extracted with RIPA lysis buffer (Solarbio, China). After the detection of protein concentrations of each sample by BCA assay (ThermoFisher, USA), equal amounts of protein extracts were separated using 10% or 12.5% PAGE Gel Fast Preparation Kits (EpiZyme, China) followed by transfer to a polyvinylidene fluoride (PVDF) membrane (Millipore‐Sigma, USA). Membranes were blocked with 5% nonfat dry milk and incubated with primary antibodies at 4°C overnight, followed by incubation with secondary antibodies conjugated to horseradish peroxidase at room temperature for 2h. Visualization of indicated proteins was detected with the Chemistar High-sig ECL Western blotting substrate (NCM Biotech, China). A list of the primary and secondary antibodies used are included in supplemental Table 4.

**Single‐base elongation- and ligation‐based PCR amplification method (SELECT)**

For quantitatively detecting the m^6^A modification in A1825 (as input control) and A1832 (target point) locus of 18S rRNA, the SELECT method was performed as described^21^. In brief, we used 2μg total RNA extracted from different cells, 400 nM Up/Down primer (Sangon Biotech, China), and 100 μM dNTP (NEB, UK) in 1X CutSmart buffer (NEB, UK) in 17 μl of reaction mixture, which were annealed using a temperature gradient. Then, 3 μl of 0.01 U Bst 2.0 DNA polymerase (NEB, UK), 0.5 U SplintR ligase (NEB, UK), and 10 nM ATP (NEB, UK) were added in the former reaction mixture and incubated at 40°C for 20 min, denatured at 80°C for 20 min, and held at 4°C. Next, 2 μl of elongated and ligated products were used as a template to perform RT-qPCR amplification. Ct values were used to assess m^6^A modification levels. The primers used in this study are listed in supplemental Table 3.

**Cell viability**

A Cell Counting Kit-8 (CCK8; APExBIO, USA) was used to evaluate cell viability. Briefly, 2000 normal or transfected MM cells/well were seeded into 96-well plates. Then, 10 μl CCK8 reagent was added to each sample at 0, 24, 48 and 72h timepoints, and cultured for 2.5 h. In studies assessing selenium metabolic pathway activity, the CCK-8 assay was performed as described above. Briefly, 2×103 MM cells were plated into 96-well plates containing DMSO, Sodium Selenite (SE) or selenocysteine (SEC), and cultured for 48h. Then, 10 μl of CCK8 reagent was added to each sample and cultured for 2.5 h. After the incubation period, absorbance was measured at 450 nm by a microplate reader to evaluate cell viability.

**Immunohistochemical staining assay**

Clinical and mouse BM smear samples were used to perform IHC (Immunohistochemistry Kit, Cell Biological, China) assays. Specifically, endogenous peroxidase was blocked with 3% H2O2 for 10 min at room temperature after deparaffinization and rehydration. Then, antigens were retrieved using the microwave method with citrate, and nonspecific binding was blocked with 5% BSA buffer. Next, tissues were incubated with primary antibodies at 4°C overnight, followed by incubation with secondary antibodies at room temperature for 2h. The antibodies used in the IHC assays are shown in supplementary Table 4.

**Surface sensing of translation (SUnSET) assay**

Cells were incubated with puromycin (10 μg/mL) at 37°C 30min. Then, western blot analysis was used to detect the immunoreactivity of anti-Puromycin (Sigma-Aldrich, Austria) (RRID: AB_2566826 and GAPDH (RRID: AB_2107436) (as loading control) antibodies in the protein suspension.

**Polysomal profiling and RT-qPCR analysis**

Cell were incubated with 100 μg/ml cycloheximide (CHX) for 15 min, scratched after an immediate wash with cold PBS containing 100 μg/ml CHX, and then incubated with polysome cell extraction buffer (50 mM MOPS, 15 mM MgCl2, 150 mM NaCl, 100 μg/ml CHX, 0.5% Triton X-100, 1 mg/ml heparin, 200 U/ml RNase inhibitor, 2 mM PMSF, 1 mM benzamidine) on ice for 10 min. Next, the cell lysis products were centrifuged at 13,000*g* at 4°C for 10 min, and supernatants were layered on the top of a 5–50% sucrose gradient tube and centrifuged at 222,200*g* at 4°C for 2.5hours in an SW41 rotor (Beckman Coulter). Fractions were collected from the top using a BR-188 Density Gradient Fractionation System (Brandel) and measured at an absorbance of 254nm. Lysate preparation and centrifugation conditions were identical to those described in the ribosome footprinting analysis section. In total, 5 fractions (2 mL) were collected, reverse transcribed, and RT-qPCR performed. A list of GAPDH and SEPHS2 primers are included in supplemental Table 3.

**RNA-seq and data analysis**

After RNA extraction, purity was assessed using a Nanodrop by measuring OD260/280 and

OD260/230 ratios, and its integrity evaluated using a Agilent 2100/4150 bioanalyzer. A total of 1 µg total RNA per sample was used as input material for the mRNA library preparation. Library quality was assessed on the Agilent 4200 TapeStation. After passing library inspection, the library preparations were sequenced on a NovaSeq 6000 platform (CHI BIOTECH CO.,LTD), and 150 bp stand-specific paired-end reads were generated. For data analysis, raw data (raw reads) of fastq format were first processed through in-house perl scripts. At the same time, Q20, Q30 and the GC content of the clean data were calculated. All the downstream analyses were performed based on high quality clean data. An index of the reference genome was built using star v2.7.10b, and paired-end clean reads were mapped to the reference genome. Quantification of gene expression levelfeatureCounts software (v2.0.3) was used to count the read numbers mapped to each gene. Next, the number of transcripts per kilobase (TPM) was calculated, with the expected number of TPM of exon model per Million mapped reads. The read counts were adjusted using the edgeR program package through one scaling normalized factor. Differential expression analysis of two conditions was performed using the edgeR R package (3.40.2). The P values were adjusted using the Benjamini & Hochberg method. A corrected P-value of 0.05 was set as the threshold for significantly differential gene expression.

**Ribo-seq** **and data analysis**

To block translational elongation, 1% volume of 100X CHX was added to fresh cell culture medium. Then, the cells were incubated under the original conditions for 15min. Next,, cells were washed with pre-chilled PBS buffer with 1% volume of 100X CHX. In order to digest RNA other than ribosome-protected fragments (RPFs), cell or tissue lysates were treated with non-specific endoribonuclease RNase I. Monosomes were purified from the lysate by fractionation on a linear sucrose gradient. Following PAGE purification, both ends of RPFs were phosphorylated and ligated with 5’ and 3’ adapters, respectively. The RNA samples were then treated with rRNA depletion kit (Qiagen, Germany) to deplete as much rRNA contamination as possible before PAGE purification of the relatively short (25~36nt) RPFs. Then, the fragments were reverse transcribed to cDNAs and amplified by PCR. After library construction (Multiplex Small RNA Library Prep Set for Illumina (Set1), NEB), the concentration of the library was measured by a Qubit® 2.0 Fluorometer. An Agilent 2100 Bioanalyzer was used to assess the insert size of the acquired library. The cDNA library products were generated using VAHTS Small RNA Library Prep Kit for Illumina V2 (Vazyme, China). After library preparation and pooling of different samples, the samples were subjected to Illumina sequencing. RiboProfiler was used for Ribo-seq data analysis. Analyses included alignment on the genome, quality control visualization, frames with length distribution, different region features, and calculation of Ribo density and RNA coverage.

**Combination analyses of RNA-seq and Ribo-seq**

**Correlation between translation and transcription**

To analyze the correlation between translation and transcription levels of indicated genes, a Pearson correlation coefficient between translation and transcription level was calculated.

**Comparison of differences between translation and transcription**

According to the expression changes at both the transcriptional and translational level, genes were classified into five groups: Transcription (significantly different only at transcriptional level); Translation (significantly different only at translational level); Homodirection (significantly different at both levels and have the same trends); Opposite (significantly different at both levels and have the opposite trends); or Unchanged (not significantly different at both levels).

**Translational efficiency (TE) calculation**

Translational efficiency was considered the ratio of translating mRNAs to total mRNAs of a gene. The formula is shown as follows: TE = (TPM in Ribo-seq) / (TPM in RNA-seq). The TE of genes was calculated and compared between samples and groups.

**Comparison of gene expression level and translational efficiency**

To analyze the correlation between translational efficiency and the transcription level of genes, a Pearson correlation coefficient between the translational efficiency and transcription level was calculated.

**Differential TE genes (DTEGs) analysis**

The t-test was used to identify differential TE genes across sample groups. Genes with a P-value <0.05 were considered as significant DTEGs. Then, significant DTEGs were analyzed by GO and KEGG Enrichment Analysis.

**Dual-Luciferase reporter assay**

The wild-type or mutant 5′ UTR sequence of the SEPHS2 5′ UTR region was cloned before the sequence of a luciferase reporter (pmirGLO plasmid, GeneChem). METTL5 overexpression or control plasmids were co-transfected with the luciferase plasmid into 293T cells. Firefly luciferase activity was normalized to Renilla luciferase measurements. The sequence of SEPHS2-5'UTR-WT or SEPHS2-5'UTR-Mutants are shown in supplementary Table 5.

**H&E staining**

Mouse organ tissue samples were used to perform H&E (H&E Staining Kit, Solarbio, China) staining. Briefly, tissue sections were deparaffinized with xylene, rehydrated through an ethanol gradient, and subjected to hematoxylin staining for 30 seconds and eosin staining for 5 seconds. The morphological structure of tissue samples were observed at a magnification of 400X to determine any toxicity of treatments on key organ systems.

**Determining the Dosage of Salvianolic Acid C**

In vitro, we selected a drug concentration of 1.5×IC50 for MM cells and validated this in bone marrow stromal cells (HS-5). Results showed that 40 μM SAC exhibited no cytotoxicity toward HS-5 cells, so we established 40 μM as the in vitro treatment concentration. In vivo, we administered tail vein injections to NSG mice at doses of 20, 40, 60, and 80 mg/kg under non-tumor-bearing conditions. Over the three-month administration period, mortality was observed in the 60 and 80 mg/kg groups, while the 20 and 40 mg/kg groups showed no mortality or adverse effects (e.g. reduced activity and lethargy, fluffy and untidy fur coats, unresponsiveness to external stimuli, and an inability to quickly get up when laid on its side, etc.). Based on these findings, we selected 40 mg/kg as the maximum therapeutic concentration.

**Supplementary Figures**

**Figure S1**


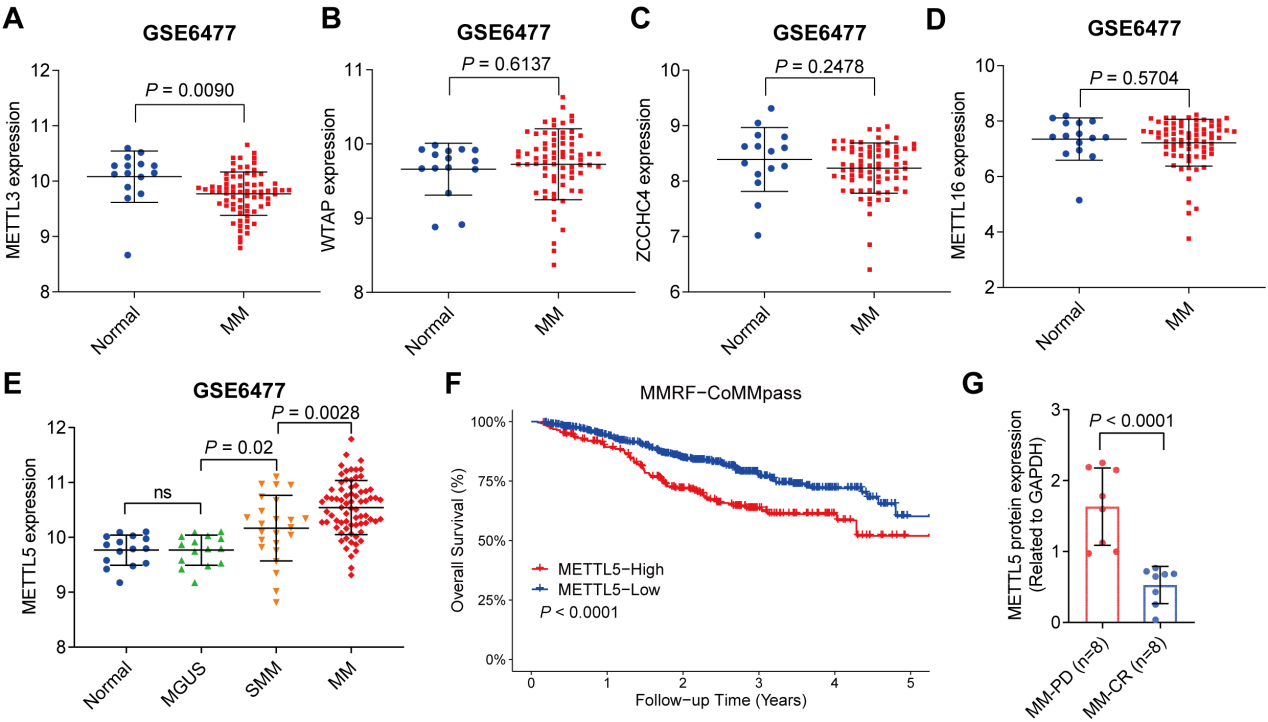


Figure S1 METTL5 overexpression is associated with a poor prognosis in MM patients. (A-D) METTL3, WTAP, ZCCHC4 and METTL16 expression in the GSE6477 MM cohort (n=15 in normal donor (ND) and n=73 in MM groups). (E) The expression of METTL5 in ND, MGUS, SMM, and MM patient BMs in the GSE6477 cohort (n=15 in ND, n=22 in MGUS, n=24 in SMM, and n=73 in MM groups). (F) Kaplan-Meier analyses of overall survival in [The Multiple Myeloma Research Foundation](http://gdc.cancer.gov/about-gdc/contributed-genomic-data-cancer-research/foundation-medicine/multiple-myeloma-research-foundation-mmrf) (MMRF) Relating Clinical Outcomes in MM to Personal Assessment of Genetic Profile (CoMMpass) cohort (n=255 in METTL5-High and n=589 in METTL5-Low groups). (G) METTL5 protein expression between MM-CR and MM-PD BM samples in our cohort (n=8 in MM-PD and n=8 in MM-CR).

**Figure S2**


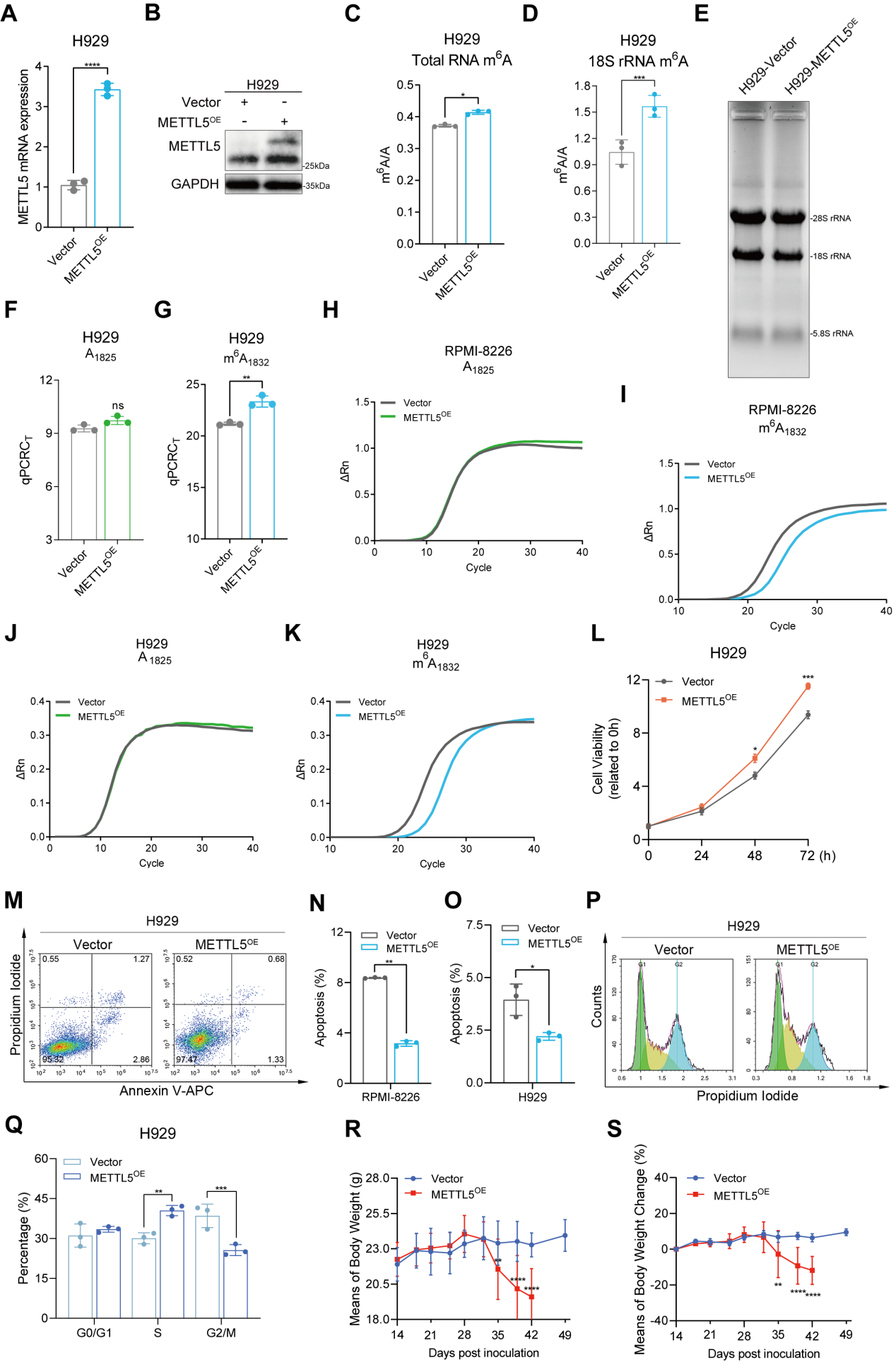


Figure S2 Overexpression of METTL5 promotes MM tumorigenesis and tumor progression in vitro and in vivo. (A) The mRNA expression of *METTL5* in vector and METTL5-overxpressing H929 cells. n=3. (B) METTL5 protein expression in H929 cells with or without METTL5 overexpression. (C-D) The m^6^A modificaion of total RNA (C) and 18S rRNA (D) in vector and METTL5-overxpressing H929 cells were determined by LC-MS/MS. (E) RNA formaldehyde denaturing gel electrophoresis of RNAs from vector and METTL5-overxpressing H929 cells. (F-G) Statistical quantification of the expression levels of A_1825_ (F) and m^6^A_1832_ (G) in 18S rRNA in H929 cells with or without METTL5 overexpression. n=3. (H-I) Representative amplified curve of A_1825_ (H) and m^6^A_1832_ (I) in RPMI-8226 cells with or without METTL5 overexpression were detected by SELECT assay. (J-K) Representative Amplified curve of A_1825_ (J) and m^6^A_1832_ (K) in H929 cells with or without METTL5 overexpression were detected by SELECT assay. (L) Growth curves of vector and METTL5-overexpression H929 cells as evaluated by CCK-8 assay. n=5. (M) Apoptosis analysis by flow cytometry of vector and METTL5-overexpressing H929 cells. (N-O) Statistical quantification for apoptosis analysis of vector and METTL5-overexperssing RMPI-8226 (N) and H929 (O) cells. n=3. (P-Q) Cell cycle analysis (P) and its statistical quantification (Q) of vector and METTL5-overexperssing H929 cells. n=3. (R-S) body weight trends (R) and average body weight change trends (S) for vector and METTL5^OE^ mice.

**Figure S3**


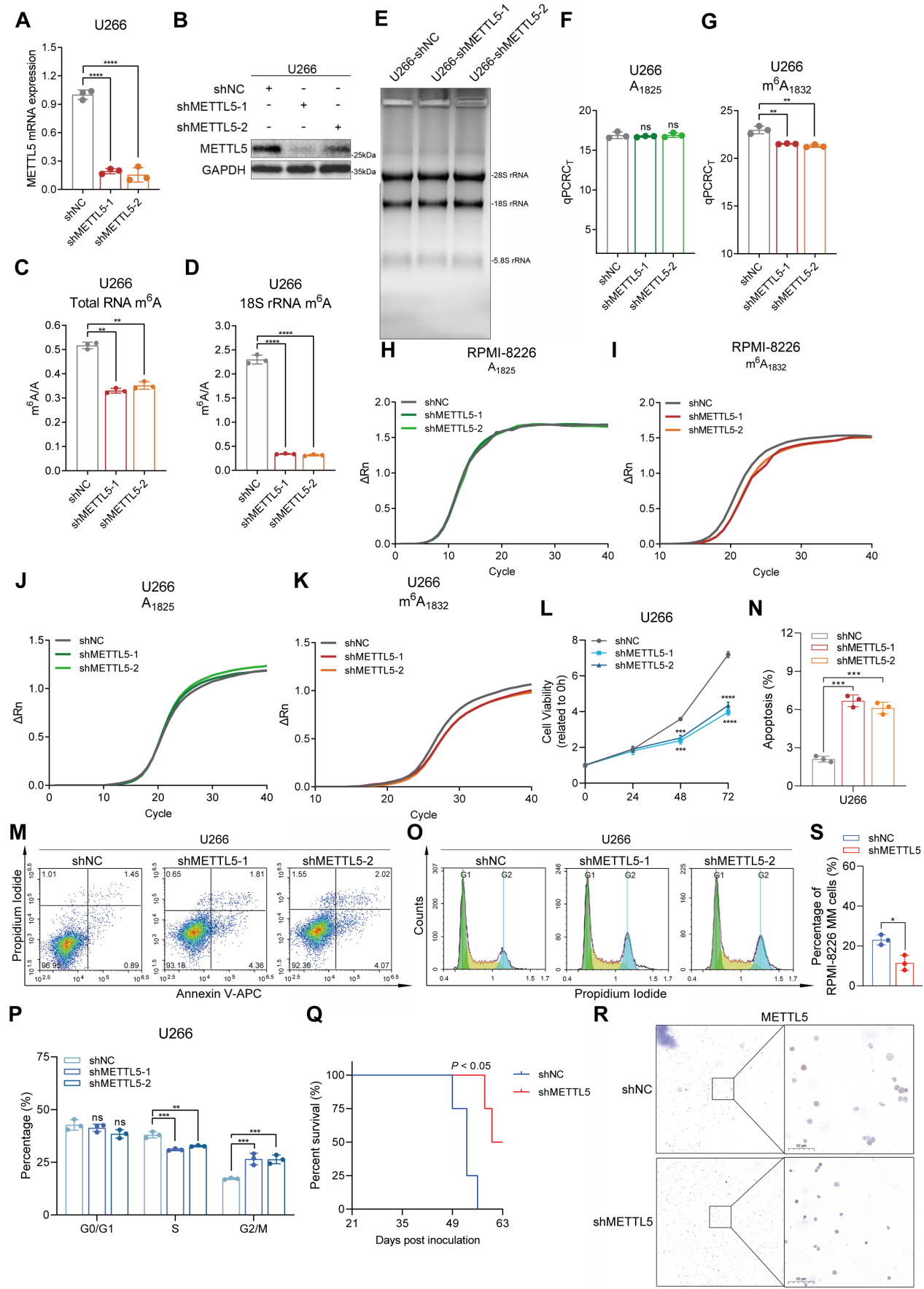


Figure S3 Knockdown of METTL5 inhibits MM progression in vitro and vivo. (A) *METTL5* mRNA expression in U266 cells with or without METTL5 knockdown. n=3. (B) The protein expression of METTL5 expression in control and METTL5-knockdown U266 cells. (C-D) The m^6^A modificaion of total RNA (C) and 18S rRNA (D) in control and METTL5-knockdown U266 cells were determined by LC-MS/MS. (E) RNA formaldehyde denaturing gel electrophoresis of RNAs from control and METTL5-knockdown U266 cells. (F-G) Statistical quantification of the expression levels of A_1825_ (F) and m^6^A_1832_ (G) in 18S rRNA in control and METTL5-knockdown U266 cells. n=3. (H-K) Representative amplified curve of A_1825_ (H and J) and m^6^A_1832_ (I and K) in control and METTL5-knockdown RPMI-8226 (H-I) and U266 (J-K) cells were detected by SELECT assay. (L) Growth curves of control and METTL5-knockdown U266 cells as evaluated by CCK-8 assay. n=5. (M-N) Apoptosis analysis (M) and its statistical quantification (N) of control and METTL5-knockdown U266 cells. n=3. (O-P) Cell cycle analysis (O) and its statistical quantification (P) of control and METTL5-knockdown U266 cells. n=3. (Q) Survival curves for shNC and shMETTL5 orthotopical xenograft models. (R-S) Representative IHC staining images of METTL5 (R) and quantification of the RPMI-8226 MM cells (S) from control and shMETTL5 mice bone marrow samples. Scale bars: left panels, 200 μm; right panels, 50 μm.

**Figure S4**


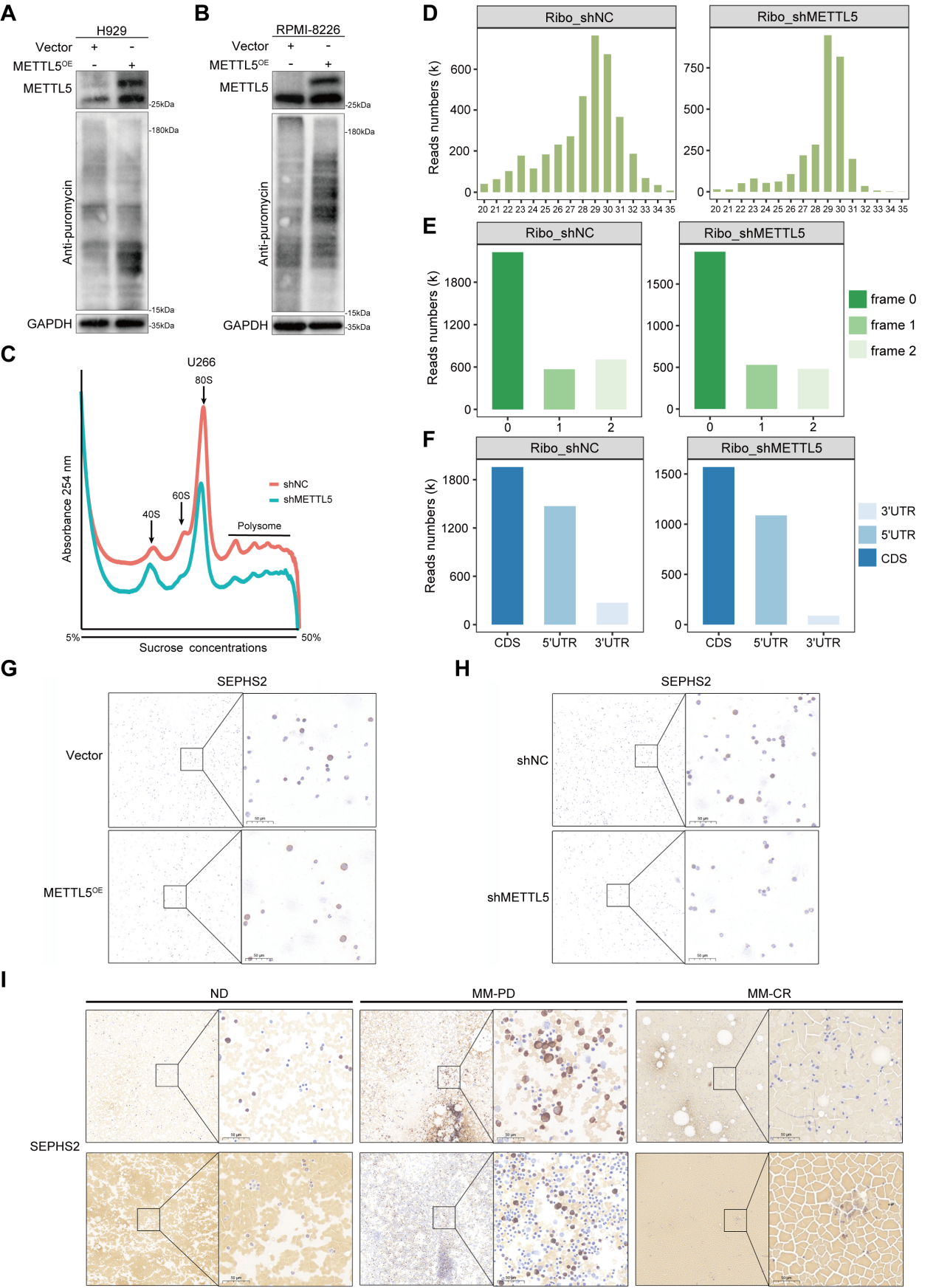


Figure S4 Knockdown of METTL5 diminishes protein synthesis in MM cells. (A-B) Global protein translation efficiency of vector and METTL5-overexpressing RPMI-8226 (A) and H929 (B) cells was detected by SUnSET assay. (C) Global translation activity of control and METTL5-knockdown U266 cells was analyzed by polysome profiling. (D-F) Codon periodicity and length distribution of ribosome-protected mRNA fragments was examined using Ribo-seq. n=3. Histogram of genome mapped reads at different lengths (D) and frames (E) with length distribution in RPMI-8226 cells with or without METTL5 knockdown. (F) Fractions of reads mapping to each reading frame in the coding sequence (CDS), the 5′-untranslated region (UTR) and the 3′-UTR in control and METTL5-knockdown RPMI-8226 cells. (G-H) Representative IHC staining images of SEPHS2 from vector and METTL5^OE^ (G) or control and METTL5-knockdown (H) mouse BM samples. Scale bars: left panels, 200 μm; right panels, 50 μm. (I) Representative IHC staining images of SEPHS2 from ND (left), MM-PD (mid) and MM-CR (right) BM samples. Scale bars: left panels, 200 μm; right panels, 50 μm.

**Figure S5**


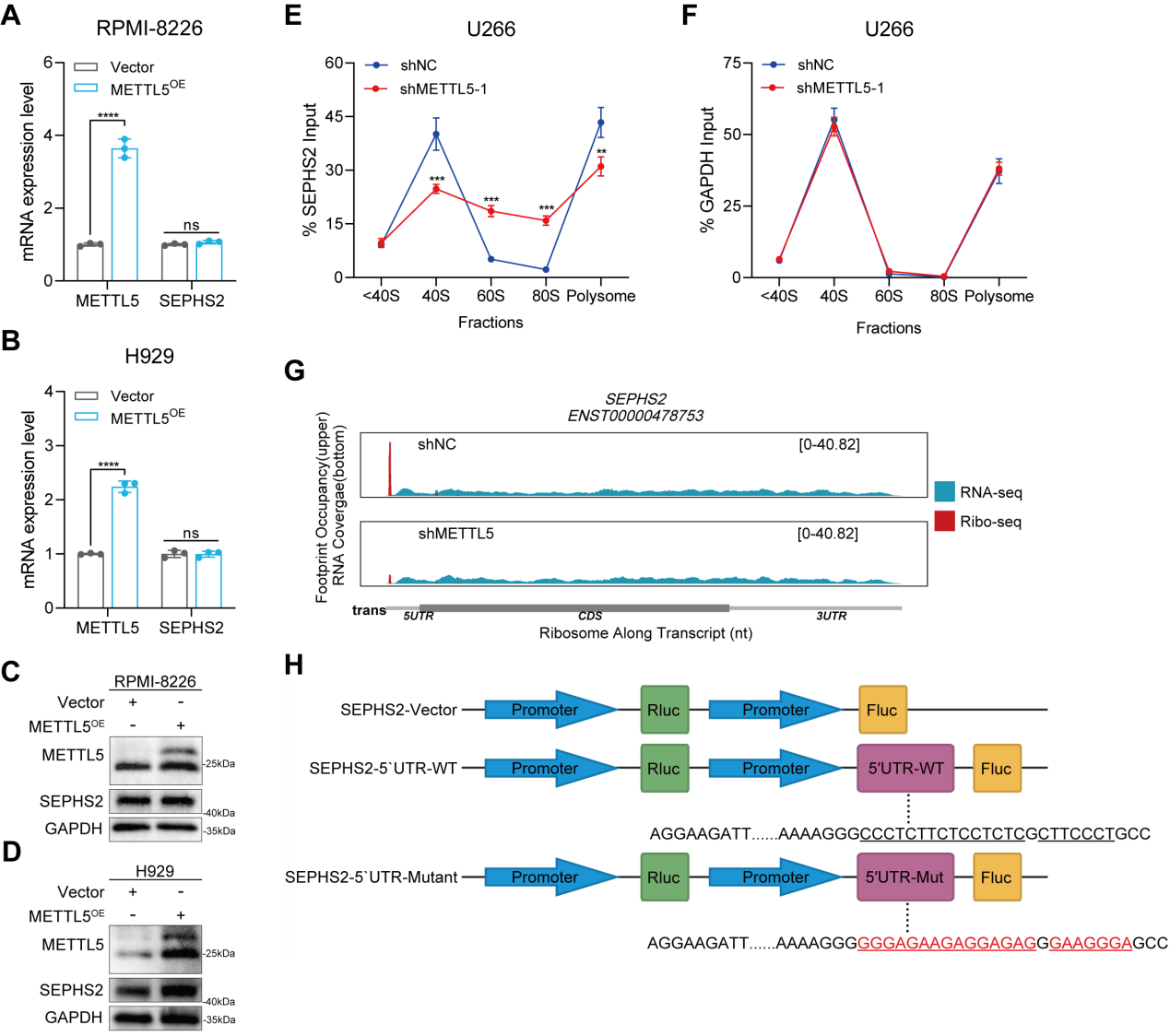


Figure S5 The 5′ TOP motif is a crucial structure enabling METTL5 to regulate the 40S ribosome's recognition of SEPHS2 and to promote its translation initiation. (A-B) METTL5 and SEPHS2 mRNA expression in vector and METTL5-overexpressing RPMI-8226 (A) and H929 (B) cells. n=3. (C-D) METTL5 and SEPHS2 protein expression in RPMI-8226 (C) and H929 (D) cells with or without METTL5 overexpression. (E-F) SEPHS2 (E) and GAPDH (F) mRNA expression levels in each fraction of polysome profiling were determined by RT-qPCR in U266 cells with or without METTL5 depletion. n=3. (G) Distribution of reads on SEPHS2 mRNA in RPMI-8226 cells with or without METTL5 knockdown was examined using Ribo-seq (red) and RNA-seq (blue). (H) Schematic of SEPHS2 luciferase reporter plasmids: SEPHS2-5′ UTR-WT (wild-type 5′ UTR of SEPHS2); SEPHS2-5′ UTR-Mutant (5′ UTR Mutant sequences were obtained by replacing the pyrimidines in wild-type 5′ UTR TOP motifs of SEPHS2 with purines).

**Figure S6**


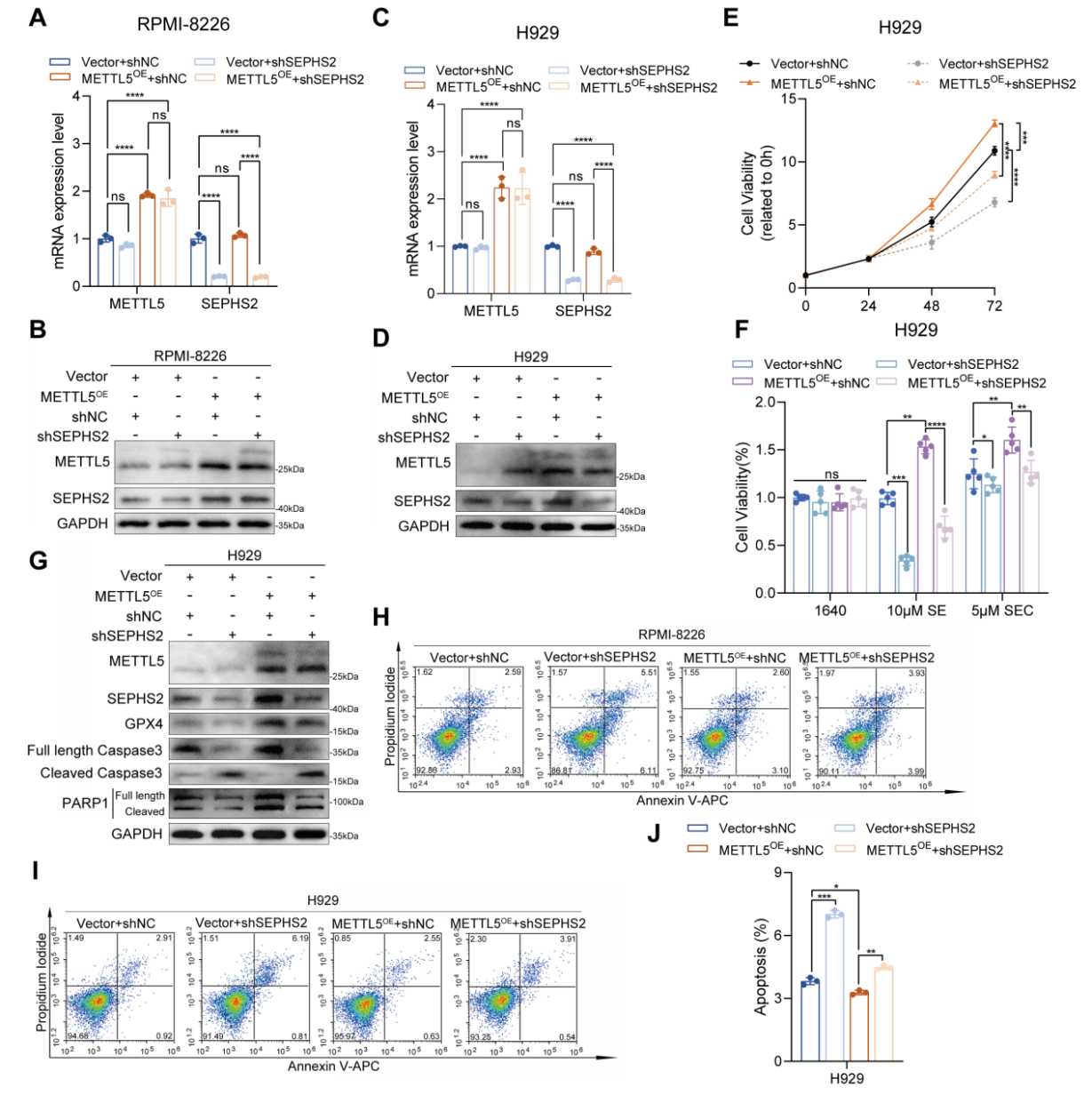


Figure S6 Silencing of SEPHS2 partially impedes the promoting role of METTL5 overexpression on the progression of MM. (A) *METTL5* and *SEPHS2* mRNA expression in vector and METTL5-overexpressing RPMI-8226 cells with or without SEPHS2 knockdown. n=3. (B) METTL5 and SEPHS2 protein expression in vector and METTL5-overexpressing RPMI-8226 cells with or without SEPHS2 knockdown. (C) *METTL5* and *SEPHS2* mRNA expression in vector and METTL5-overexpressing H929 cells with or without SEPHS2 knockdown. n=3. (D) METTL5 and SEPHS2 protein expression in vector and METTL5-overexpressing H929 cells with or without SEPHS2 knockdown. (E) Growth curves of vector and METTL5-overexpressed H929 cells, with or without SEPHS2 knockdown as evaluated by CCK-8 assay. n=5. (F) Cell viability of vector and METTL5-overexpressed RPMI-8226 (D) and H929 (E) cells, with or without SEPHS2 knockdown following treatment with DMSO, 10nM SE or 5μM SEC for 48h as evaluated by CCK-8 assay. n=5. (G) The protein expression of METTL5, SEPHS2, GPX4, γ-H2A.X (Ser139) , caspase3 and PARP1 in vector and METTL5-overexpressed H929 cells, with or without SEPHS2 knockdown was analyzed by Western blot. (H-I) Flow cytometry analysis of apoptosis in vector and METTL5-overexpressed RPMI-8226 (H) and H929 (I) cells, with or without SEPHS2 knockdown. (J) Statistical quantification of apoptosis in vector and METTL5-overexpressed H929 cells, with or without SEPHS2 knockdown. n=3.

**Figure S7**


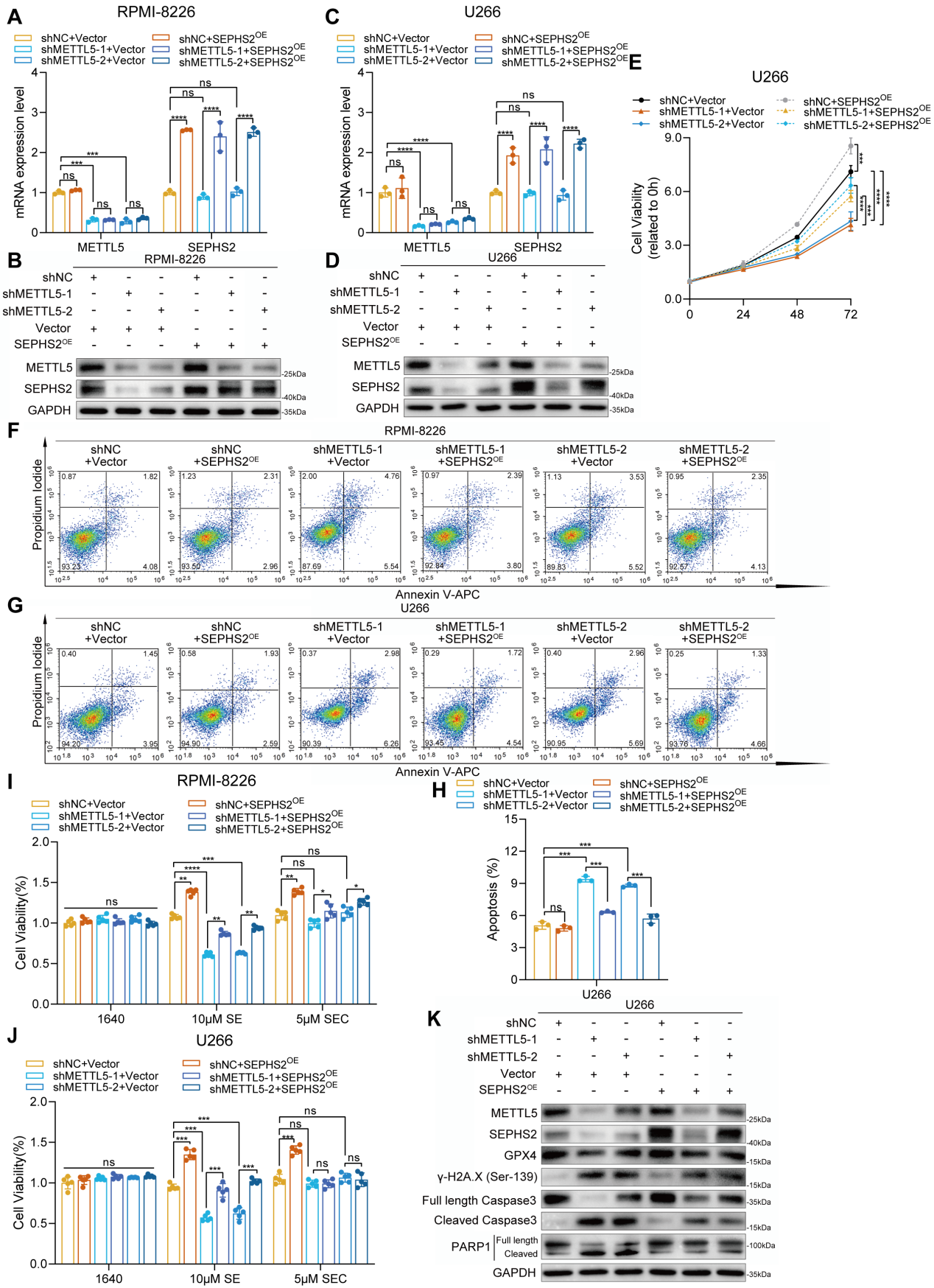


Figure S7 Overexpression of SEPHS2 promotes the malignant progression of MM cells with METTL5 knockdown in vitro. (A) *METTL5* and *SEPHS2* mRNA expression in control and METTL5-depleted RPMI-8226 cells with or without SEPHS2 restoration. n=3. (B) METTL5 and SEPHS2 protein expression in control and METTL5-depleted RPMI-8226 cells, with or without SEPHS2 restoration. (C) *METTL5* and *SEPHS2* mRNA expression in control and METTL5-depleted U266 cells, with or without SEPHS2 reexpression. n=3. (D) METTL5 and SEPHS2 protein expression in control and METTL5-depleted U266 cells, with or without SEPHS2 reexpression. (E) Growth curves of control and METTL5-depleted U266 cells, with or without SEPHS2 reexpression as evaluated by CCK-8 assay. n=5. (F-G) Flow cytometry analysis of apoptosis in control and METTL5-depleted RPMI-8226 (F) and U266 (G) cells, with or without SEPHS2 reexpression. (H) Statistical quantification of apoptosis in control and METTL5-depleted U266 cells, with or without SEPHS2 reexpression. n=3. (I-J) Cell viability of control and METTL5-depleted RPMI-8226 (I) and U266 (J) cells, with or without SEPHS2 reexpression following treatment with DMSO, 10nM SE or 5μM SEC for 48h as evaluated by CCK-8 assay. n=5. (K) The protein expression of METTL5, SEPHS2, GPX4, γ-H2A.X (Ser139) , caspase3 and PARP1 in control and METTL5-depleted U266 cells, with or without SEPHS2 reexpression was analyzed by Western blot.

**Figure S8**


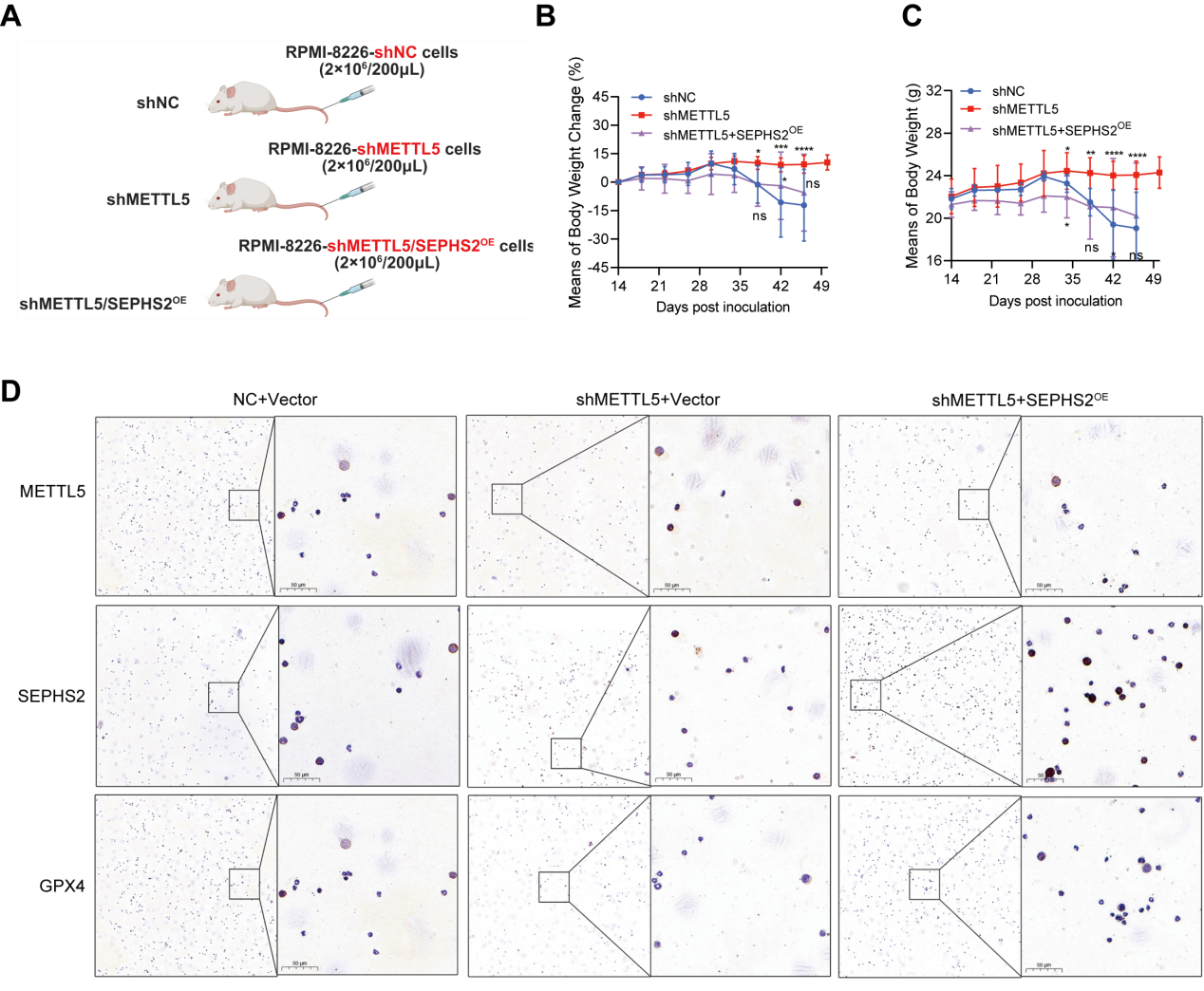


Figure S8 Overexpression of SEPHS2 promotes the malignant progression of MM cells with METTL5 knockdown in vivo. (A) Schematic diagram of orthotopical xenograft model was established through the injection of shNC/vector, shMETTL5/vector and shMETTL5/SEPHS2^OE^ cells into the tail vein of NSG mice (n=5 in each group) (Created with BioRender.com.). (B-C) Body weight trends (B) and average body weight change trends (C) for each group of orthotopical xenograft models. (D) Representative IHC staining images of METTL5 from shNC/vector, shMETTL5/vector and shMETTL5/SEPHS2^OE^ mice bone marrow samples. Scale bars: left panels, 200 μm; right panels, 50 μm.

**Figure S9**


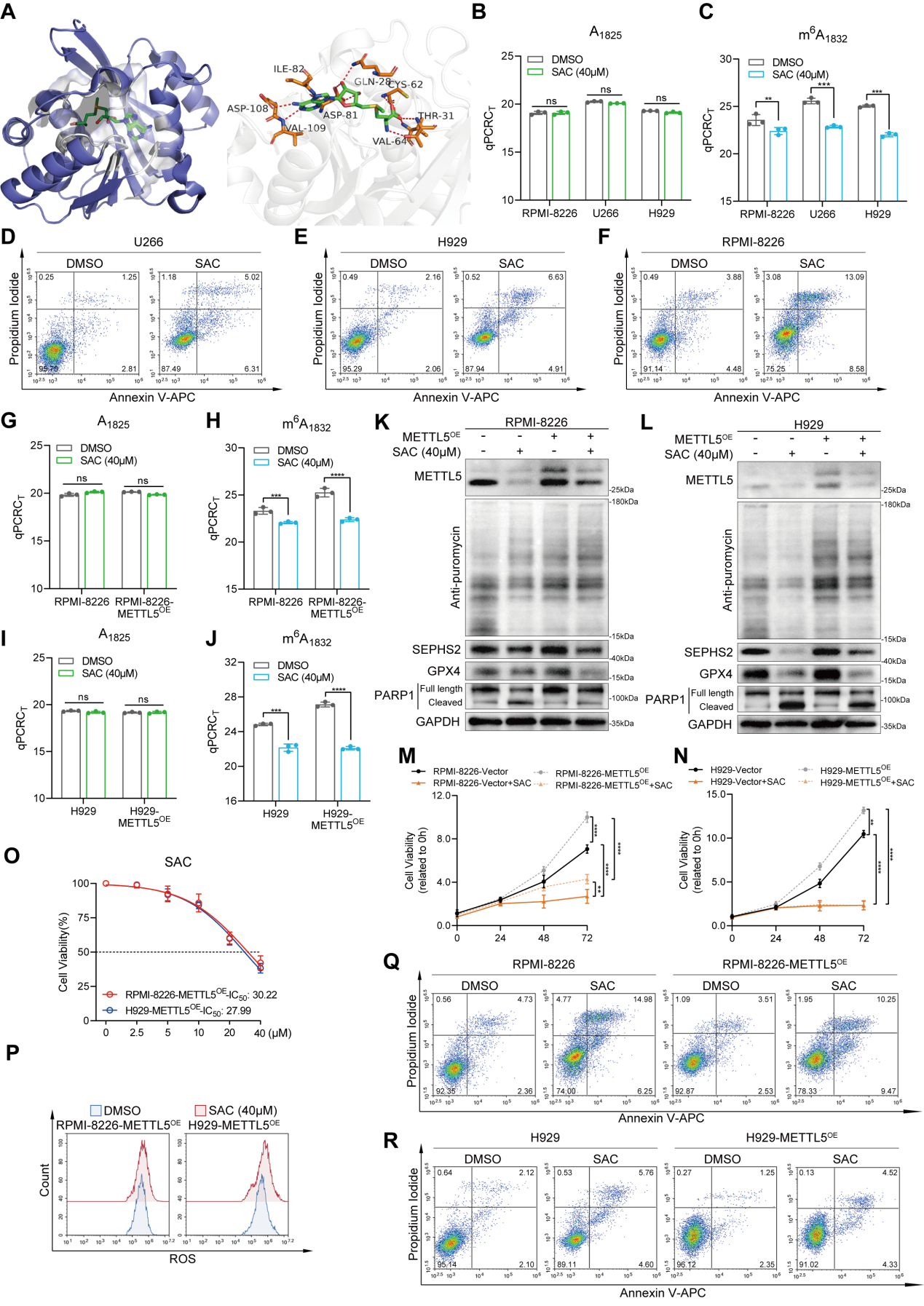


Figure S9 SAC induces apoptosis of MM cells by targeting METTL5 in vitro. (A) Crystal structure of METTL5 (6H2U) and interactions between its ligand and the active pocket. (B-C) Statistical quantification of the expression levels of A_1825_ (B) and m^6^A_1832_ (C) sites in 18S rRNA determined by SELECT in U266, RPMI-8226, and H929 cells treated with either DMSO or SAC for 48h. (D-F) Flow cytometry analysis of apoptosis in U266 (D), RPMI-8226 (E) and H929 (F) cells treated with either DMSO or SAC for 48h. (G-J) Statistical quantification of the expression levels of A_1825_ (G and I) and m^6^A_1832_ (H and J) sites in 18S rRNA in RPMI-8226 (G-H) and H929 (I-J) cells with or without METTL5 overexpression following treatment with either DMSO or SAC for 48h. (K-L) Western blot analysis of METTL5, SEPHS2, GPX4, and PARP1 protein levels, and global protein translation efficiency in RPMI-8226 (K) and H929 (L) cells with or without METTL5 overexpression following treatment with either DMSO or SAC for 48h. (M-N) apoptosis (M-N) in RPMI-8226 (M) and H929 (N) cells with or without METTL5 overexpression following treatment with either DMSO or SAC for 48h. (O) IC_50_ values of SAC in RPMI-8226-METTL5^OE^ and H929-METTL5^OE^ cells determined by CCK-8 assay. (P-R) Flow cytometry analysis of both ROS levels (P) and apoptosis (Q-R) in RPMI-8226 (Q) and H929 (R) cells with or without METTL5 overexpression following treatment with either DMSO or SAC for 48h.

**Figure S10**


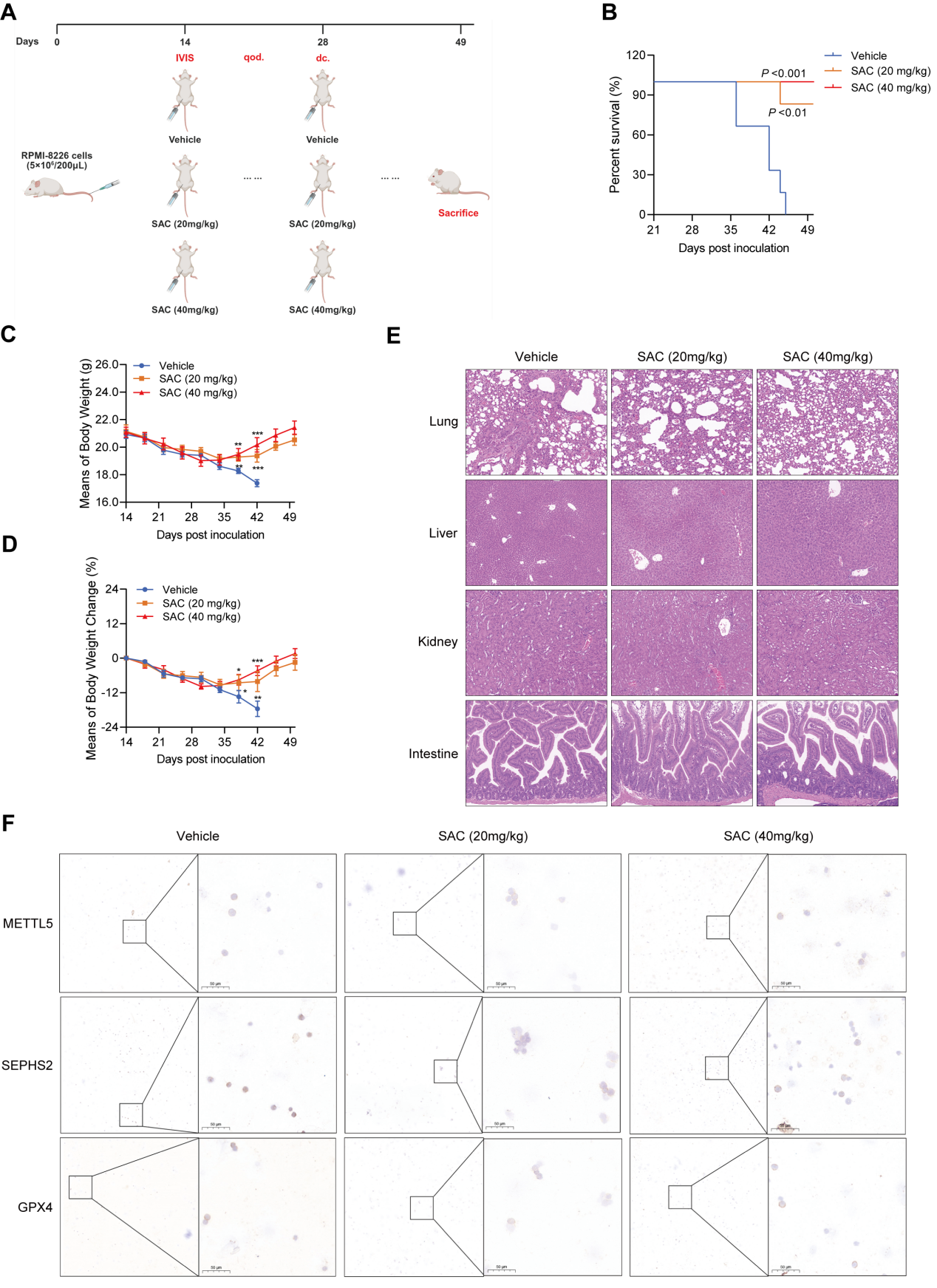


Figure S10 SAC is a potent and safe METTL5 inhibitor that can mitigate MM burden and enhance the survival rate of MM mouse models. (A) An orthotopical xenograft model was established through the injection of RPMI-8226-Luc cells into the tail vein of NSG mice. After MM lesions were detected via whole-body bioluminescence image (2 weeks post MM cell injection), mice were allocated to receive either SAC (20 or 40 mg/kg, intraperitoneal injection, 5 days a week, until all mice in a group had died) or vehicle control treatment (Created with BioRender.com.). (B-D) Survival curves (B) , body weight trends (C) and average body weight change trends (D) for orthotopical xenograft models subjected to either vehicle control or SAC treatment. (E) Representative IHC staining images demonstrating METTL5, SEPHS2, and GPX4 in BM samples from both vehicle control and SAC treated mice. Scale bars: left panels, 200 μm; right panels, 50 μm. (F) Representative images showcasing H&E staining of lung, liver, kidney and intestine samples from both vehicle control and SAC treated mice.

**Supplementary Tables**

**Supplementary Table S1. Clinical characteristics of normal donors and MM patients**

| **Characteristics** | **Median (range)** | **NDs** | **MM-PDs** | **MM-CRs** |
| --- | --- | --- | --- | --- |
| Sex |  |  |  |  |
| Female |  | 7 | 7 | 6 |
| Male |  | 8 | 8 | 12 |
| Total |  | 15 | 15 | 18 |
| Median age, y | 64.14 (46-71) |  |  |  |
| Younger than 50 y |  | 0 | 2 | 0 |
| 50–60 y |  | 6 | 4 | 5 |
| Older than 60 y |  | 9 | 10 | 12 |
| Total |  | 15 | 16 | 17 |

**Supplementary Table S2. Chemical reagents used in this study.**

| **Chemical reagents** | **Source** | **Identifier** |
| --- | --- | --- |
| Selenocysteine | TargetMol | Cat# T9427 |
| Sodium Selenite | Sigma-Aldrich | Cat# 10102-18-8 |
| Salvianolic Acid C | TargetMol | Cat# T3149 |
| RPMI Medium 1640 Basic (1X) | Gibco | Cat# C11875500BT |
| Fetal Bovine Serum | Gibco | Cat# 10099-141 |
| Puromycin | Beyotime | Cat# ST551 |
| RIPA | Solarbio | Cat# R0010 |
| RNAiso Plus | Takara | Cat# 9109 |
| Protease and PhosphataseInhibitor Cocktail | NCM Biotech | Cat# P002 |
| Cycloheximide | Sigma-Aldrich | Cat# C7698 |
| 10X MOPS Running Buffer | Sangon Biotech | Cat# C516042 |
| Methanol | Xilong Scientific | Cat# A110202938 |
| Water-free Ethanol | Xilong Scientific | Cat# 1030001-01-01 |
| DEPC Water | Sangon Biotech | Cat# B501005 |
| Isopropanol | Xilong Scientific | Cat# E-150191 |
| Trichloromethane | Servicebio | Cat# G3014-02 |
| Adenosine 5'-Triphosphate (ATP) | NEB | Cat# P0756S |
| rCutSmart™ Buffer | NEB | Cat# B6004S |
| SplintR® Ligase | NEB | Cat# M375S |
| Phosphate Buffered Saline | Servicebio | Cat# G4250-500ML |
| Tris-Glycine SDS-PAGE Running Buffer (Powder) | Servicebio | Cat# G2018-1L |
| Tris-Glycine Transfer Buffer (Powder) | Servicebio | Cat# G2017-1L |
| Tris Buffered Saline (TBS，Powder） | Servicebio | Cat# G0001-2L |
| NON-FatPowderedMilk | Solarbio | Cat# D8340 |
| NCM SDS-PAGE Loading Buffer，5X | NCM Biotech | Cat# WB2001 |
| CELLSAVING | NCM Biotech | Cat# C40100 |
| Tween-20 | Solarbio | Cat# T8220 |
| Antibody eluent (enhanced) | Servicebio | Cat# G2079-100ML |
| Deoxynucleotide (dNTP) | NEB | Cat# N0447S |
| Bst 2.0 DNA Polymerase | NEB | Cat# M537S |
| 10X RNA Glycerol Gel Loading Buffer | Sangon Biotech | Cat# B548318 |
| Formamide | Macklin | Cat# F809511 |
| N6-methyladenosine (m6A) | Selleck | Cat# S3190 |
| Adenosine | Selleck | Cat# S1647 |
| Thymidine | Selleck | Cat# S4803 |
| Transfer Membrane | Millipore | Cat# ISEQ00010 |
| Alkaline Phosphatase (Shrimp) | Takara | Cat# 2660A |
| S1 Nuclease | Takara | Cat# 2410A |
| DMSO | Solarbio | Cat# D8371 |
| 37% Formaldehyde | Sigma-Aldrich | Cat# F8775 |
| Propidium Iodide Solution | Biolegend | Cat# 421301 |
| FOXP3 Fix/Perm Buffer Set | Biolegend | Cat# 421403 |
| ZymocleanTMGel RNA Recovery Kit | Zymo Research | Cat# R1011 |
| PAGE Gel Fast Preparation Kit (10%) | EpiZyme | Cat# PG112 |
| PAGE Gel Fast Preparation Kit (12.5%) | EpiZyme | Cat# PG113 |
| APC Annexin V Apoptosis Detection Kit with PI | Biolegend | Cat# 640932 |
| Cell Counting Kit-8 | APExBIO | Cat# K1018 |
| PierceTM BCA Protein Assay Kit | ThermoFisher | Cat# 23227 |
| Chemistar High-sig ECL Western blotting substrate | NCM Biotech | Cat# P10200 |
| PrimeScript™ RT reagent Kit with gDNA Eraser (Perfect Real Time) | Takara | Cat# RR047A |
| TB Green® Premix Ex Taq™ II (Tli RNaseH Plus) | Takara | Cat# RR820A |
| Human lambda immunoglobulin light chain,λ-IgLC ELISA Kit | Jianglaibio | Cat# JL12360 |
| CD138 MicroBeads, human | Miltenyi Biotec | Cat# 130-051-301 |
| MACS BSA Stock Solution | Miltenyi Biotec | Cat# 130-091-376-1 |
| autoMACS Rinsing Solution | Miltenyi Biotec | Cat# 130-091-222-1 |
| LS Columns | Miltenyi Biotec | Cat# 130-042-401 |
| MiniMACS Separation Unit | Miltenyi Biotec | Cat# 130-042-102 |
| Red Blood Cell Lysis Buffer | Solarbio | Cat# R1010 |
| RNase A | Solarbio | Cat# R8020 |
| Ethidium bromide | Solarbio | Cat# E1020 |
| Formic acid | Xilong Scientific | Cat# 12700602 |
| D-Luciferin, Potassium Salt | Maokang Biotechnology | Cat# MX4603-1G |
| Bovine Serum Albumin | Servicebio | Cat# GC305010-5g |
| Hematoxylin staining Solution | Servicebio | Cat# G1004-100ML |
| Hematoxylin differentiation solution | Servicebio | Cat# G1039-100ML |
| Hematoxylin antiblue solution | Servicebio | Cat# G1040-100ML |
| Lymphocyte Separation Medium | Solarbio | Cat# R8610 |
| CellROX® Deep Red Reagent | Thermo Fisher | Cat# C10422 |
| Hitrans B2 | GeneChem | Cat# REVG006-II |

**Supplementary Table S3.Primers for RT- qPCR and SELECT-qPCR analysis in this study.**

| **Name** | **Sequence** | **Source** |
| --- | --- | --- |
| Primer for GAPDH: | Forward: TGCCAAATATGATGACATCAAGAA Reverse: GGAGTGGGTGTCGCTGTTG | Sangon Biotech |
| Primer for METTL5: | Forward: AGCAGGGTTGTGTGTTGGAT Reverse: GGTCCCAAAGGGAGGATTCA | Sangon Biotech |
| Primer for SEPHS2: | Forward: TGTCTGCCAAGAGAACAGGC Reverse: TGGTGACCCTCTCCGTACTT | Sangon Biotech |
| Primer for Luciferase: | Forward: CTGGGACGAAGACGAACAC  Reverse: GAAGACCTGCGACACCTG | Sangon Biotech |
| SELECT Probe for 18S rRNA A_1825_ site: | Up: TAGCCAGTACCGTAGTGCGTGCCTACGGAAACCTTGTTACGAC Down: phos/ TTTACTTCCTCTAGATAGTCAAGTTCGACCAGAGGCTGAGTCGCTGCAT | Sangon Biotech |
| SELECT Probe for 18S rRNA m^6^A_1832_ site: | Up: TAGCCAGTACCGTAGTGCGTGGGTTCACCTACGGAAACCTTG Down: phos/ TACGACTTTTACTTCCTCTAGATAGTCAAGCAGAGGCTGAGTCGCTGCAT | Sangon Biotech |
| Primer for SELECT-qPCR: | Forward: ATGCAGCGACTCAGCCTCTG Reverse: TAGCCAGTACCGTAGTGCGTG | Sangon Biotech |

**Supplementary Table S4. Antibodies used in this study.**

| **Antibodies used for Westrern-blot and immumohistochemical staining** | | | | | |
| --- | --- | --- | --- | --- | --- |
| **Antibodies** | **Source** | **Clone** | **Host** |  | **Dilution** |
| anti-METTL5 | Proteintech | Polyclonal | Rabbit | Cat# 16791-1-AP | 1: 1000 |
| anti-Puromycin | Sigma-Aldrich | Monoclonal | Mouse | Cat# MABE343 | 1: 3000 |
| anti-SEPHS2 | Proteintech | Polyclonal | Rabbit | Cat# 14109-1-AP | 1: 2000 |
| anti-GPX4 | Proteintech | Monoclonal | Mouse | Cat# 67763-1-Ig | 1: 2000 |
| anti-TXNRD1 | Proteintech | Polyclonal | Rabbit | Cat# 11117-1-AP | 1: 3000 |
| anti-GAPDH | Proteintech | Monoclonal | Mouse | Cat# 60004-1-Ig | 1: 2000 |
| Anti-PARP1 | Huabio | Monoclonal | Rabbit | Cat# ET1608-56 | 1: 2000 |
| Anti-active+pro Caspase-3 | Huabio | Monoclonal | Rabbit | Cat# ET1608-64 | 1: 2000 |
| Anti-Phospho-Histone H2A.X(S139) | Huabio | Monoclonal | Rabbit | Cat# ET1602-2 | 1: 2000 |
| HRP-conjugated Affinipure Goat Anti-Mouse IgG(H+L) | Proteintech | Polyclonal | Rabbit | Cat# SA00001-1 | 1: 7500 |
| HRP-conjugated Affinipure Goat Anti-Rabbit IgG(H+L) | Proteintech | Polyclonal | Rabbit | Cat# SA00001-2 | 1: 7500 |
| **Antibodies used for flow cytometry** | | | | | |
| **Antibodies** | **Source** | **Clone** | **Subtype** | **Identifier** | **Fluorochrome** |
| anti-mouse/human Ki-67 Antibody | Biolegend | Monoclonal | Rabbit IgG2b, κ | Cat# 151210 | PE |
| anti-human CD45 Antibody | Biolegend | Monoclonal | Mouse IgG1, κ | Cat# 304026 | PerCP |
| anti-human CD38 Antibody | Biolegend | Monoclonal | Mouse IgG1, κ | Cat# 356604 | PE |
| anti-human CD138 (Syndecan-1) Antibody | Biolegend | Monoclonal | Mouse IgG1, κ | Cat# 352308 | APC |

**Supplementary Table S5. Sequences of Target Seq and shRNAs**

| **Name** | **Sequence** | **Source** |
| --- | --- | --- |
| sh-METTL5-1 Target Seq | 5'-ATGTGATGTGTGCTTATTATC-3' | GeneChem Co., Ltd |
| sh-METTL5-1 sense | 5'-CCGGATGTGATGTGTGCTTATTATCCTCGAGGATAATAAGCACACATCACATTTTTTG-3' | GeneChem Co., Ltd |
| sh-METTL5-1 anti-sense | 5'-AATTCAAAAAATGTGATGTGTGCTTATTATCCTCGAGGATAATAAGCACACATCACAT-3' | GeneChem Co., Ltd |
| sh-METTL5-2 Target Seq | 5'-ATGCAGAAGAGTTTGAGTTAA-3' | GeneChem Co., Ltd |
| sh-METTL5-2 sense | 5'-CCGGATGCAGAAGAGTTTGAGTTAACTCGAGTTAACTCAAACTCTTCTGCATTTTTTG-3' | GeneChem Co., Ltd |
| sh-METTL5-2 anti-sense | 5'-AATTCAAAAAATGCAGAAGAGTTTGAGTTAACTCGAGTTAACTCAAACTCTTCTGCAT-3' | GeneChem Co., Ltd |
| METTL5-overexpressiom Target Seq | 5'-ATGAAGAAAGTAAGGCTTAAGGAACTAGAGAGTCGCCTGCAACAAGTGGATGGATTTGAAAAGCCCAAGCTACTTCTGGAACAGTATCCTACCAGGCCGCACATTGCAGCATGTATGCTCTATACAATCCATAACACTTATGATGACATTGAAAATAAAGTCGTTGCAGATCTAGGATGTGGTTGTGGAGTACTTAGCATCGGAACTGCAATGTTAGGAGCAGGGTTGTGTGTTGGATTTGACATAGATGAAGACGCATTGGAAATATTTAATAGGAATGCAGAAGAGTTTGAGTTAACAAATATTGACATGGTTCAATGTGATGTGTGCTTATTATCTAACAGAATGTCCAAGTCATTCGATACAGTAATTATGAATCCTCCCTTTGGGACCAAAAATAATAAAGGGACAGATATGGCTTTTCTAAAGACTGCTTTGGAAATGGCAAGAACAGCAGTATATTCCTTACACAAATCCTCAACTAGAGAACATGTTCAAAAGAAAGCTGCAGAATGGAAAATCAAGATAGATATTATAGCAGAACTTCGATATGACCTGCCAGCATCATACAAGTTTCACAAAAAGAAATCAGTGGACATTGAAGTGGACCTAATTCGGTTTTCCTTT-3' | GeneChem Co., Ltd |
| Sh-SEPHS2 Target Seq | 5'-CGAGTTATTGAAGTCCTGCCT-3' | GeneChem Co., Ltd |
| Sh-SEPHS2 sense | 5'-CCGGCGAGTTATTGAAGTCCTGCCTCTCGAGAGGCAGGACTTCAATAACTCGTTTTTG-3' | GeneChem Co., Ltd |
| Sh-SEPHS2 anti-sense | 5'-AATTCAAAAACGAGTTATTGAAGTCCTGCCTCTCGAGAGGCAGGACTTCAATAACTCG-3' | GeneChem Co., Ltd |
| SEPHS2-overexpressiom Target Seq | 5'-ATGGCGGAAGCCTCGGCGACGGGCGCCTGCGGAGAGGCGATGGCAGCGGCGGAAGGCTCCTCGGGCCCGGCGGGCTTGACTCTGGGCCGGAGCTTCTCGAACTACCGGCCCTTCGAGCCCCAGGCGTTGGGCCTCAGCCCGAGCTGGCGGCTGACGGGCTTCTCCGGCATGAAGGGCTGAGGCTGCAAGGTCCCGCAGGAGGCGCTGCTCAAACTCCTGGCGGGACTGACGCGGCCGGACGTGCGGCCCCCGCTGGGCCGGGGCCTGGTGGGTGGCCAGGAAGAGGCGTCCCAGGAAGCCGGCCTGCCGGCAGGAGCGGGCCCCAGCCCCACCTTTCCAGCCCTGGGCATCGGGATGGACTCCTGCGTCATCCCCCTGAGGCACGGGGGCCTGTCACTGGTGCAGACCACGGACTTCTTTTACCCCTTGGTAGAAGATCCCTACATGATGGGGCGCATAGCTTGTGCCAACGTGCTGAGTGACCTCTACGCCATGGGGATTACTGAGTGTGACAACATGTTGATGTTACTCAGCGTCAGCCAGAGTATGAGTGAGGAGGAACGCGAAAAGGTAACGCCACTCATGGTCAAAGGCTTTCGGGATGCGGCTGAGGAAGGAGGGACGGCAGTGACCGGTGGGCAAACGGTGGTCAACCCTTGGATTATAATCGGTGGAGTTGCCACTGTAGTATGCCAACCAAATGAGTTCATAATGCCGGACAGCGCCGTCGTTGGGGACGTGCTGGTGTTAACCAAACCGTTAGGAACCCAGGTTGCTGTCAATGCCCACCAATGGCTGGATAATCCTGAAAGATGGAATAAAGTAAAGATGGTGGTCTCCAGAGAAGAGGTGGAGCTGGCCTATCAGGAAGCCATGTTCAATATGGCTACCCTCAACAGAACTGCTGCAGGTTTAATGCACACATTTAATGCCCATGCGGCCACAGATATCACAGGCTTTGGCATTCTAGGACACTCCCAGAACCTTGCAAAACAACAAAGAAATGAAGTGTCCTTTGTTATTCATAATCTGCCAATAATTGCCAAGATGGCTGCCGTCAGCAAGGCCAGTGGACGGTTTGGGCTTCTTCAAGGAACCTCAGCTGAAACCTCTGGGGGATTACTGATTTGTCTGCCAAGAGAACAGGCGGCTCGCTTTTGTTCTGAAATCAAATCCTCCAAGTACGGAGAGGGTCACCAAGCGTGGATCGTTGGCATTGTGGAAAAGGGAAACCGAACGGCCCGGATCATTGACAAGCCGCGAGTTATTGAAGTCCTGCCTCGTGGGGCCACAGCTGCTGTTCTTGCTCCTGACAGTTCAAATGCCTCCTCTGAGCCTAGCTCGGGAGGTGGAGGATCAGACTACAAGGATGACGATGACAAGGATTACAAAGACGACGATGATAAGGACTATAAGGATGATGACGACAAATGAGATGAAAGAACAGAAGTTGTTTGGACCTTAGAGCCATTGTCCACAATCACGGATGGTTCTCAAGAGTTGATTGTAAGAAATTTCCAAAGAAGGCTGCCTGCATAGTGGTTCCGGCTGCCCTTTCTAGGTGATTGGAATCAGCCCATCTAAAGCAGTCTTTATATGCATTCCGAGGCCAGAGTAACATTTTGAACTTTGGGGGGATATTTGTTCATCACTTGGGTAGAAGAGGAGCAAAAATACCTCTGTTTTCTCTTGCCAAAGTAAGATGAAGCTATTCCAGGTTGAGGGATTTTTCTTTGCACGGGGTTGATTAATTTCTGCACAGGGAGTGAGATTATTAAAGTAACACACACACAAAGTAAATTGCAAAATGAAAAAAATTAGAAGCAAATGAGTTTTGGACCAATATTGTTGATAAATCTAAATTGTTAAGAGAGATCTTATAATGCAACATCAAATTCTTTATTCAATTTTACTGAAGTACTGGCTCTTTCCTGCTCTGGACAAGAATTGAGCAACTTGTCTGATGACTGGGAAAGGAGGACCTGCAACCATCTGACTTGGTCTCTGTTAATGACGTCTCTCCCTCTAAACCCCATTAAGGACTGGGAGAGGCAGAGCAAGCCTCAGAGCCCAGGCCTCAGTGGTCATTAAGATGTTAAGTCTTTTGCGGCAGATTCCTGGTGATTTGATCAATAAAGAGTAATTTCTTGCTAAATAAATAAAAGAAACCTTGTTGAAAAACTA-3' | GeneChem Co., Ltd |
| SEPHS2-5'UTR-WT Target Seq | 5'-AGGAAGATTGTGCATGCCGTGGGTCTGACGGCTTGAGTAGCGCTAGGGAGAATCCCTGCAGGTAATATTTGACTTTTGCTTCATATTAATCTGAGTGGAAAATAAAAGGGCCCTCTTCTCCTCTCGCTTCCCTGCCGGGCAGGCGCC-3' | GeneChem Co., Ltd |
| SEPHS2-5'UTR-Mutant Target Seq | 5'-AGGAAGATTGTGCATGCCGTGGGTCTGACGGCTTGAGTAGCGCTAGGGAGAATCCCTGCAGGTAATATTTGACTTTTGCTTCATATTAATCTGAGTGGAAAATAAAAGGGGGGAGAAGAGGAGAGGGAAGGGAGCCGGGCAGGCGCC-3' | GeneChem Co., Ltd |

**Supplementary Table S6. Differential translation efficiency genes.**

| **SYMBOL** | **logFC** | **PValue** | **Con_1** | **Con_2** | **Con_3** | **KD_1** | **KD_2** | **KD_3** |
| --- | --- | --- | --- | --- | --- | --- | --- | --- |
| SEPHS2 | -2.060 | 0.000 | 2.473 | 2.713 | 2.358 | 0.557 | 0.590 | 0.663 |
| TAF3 | -1.983 | 0.000 | 1.115 | 1.194 | 1.029 | 0.307 | 0.244 | 0.294 |
| NFE2L2 | -1.575 | 0.000 | 0.842 | 0.921 | 0.815 | 0.275 | 0.275 | 0.315 |
| INSIG1 | -1.567 | 0.000 | 1.161 | 1.247 | 1.179 | 0.414 | 0.420 | 0.377 |
| DTWD1 | -1.544 | 0.000 | 4.044 | 4.339 | 4.273 | 1.500 | 1.518 | 1.321 |
| SELENOT | -0.894 | 0.000 | 2.233 | 2.174 | 2.211 | 1.227 | 1.132 | 1.202 |
| HSBP1 | -0.685 | 0.000 | 1.473 | 1.575 | 1.538 | 0.965 | 0.932 | 0.956 |
| NUDT16 | -0.660 | 0.000 | 0.349 | 0.369 | 0.352 | 0.220 | 0.232 | 0.225 |
| CMSS1 | 0.834 | 0.000 | 1.025 | 1.023 | 1.053 | 1.782 | 1.870 | 1.876 |
| NLN | 0.850 | 0.000 | 0.227 | 0.264 | 0.258 | 0.444 | 0.448 | 0.459 |
| SNRNP25 | 0.962 | 0.000 | 0.927 | 0.919 | 0.830 | 1.786 | 1.677 | 1.753 |
| CADM1 | 1.004 | 0.000 | 0.815 | 0.832 | 0.867 | 1.658 | 1.671 | 1.714 |
| TPD52L2 | 1.022 | 0.000 | 0.418 | 0.394 | 0.398 | 0.783 | 0.855 | 0.819 |
| C12orf65 | 1.091 | 0.000 | 0.837 | 0.707 | 0.826 | 1.693 | 1.686 | 1.668 |
| COPS2 | 1.135 | 0.000 | 0.418 | 0.395 | 0.394 | 0.920 | 0.827 | 0.905 |
| CHCHD7 | 1.146 | 0.000 | 0.851 | 0.998 | 0.783 | 1.957 | 1.918 | 1.949 |
| TMEM59 | 1.220 | 0.000 | 0.621 | 0.710 | 0.713 | 1.638 | 1.528 | 1.595 |
| METTL7A | 1.226 | 0.000 | 0.365 | 0.349 | 0.354 | 0.824 | 0.799 | 0.875 |
| SERINC1 | 1.231 | 0.000 | 0.585 | 0.470 | 0.563 | 1.288 | 1.290 | 1.221 |
| PPP6C | 1.292 | 0.000 | 0.388 | 0.455 | 0.420 | 1.056 | 0.979 | 1.058 |
| SPPL2A | 1.318 | 0.000 | 0.575 | 0.635 | 0.660 | 1.610 | 1.446 | 1.605 |
| RABEPK | 1.322 | 0.000 | 0.346 | 0.343 | 0.280 | 0.796 | 0.834 | 0.792 |
| TMEM14B | 1.395 | 0.000 | 0.133 | 0.105 | 0.130 | 0.330 | 0.315 | 0.323 |
| MFGE8 | 1.398 | 0.000 | 0.421 | 0.600 | 0.503 | 1.333 | 1.346 | 1.339 |
| ARHGAP42 | 1.409 | 0.000 | 0.259 | 0.257 | 0.201 | 0.648 | 0.619 | 0.638 |
| AQP3 | 1.420 | 0.000 | 0.870 | 0.802 | 0.794 | 2.140 | 2.232 | 2.227 |
| NIPSNAP3A | 1.451 | 0.000 | 0.424 | 0.463 | 0.407 | 1.147 | 1.124 | 1.266 |
| BTBD19 | 1.451 | 0.000 | 0.489 | 0.456 | 0.451 | 1.315 | 1.275 | 1.226 |
| ERLIN1 | 1.457 | 0.000 | 0.691 | 0.576 | 0.573 | 1.608 | 1.715 | 1.730 |
| TXNDC12 | 1.510 | 0.000 | 12.036 | 9.404 | 9.510 | 27.841 | 30.302 | 30.008 |
| TRAM1 | 1.512 | 0.000 | 0.367 | 0.398 | 0.477 | 1.174 | 1.240 | 1.126 |
| MED31 | 1.532 | 0.000 | 0.918 | 1.058 | 0.979 | 2.745 | 2.932 | 2.868 |
| C9orf78 | 1.575 | 0.000 | 0.702 | 0.932 | 0.735 | 2.308 | 2.361 | 2.385 |
| ANAPC16 | 1.576 | 0.000 | 2.766 | 2.750 | 2.677 | 8.072 | 7.945 | 8.404 |
| TMBIM6 | 1.595 | 0.000 | 0.494 | 0.431 | 0.491 | 1.423 | 1.373 | 1.482 |
| MYL6 | 1.608 | 0.000 | 0.238 | 0.290 | 0.248 | 0.785 | 0.766 | 0.814 |
| CDK2AP2 | 1.610 | 0.000 | 0.359 | 0.310 | 0.302 | 0.992 | 0.930 | 1.041 |
| CD300A | 1.674 | 0.000 | 0.203 | 0.174 | 0.151 | 0.579 | 0.533 | 0.573 |
| ARHGDIB | 1.678 | 0.000 | 1.071 | 0.860 | 1.146 | 3.443 | 3.146 | 3.256 |
| PPIB | 1.685 | 0.000 | 5.385 | 5.270 | 5.666 | 18.074 | 16.164 | 18.254 |
| PHF19 | 1.686 | 0.000 | 0.185 | 0.201 | 0.185 | 0.584 | 0.597 | 0.657 |
| TOMM22 | 1.723 | 0.000 | 0.545 | 0.545 | 0.442 | 1.701 | 1.612 | 1.742 |
| CAV1 | 1.725 | 0.000 | 0.968 | 0.733 | 1.093 | 3.120 | 2.951 | 3.167 |
| TCTA | 1.733 | 0.000 | 1.312 | 1.584 | 1.282 | 4.635 | 4.547 | 4.706 |
| NDUFB10 | 1.747 | 0.000 | 1.337 | 1.212 | 1.285 | 4.100 | 4.225 | 4.542 |
| LAPTM5 | 1.760 | 0.000 | 0.185 | 0.205 | 0.199 | 0.668 | 0.656 | 0.671 |
| IGFBP3 | 1.773 | 0.000 | 0.270 | 0.241 | 0.219 | 0.793 | 0.817 | 0.884 |
| SVBP | 1.792 | 0.000 | 0.330 | 0.389 | 0.312 | 1.185 | 1.117 | 1.268 |
| MPG | 1.928 | 0.000 | 0.385 | 0.508 | 0.445 | 1.739 | 1.567 | 1.788 |
| POLR3K | 1.946 | 0.000 | 0.201 | 0.215 | 0.205 | 0.778 | 0.761 | 0.855 |
| EPHX1 | 2.096 | 0.000 | 0.198 | 0.242 | 0.151 | 0.809 | 0.817 | 0.899 |
| ZCRB1 | 2.145 | 0.000 | 0.223 | 0.227 | 0.194 | 0.936 | 0.939 | 0.971 |
| TXN | 2.148 | 0.000 | 0.549 | 0.589 | 0.660 | 2.680 | 2.483 | 2.810 |
| TSPAN13 | 2.175 | 0.000 | 0.236 | 0.270 | 0.393 | 1.341 | 1.371 | 1.348 |
| LPCAT2 | 2.219 | 0.000 | 0.426 | 0.585 | 0.667 | 2.478 | 2.763 | 2.572 |
| SLC48A1 | 2.276 | 0.000 | 0.542 | 0.737 | 0.574 | 3.011 | 3.014 | 2.944 |
| SFT2D1 | 2.287 | 0.000 | 0.511 | 0.609 | 0.449 | 2.632 | 2.314 | 2.712 |
| ITGB3BP | 2.301 | 0.000 | 0.202 | 0.165 | 0.215 | 0.988 | 0.945 | 0.933 |
| CDC26 | 2.511 | 0.000 | 0.390 | 0.594 | 0.707 | 2.987 | 3.452 | 3.200 |
| CD74 | 2.553 | 0.000 | 0.453 | 0.489 | 0.341 | 2.493 | 2.314 | 2.719 |
| NUDT2 | 2.557 | 0.000 | 0.357 | 0.407 | 0.392 | 2.284 | 2.140 | 2.382 |
| MOSPD3 | 2.743 | 0.000 | 0.242 | 0.272 | 0.332 | 2.004 | 1.952 | 1.708 |
| SLC7A7 | 2.790 | 0.000 | 0.125 | 0.313 | 0.241 | 1.443 | 1.589 | 1.667 |
| STOML1 | 2.912 | 0.000 | 0.095 | 0.122 | 0.043 | 0.610 | 0.672 | 0.674 |
| HMOX1 | 2.998 | 0.000 | 0.193 | 0.259 | 0.155 | 1.549 | 1.592 | 1.701 |
| LEFTY2 | 3.010 | 0.000 | 0.101 | 0.147 | 0.145 | 1.000 | 1.018 | 1.148 |
| CR2 | 3.344 | 0.000 | 0.121 | 0.156 | 0.072 | 1.204 | 1.120 | 1.221 |
| MLC1 | 3.427 | 0.000 | 0.086 | 0.383 | 0.079 | 1.972 | 1.870 | 2.045 |
| SULF2 | 3.815 | 0.000 | 0.112 | 0.299 | 0.075 | 2.342 | 2.180 | 2.322 |
| LILRB4 | 4.106 | 0.000 | 0.471 | 0.193 | 0.538 | 6.697 | 6.375 | 7.632 |
| GALM | 4.432 | 0.000 | 0.077 | 0.033 | 0.041 | 1.057 | 1.018 | 1.195 |
| APCDD1 | 4.624 | 0.000 | 0.159 | 0.000 | 0.241 | 3.272 | 3.506 | 3.078 |
