# Supplementary material for: METTL5 regulates SEPHS2-mediated selenoprotein synthesis to promote multiple myeloma survival and progression
Source: Cell Death Dis. 2025 Aug 2;16(1):585. doi: 10.1038/s41419-025-07904-6 (PMC12316883; doi:10.1038/s41419-025-07904-6)
Supplement: Supplementary file 2 — original data [file 41419_2025_7904_MOESM2_ESM.pdf]

METTL5 regulates SEPHS2-mediated selenoprotein synthesis to promote multiple myeloma survival and progression

Original data

Figure 1C

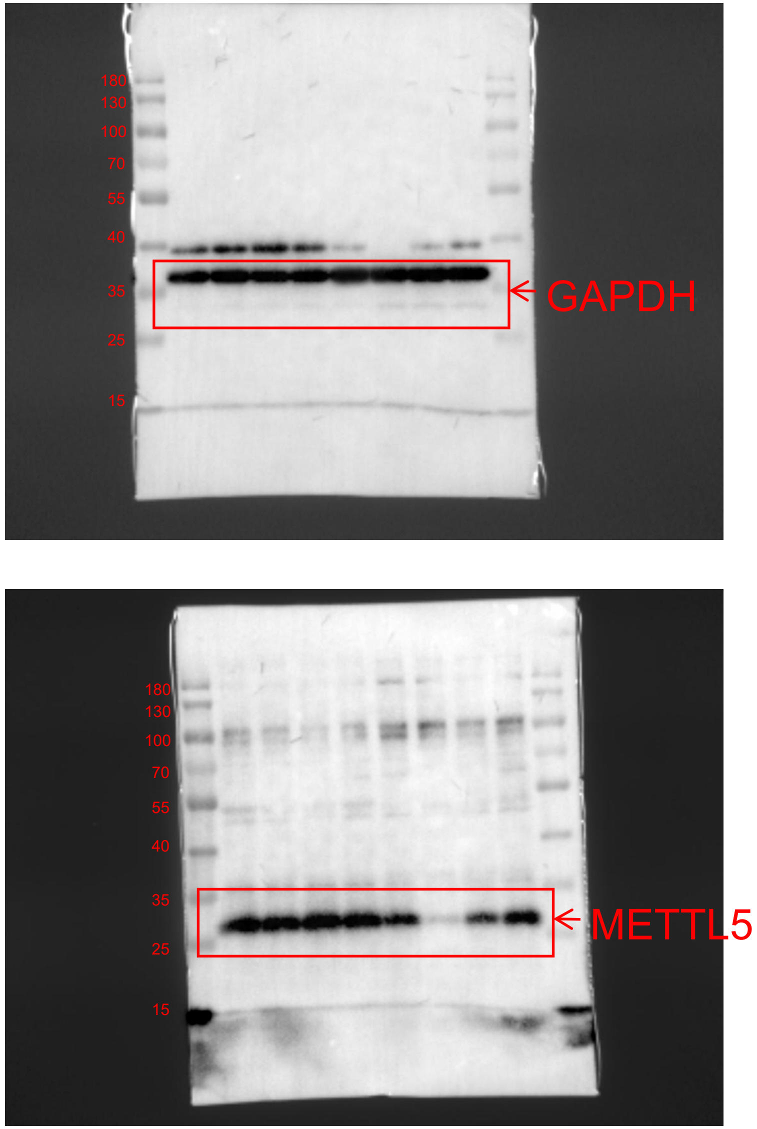

Figure 1D

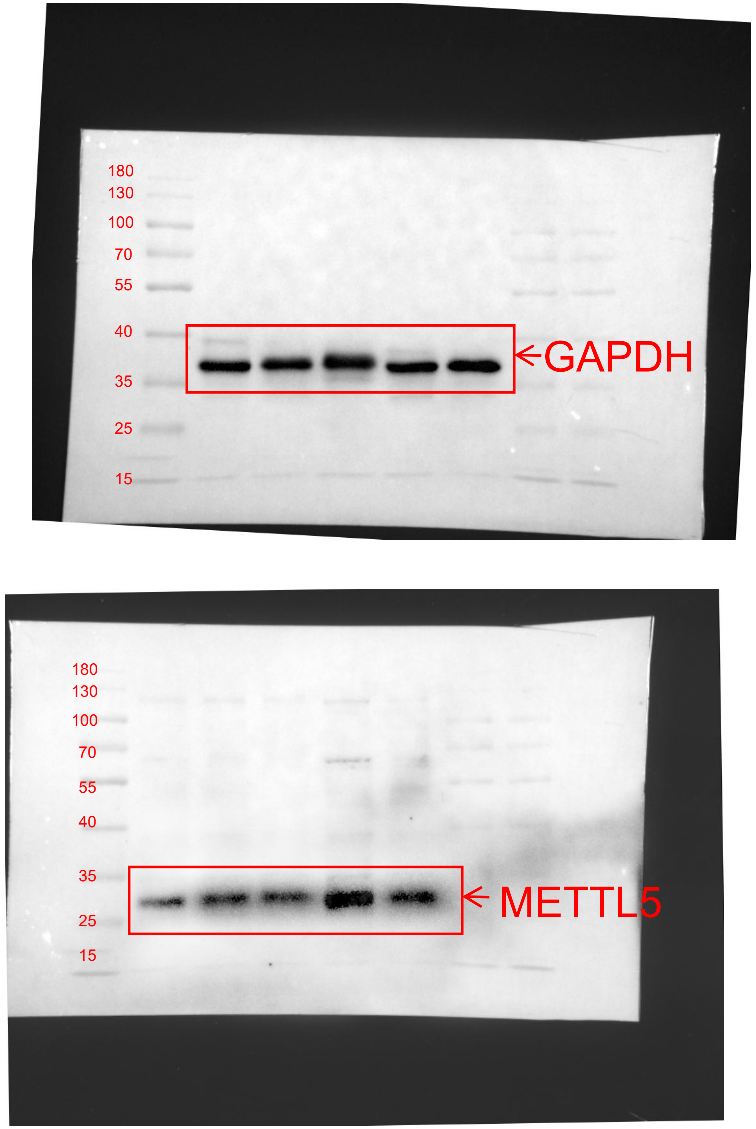

Figure 2B

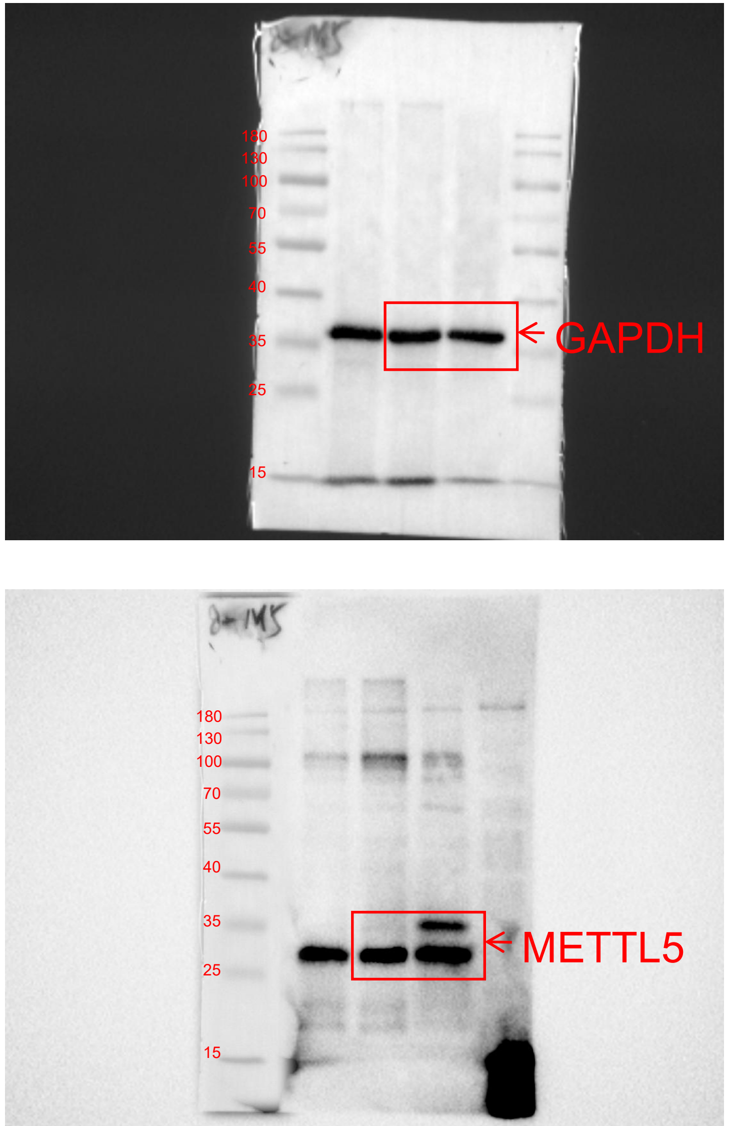

Figure 3B

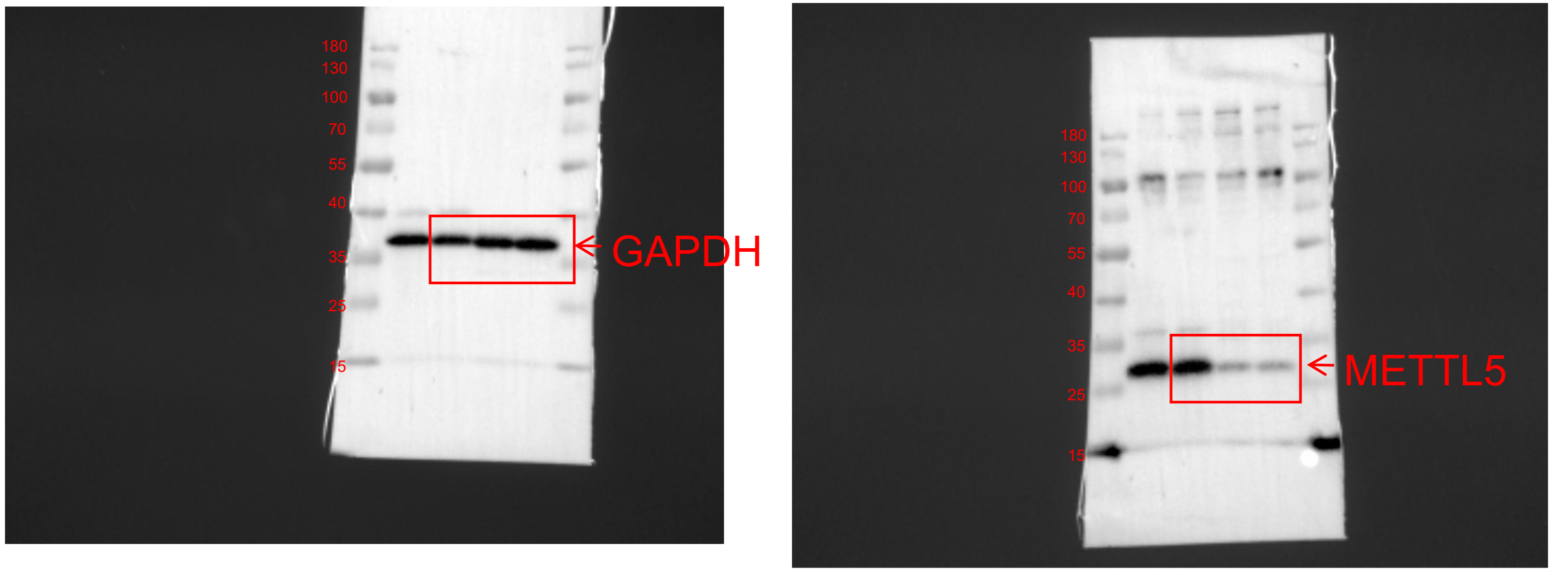

Figure 4A

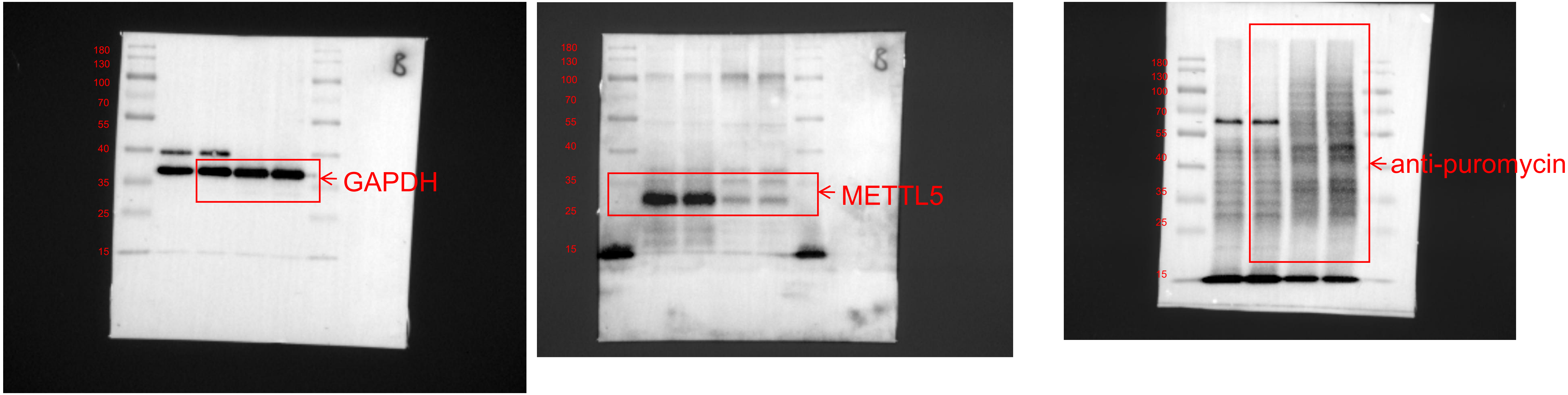

Figure 4B

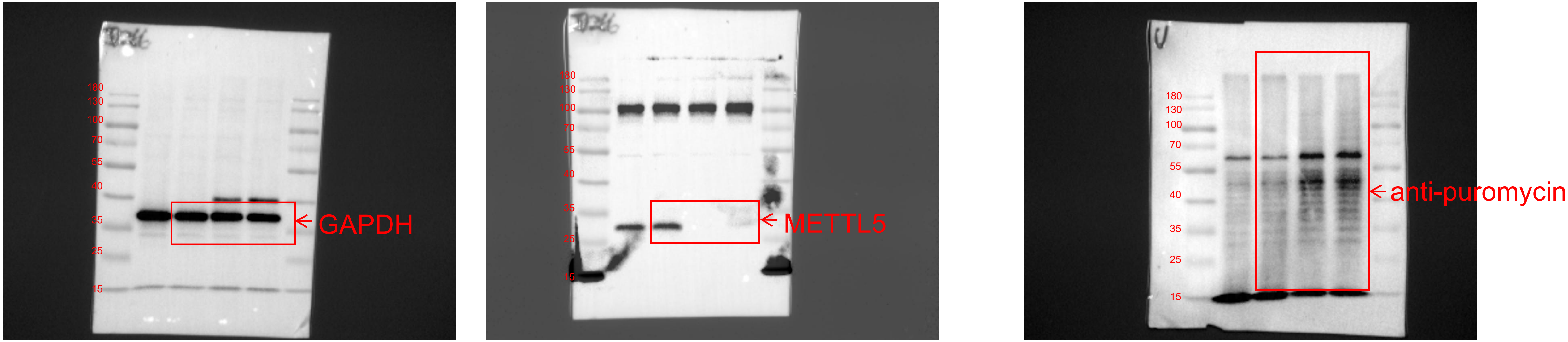

Figure 4H

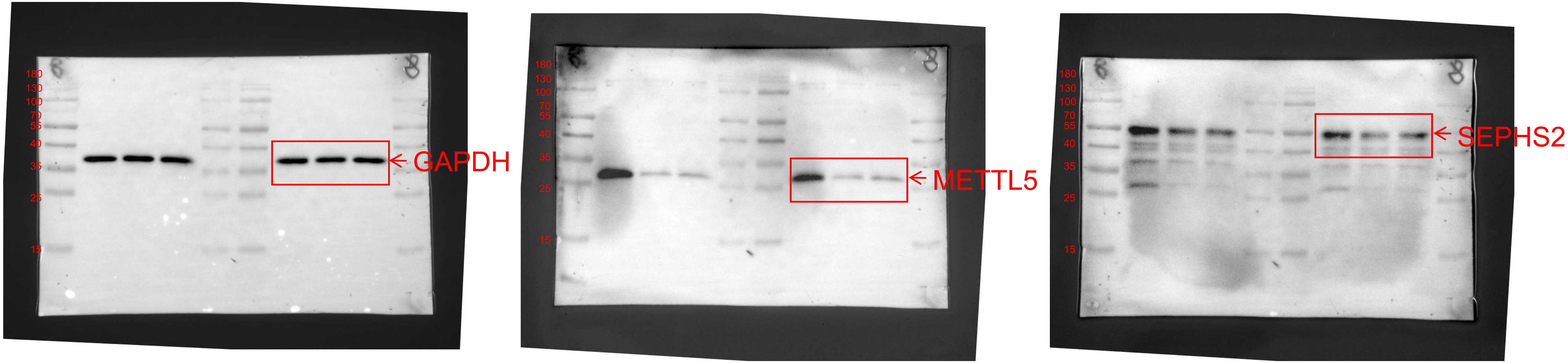

Figure 4I

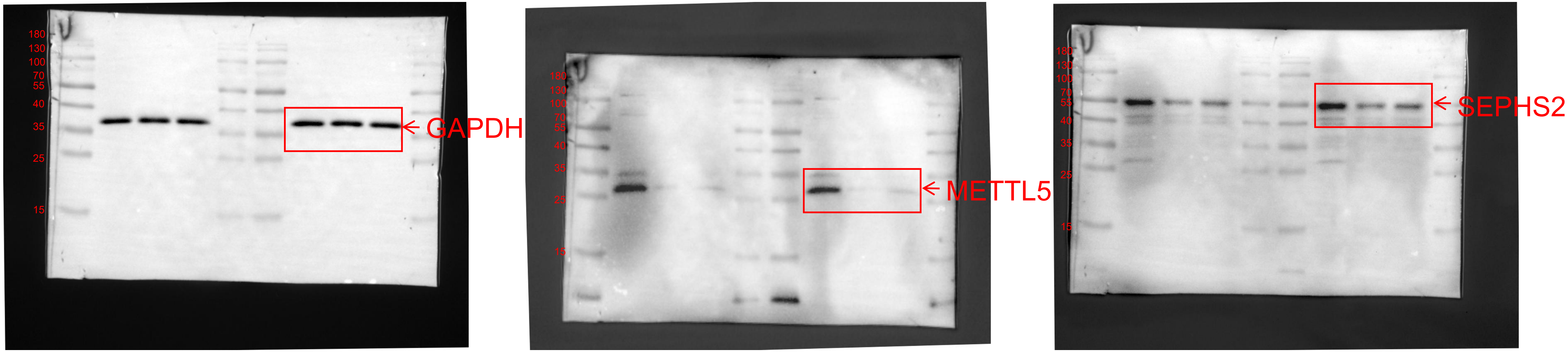

Figure 5B

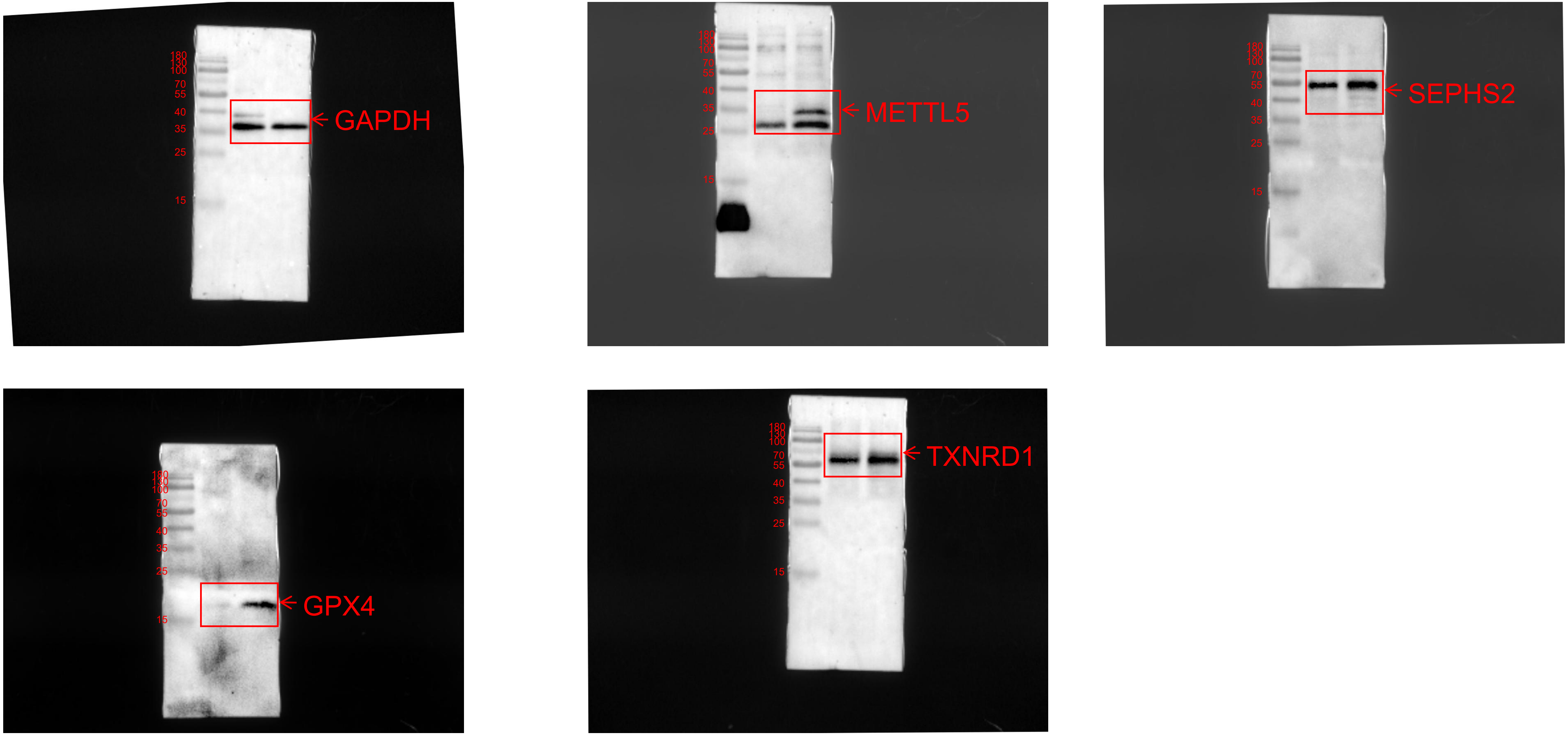

Figure 5D

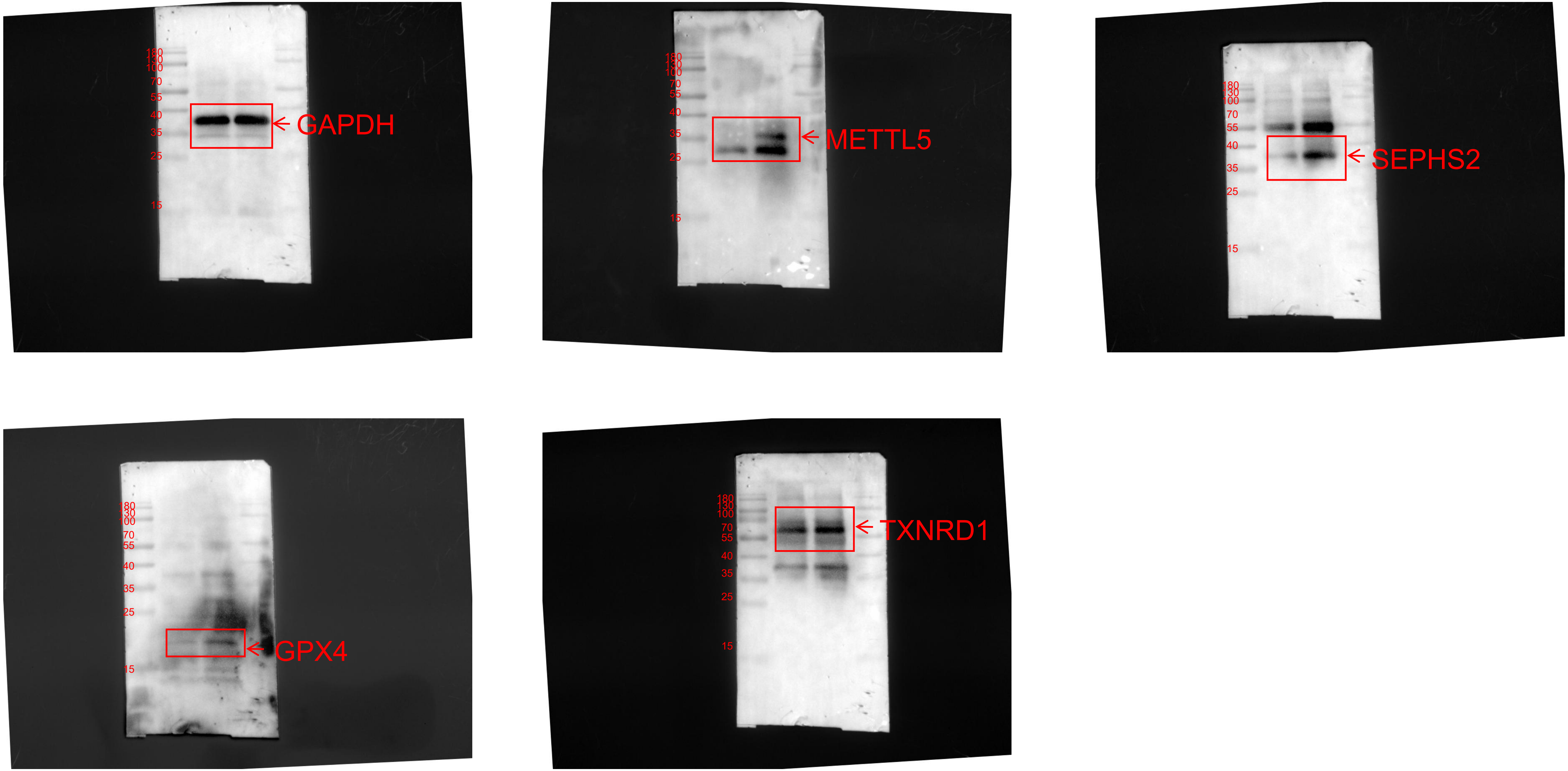

Figure 5F

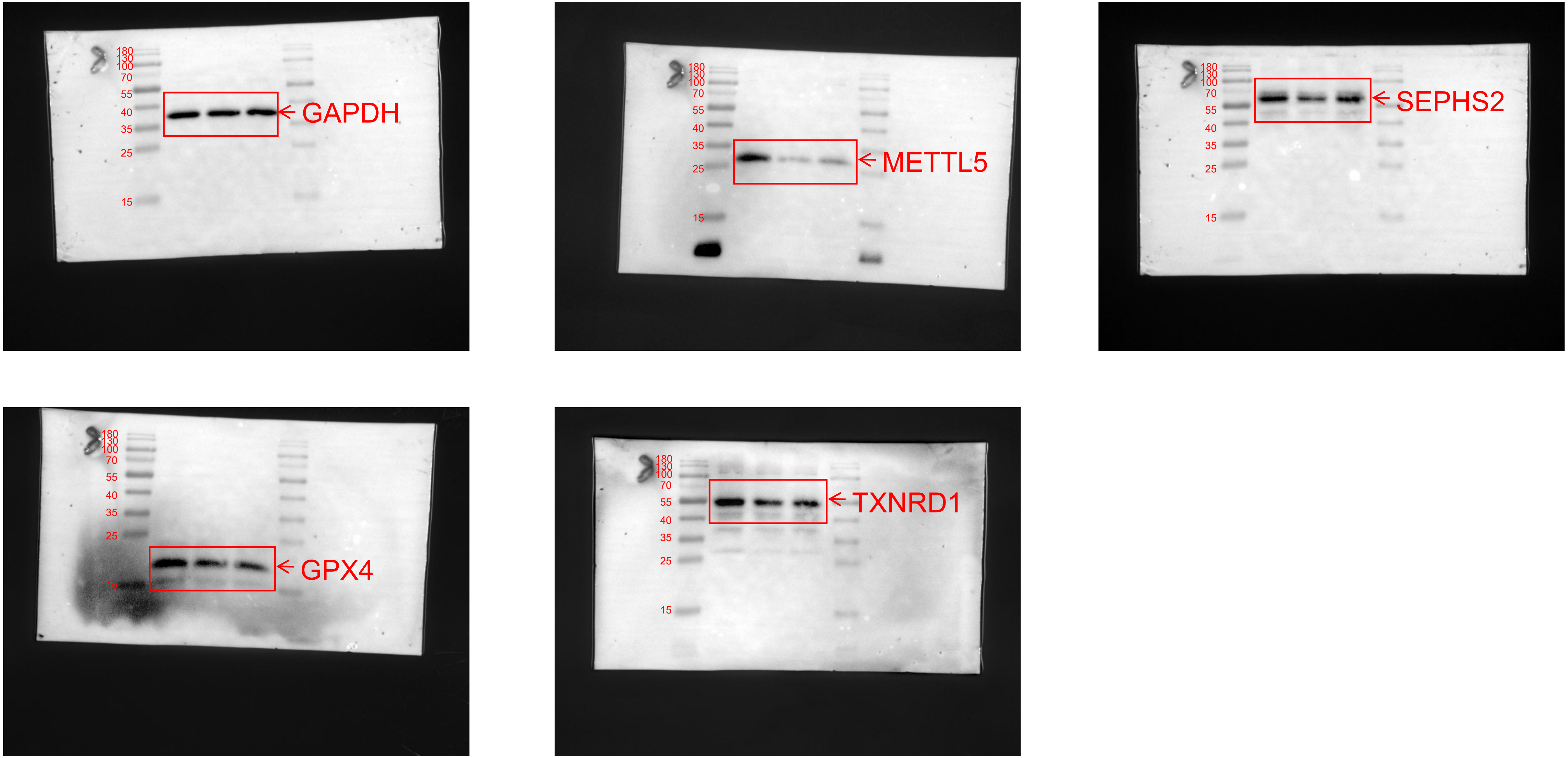

Figure 5H

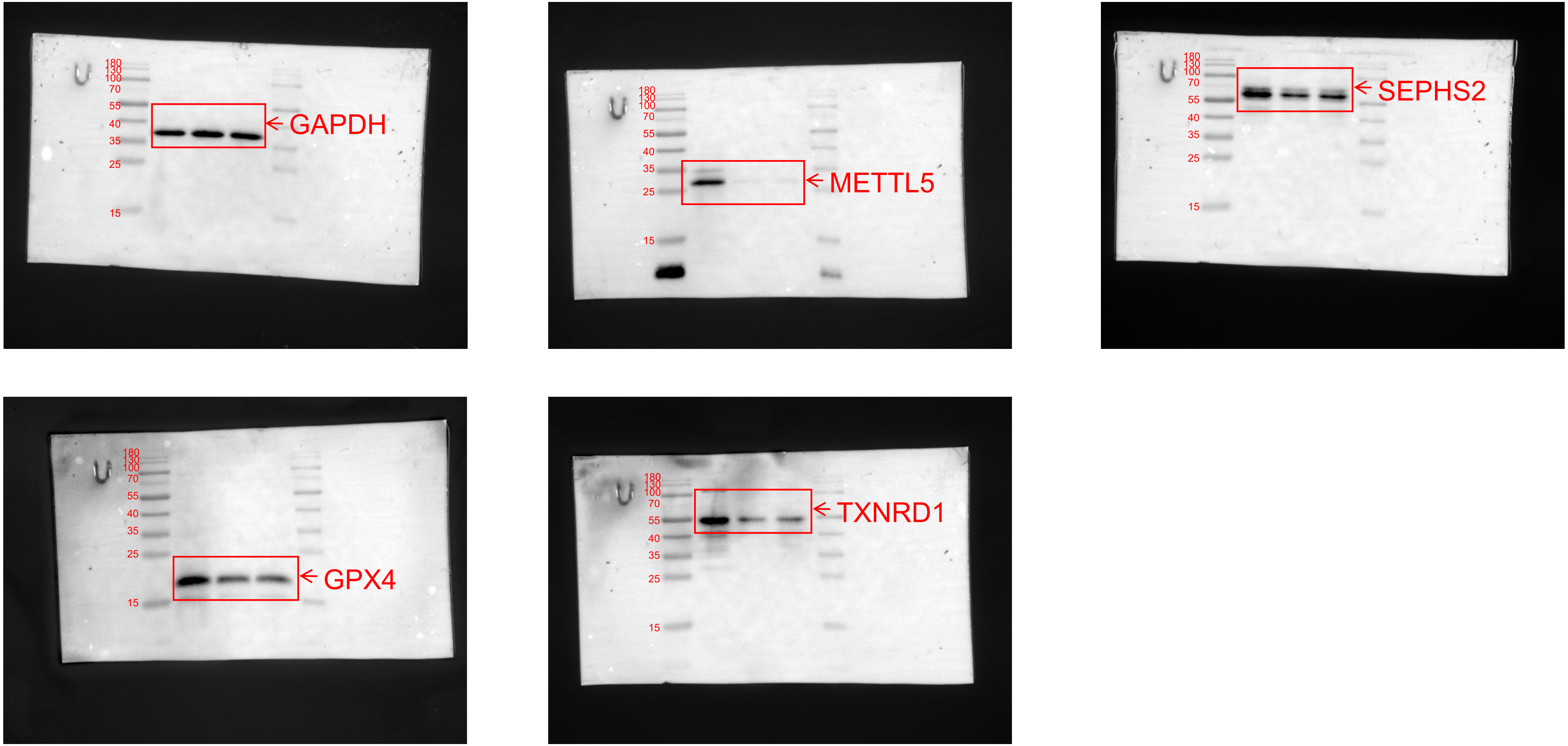

Figure 5M

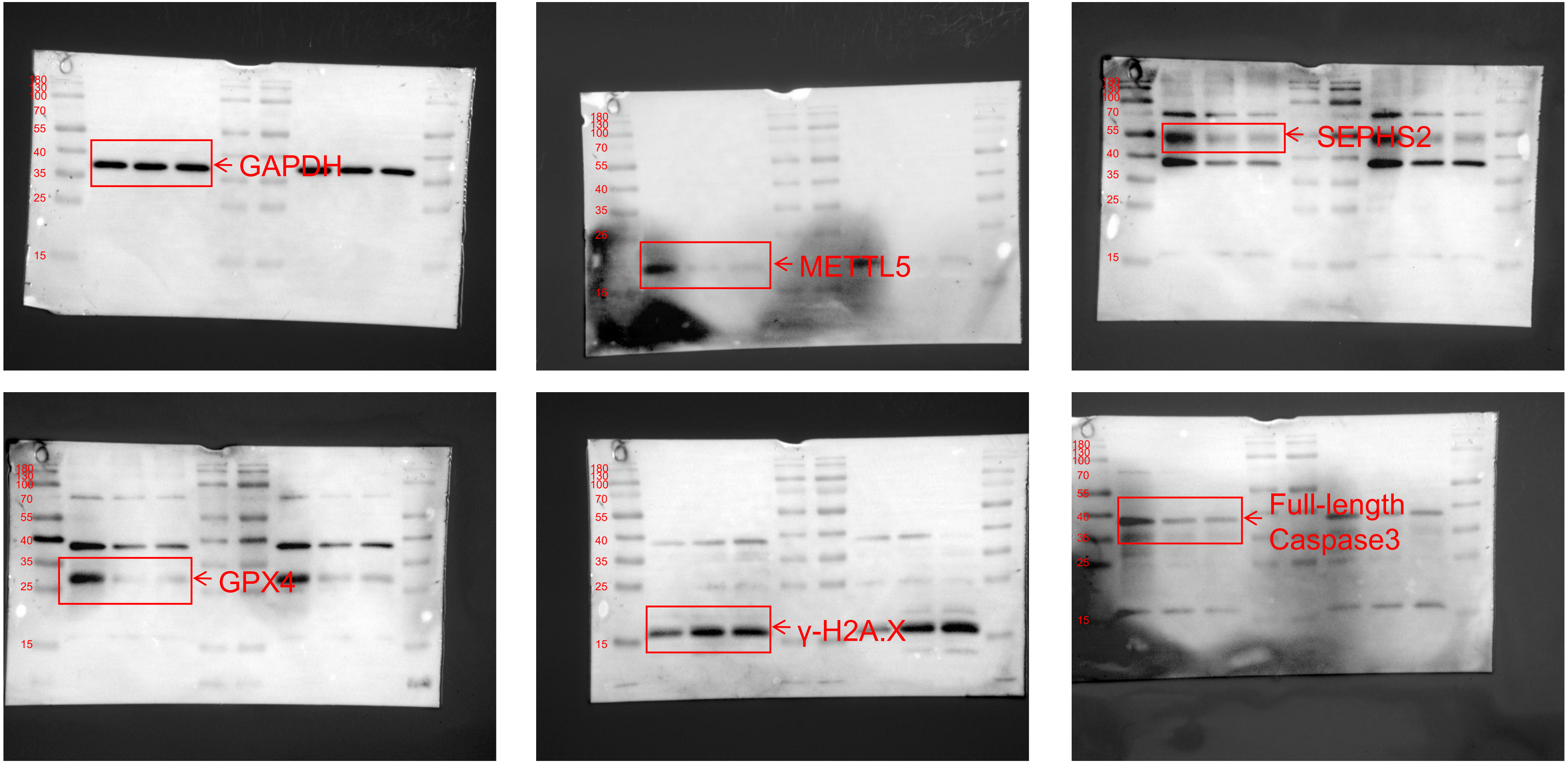
180
130
100
70
55
40
35
25
15
GAPDH
METTL5
SEPHS2
GPX4
γ-H2A.X
Full-length
Caspase3

Figure 5M

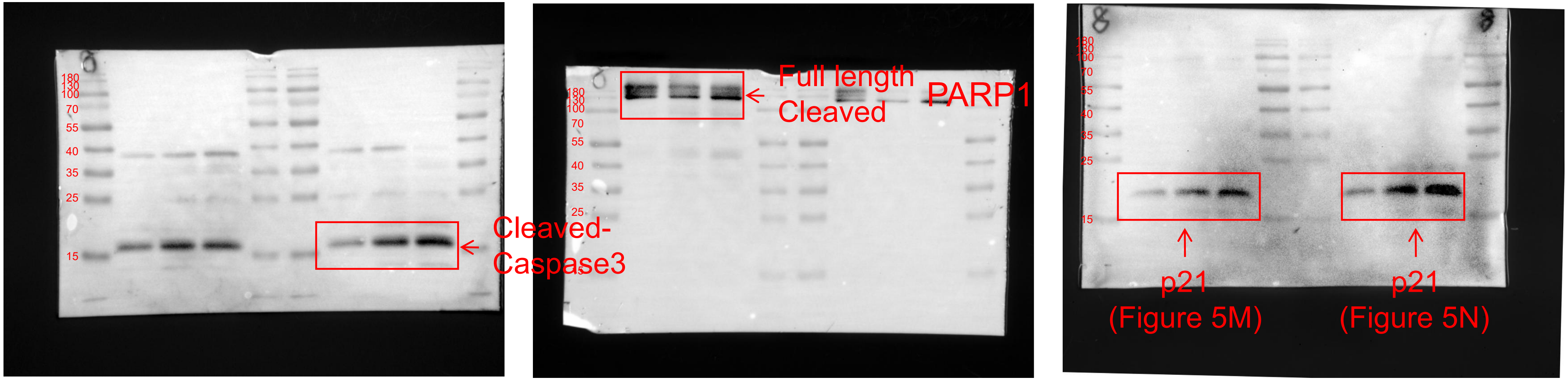

Figure 5N

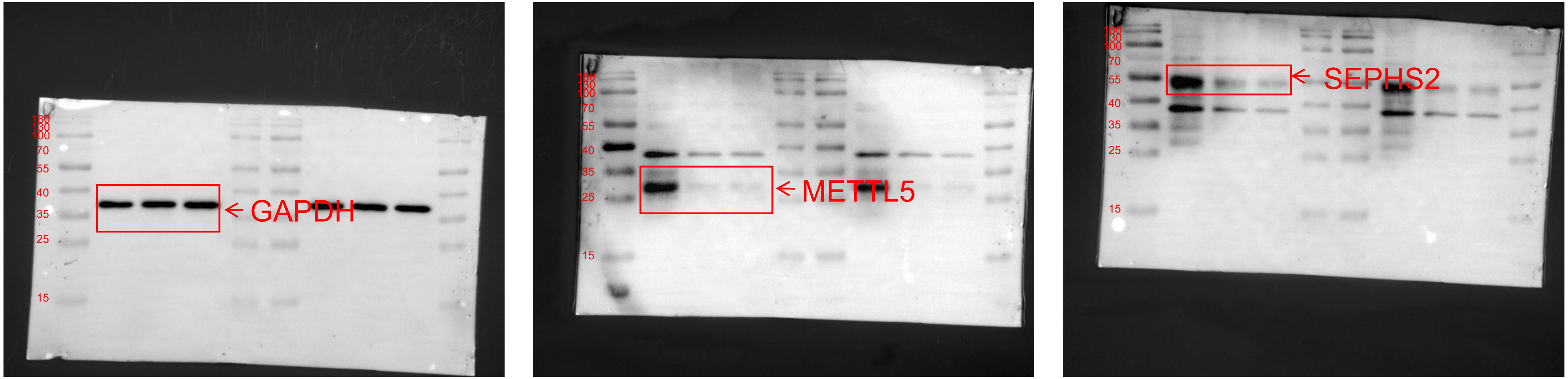

Figure 5N

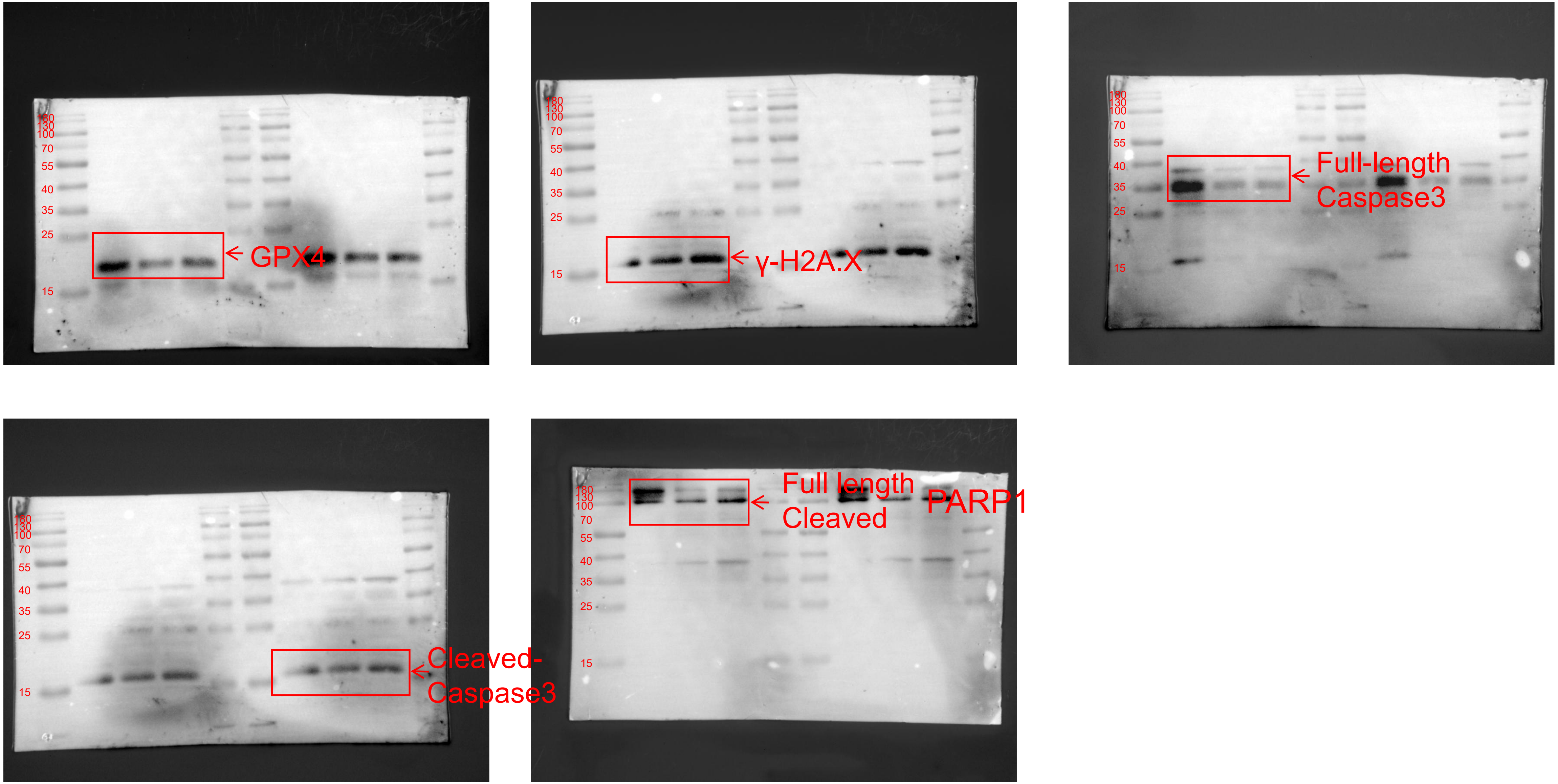
180
130
100
70
55
40
35
25
15
GPX4
γ-H2A.X
Full-length
Caspase3
Cleaved-
Caspase3
Full length
Cleaved
PARP1

Figure 6D

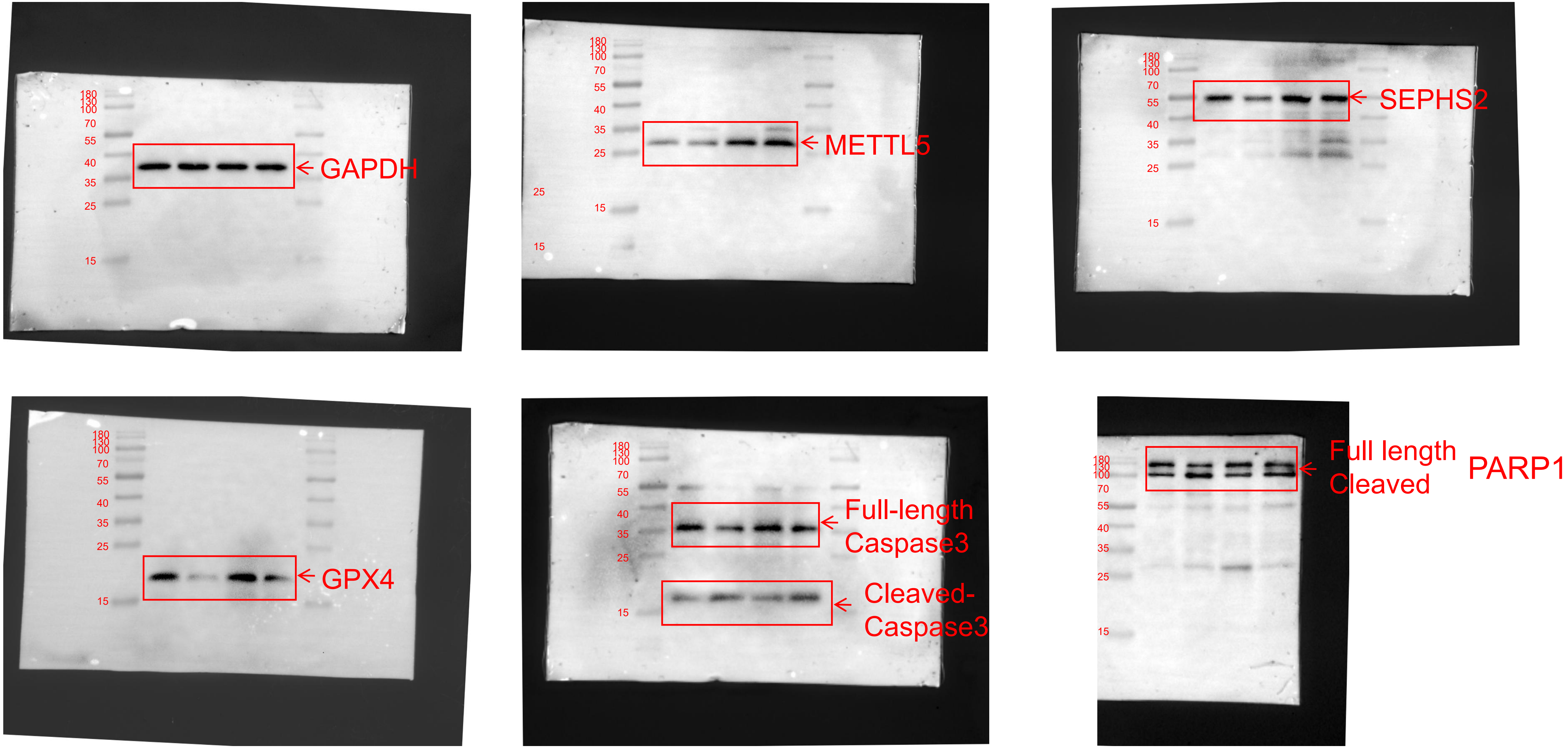
180
130
100
70
55
40
35
25
15
GAPDH
METTL5
SEPHS2
GPX4
Full-length
Caspase3
Cleaved-
Caspase3
Full length
Cleaved
PARP1

Figure 6I

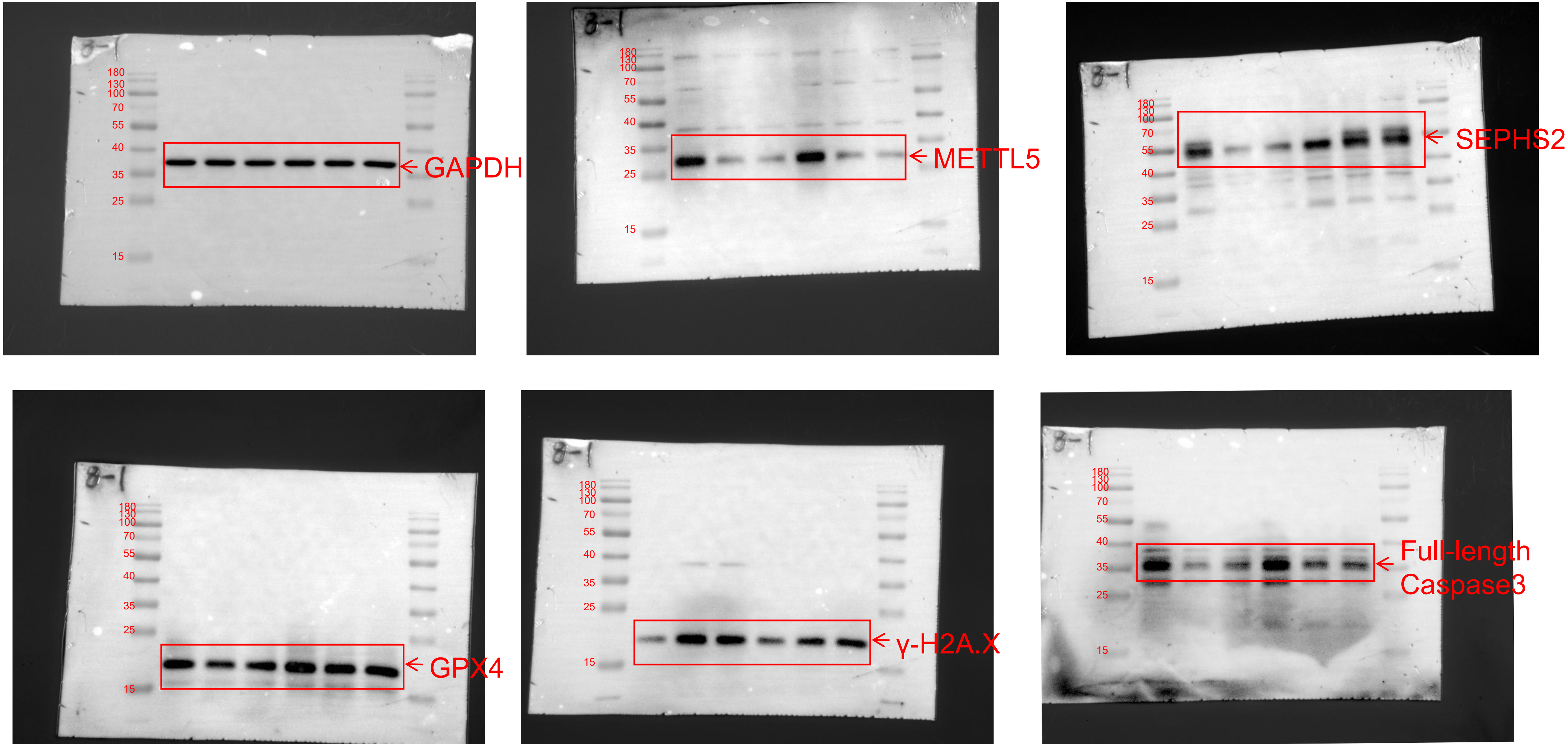

180
130
100
70
55
40
35
25
15
GAPDH
METTL5
SEPHS2
GPX4
γ-H2A.X
Full-length
Caspase3

Figure 6I

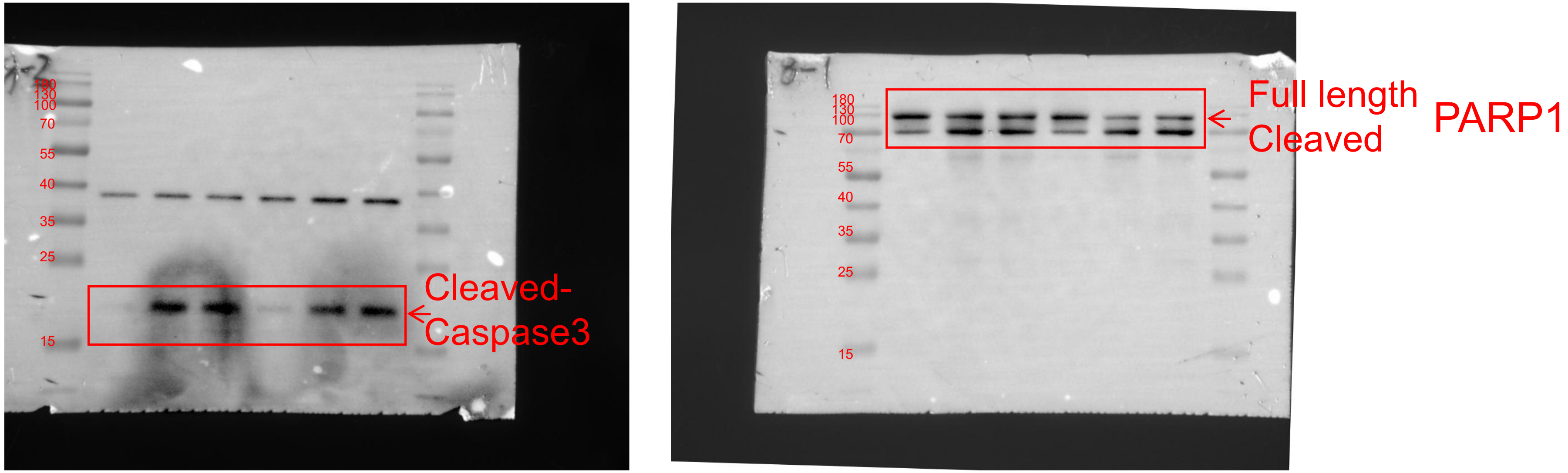

180
130
100
70
55
40
35
25
15
Cleaved-
Caspase3
Full length
Cleaved
PARP1

Figure 7F

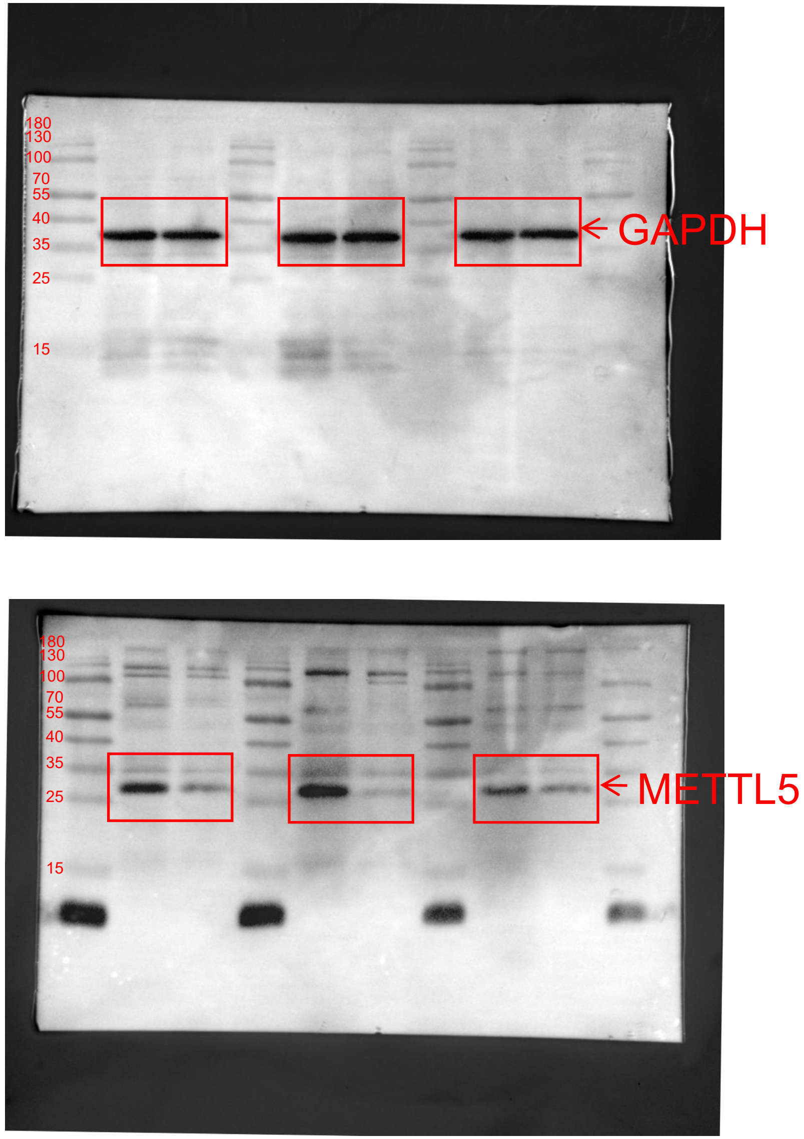

Figure 7J and K

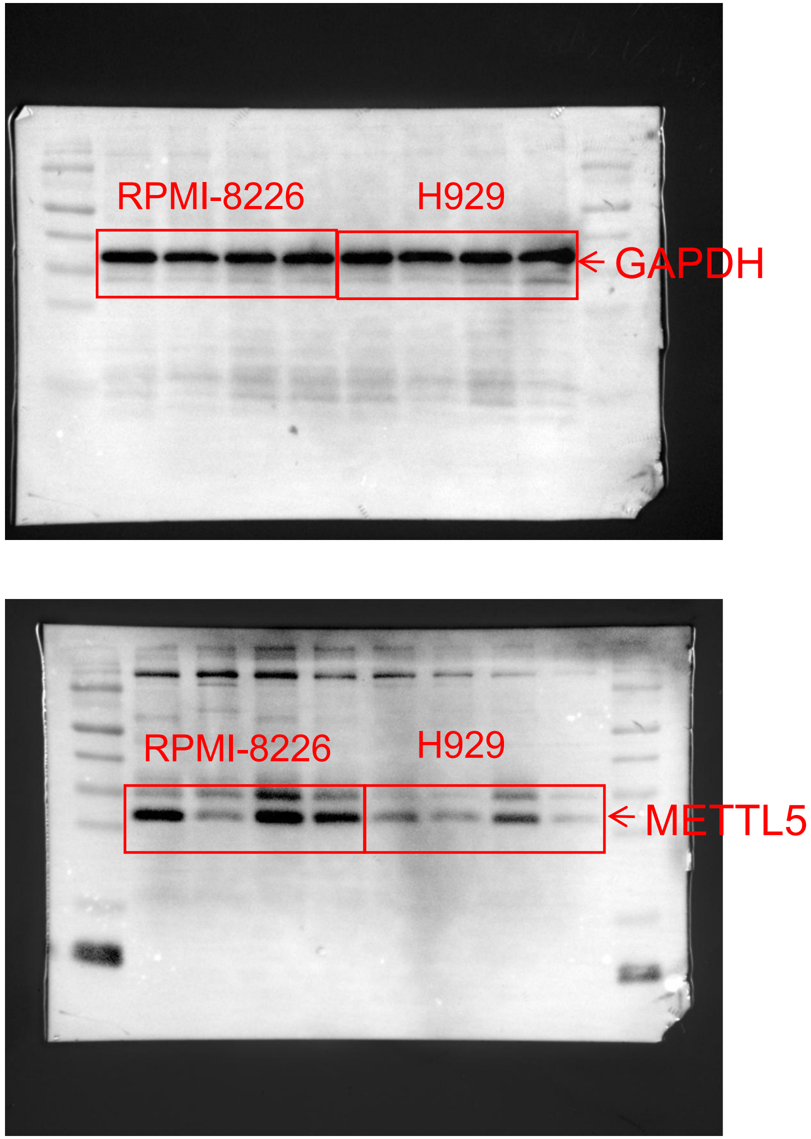

Figure 7I

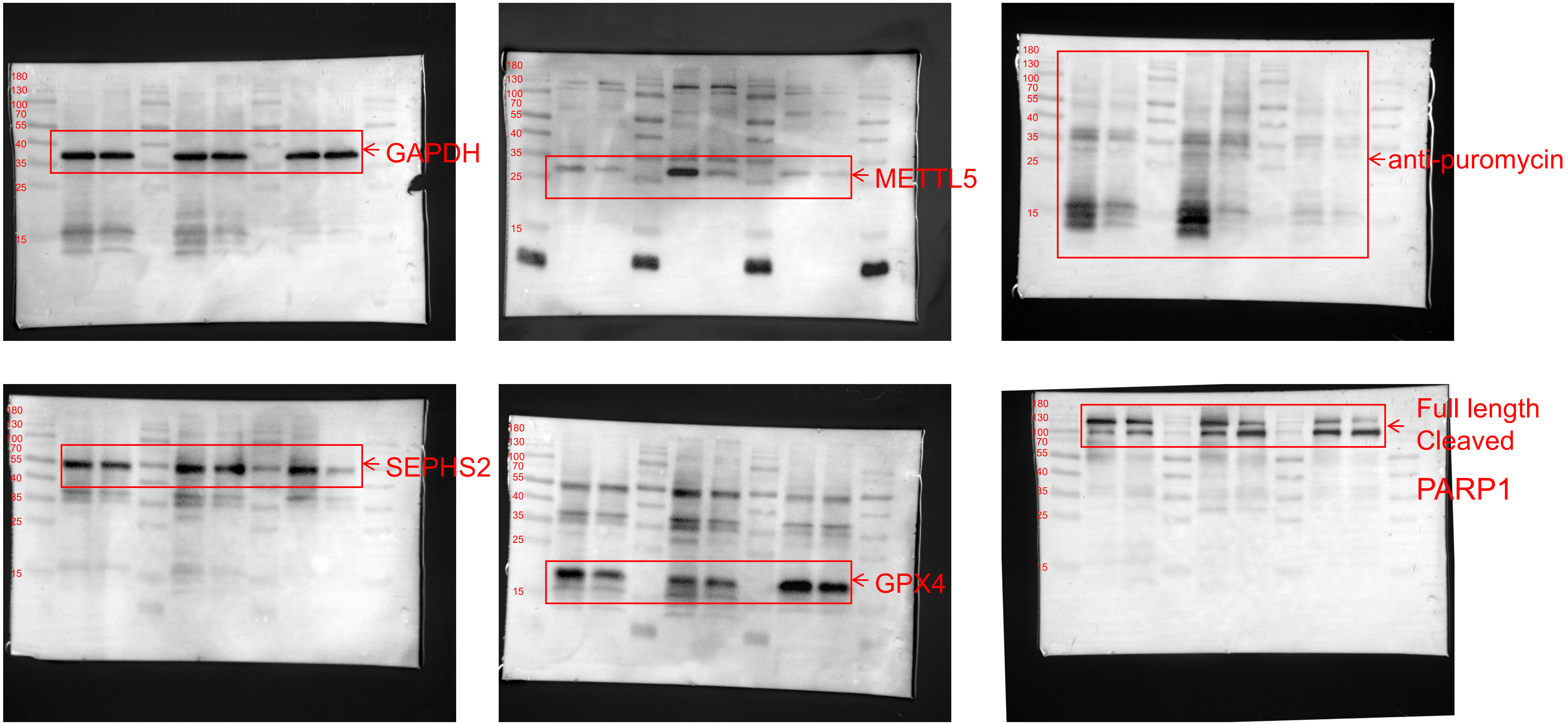
180
130
100
70
55
40
35
25
15
GAPDH
METTL5
anti-puromycin
SEPHS2
GPX4
Full length
Cleaved
PARP1

Figure S2B

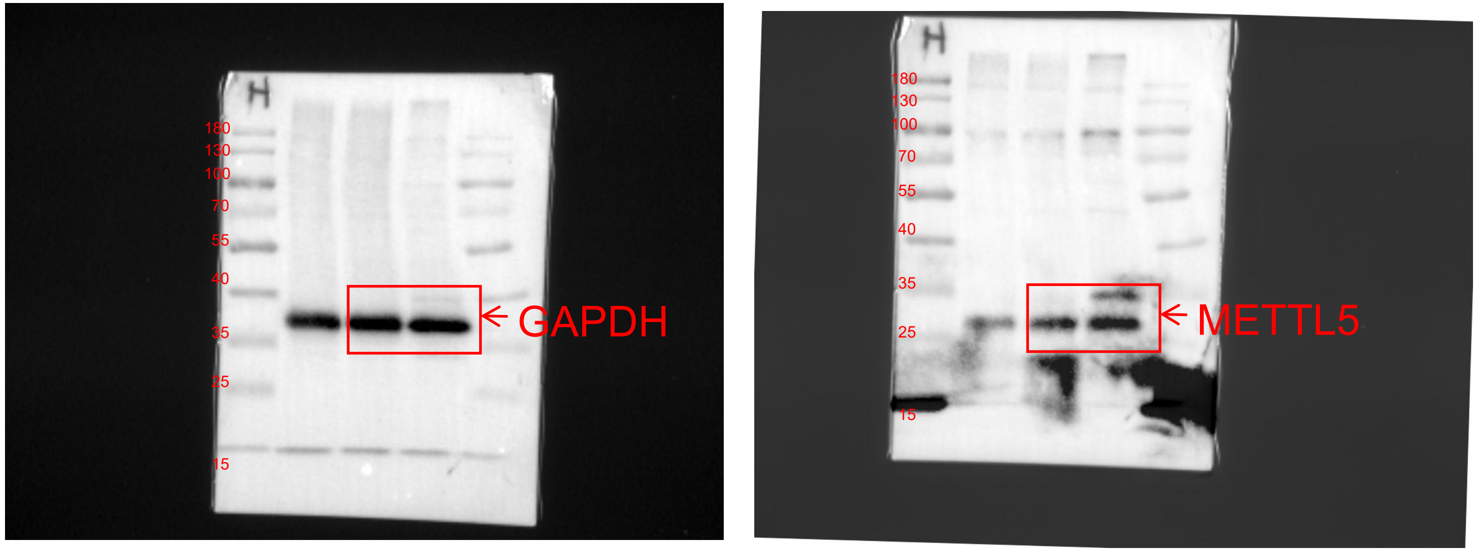

Figure S4A

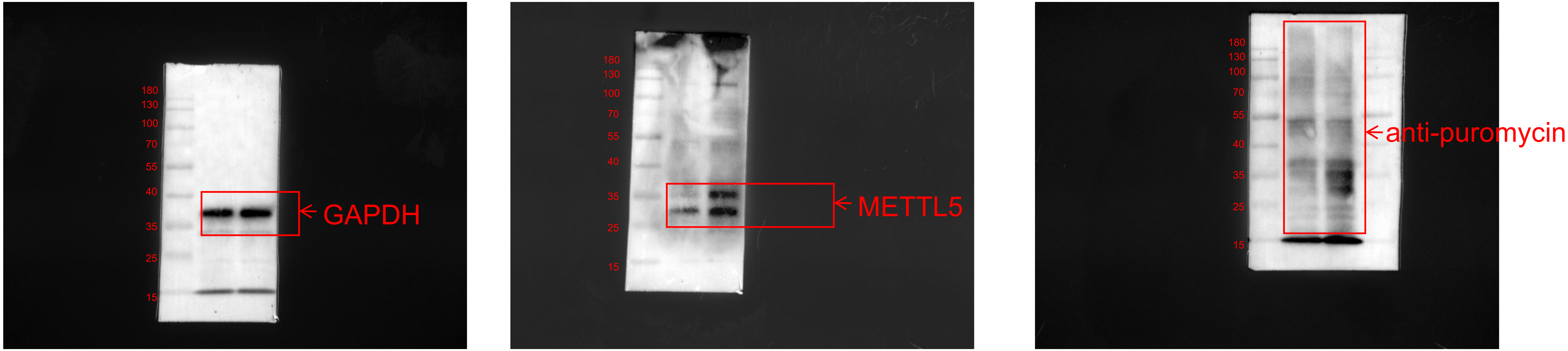

Figure S4B

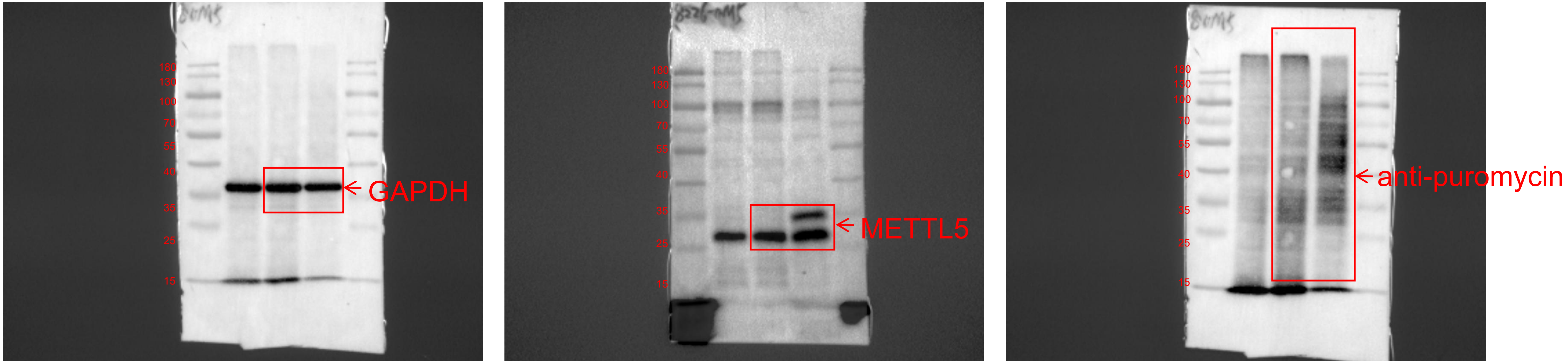

Figure S5C

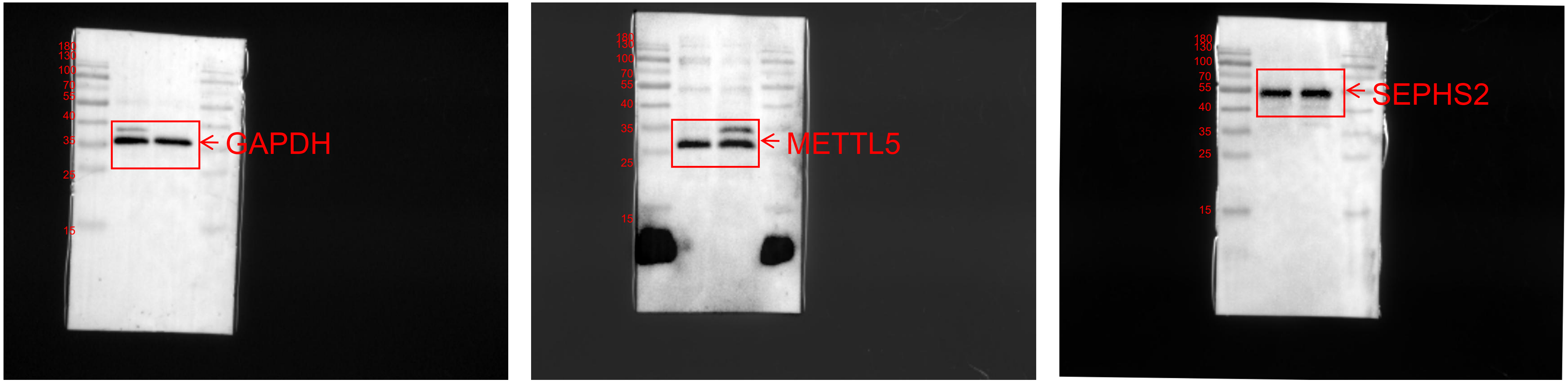

Figure S5D

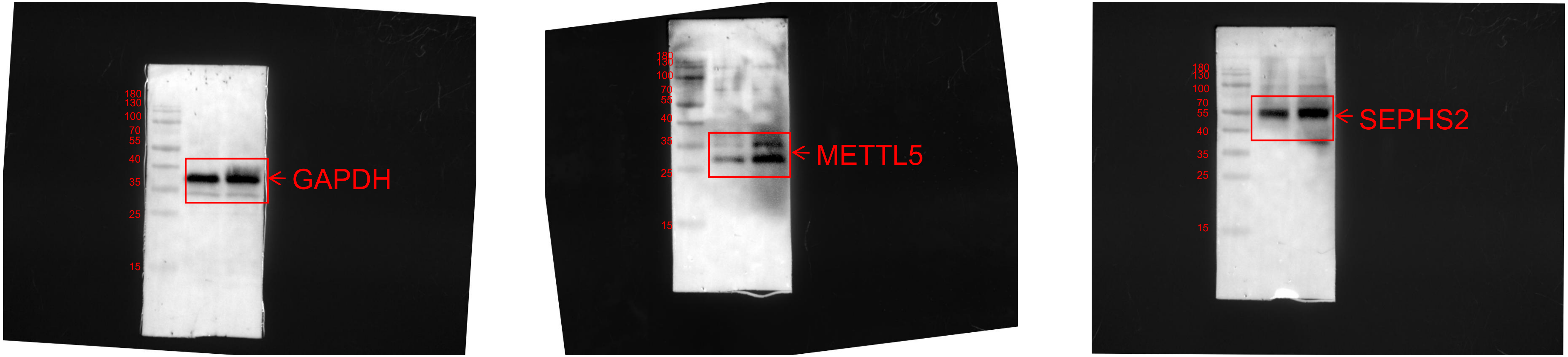

Figure S6B

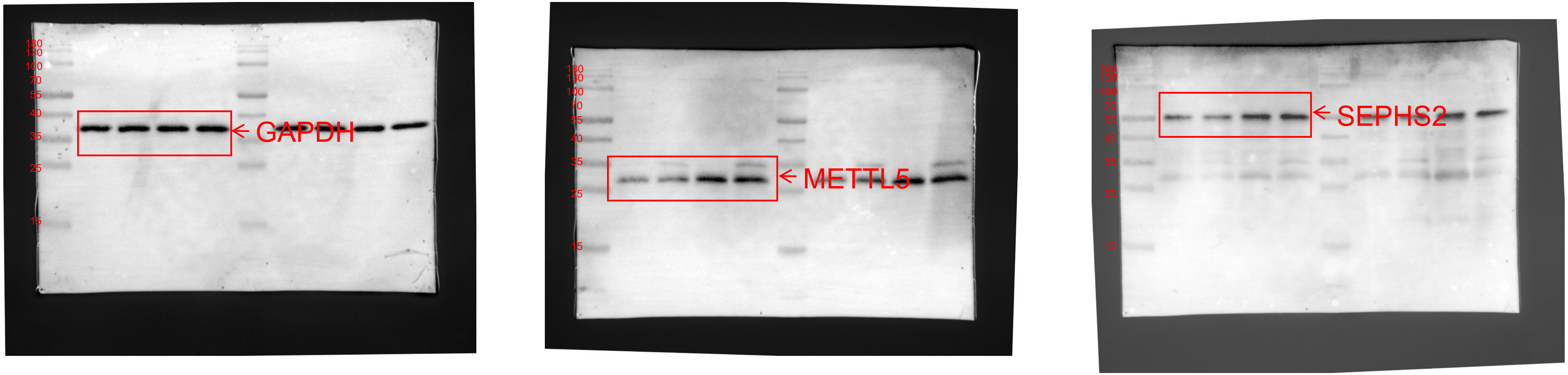

Figure S6D

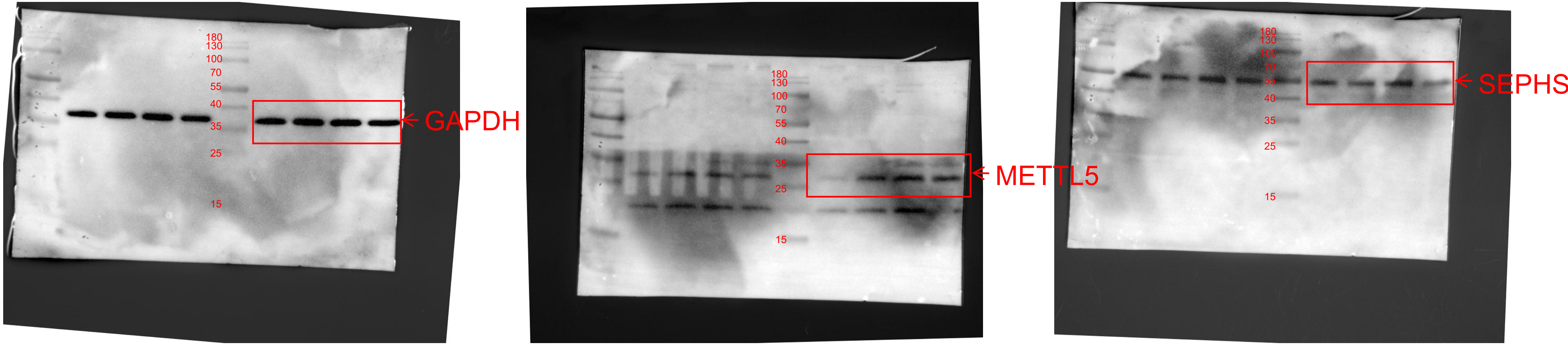

Figure S6G

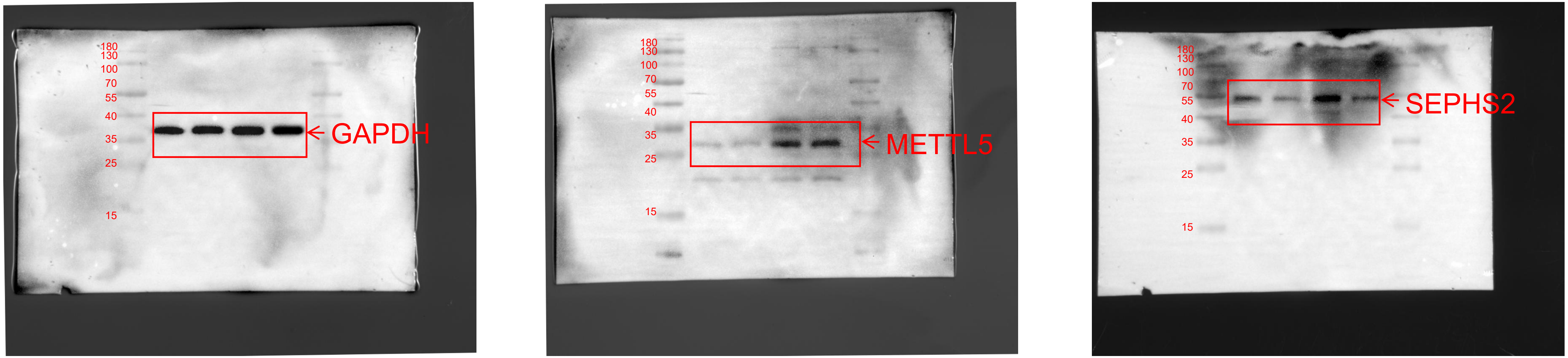

Figure S6G

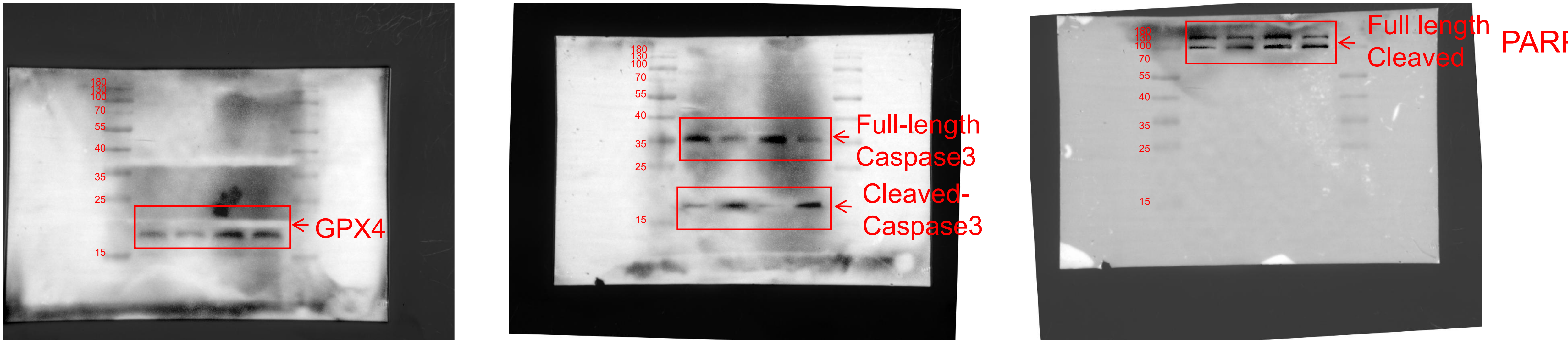
180
130
100
70
55
40
35
25
15
GPX4
Full-length
Caspase3
Cleaved-
Caspase3
Full length
Cleaved
PARP

Figure S7B

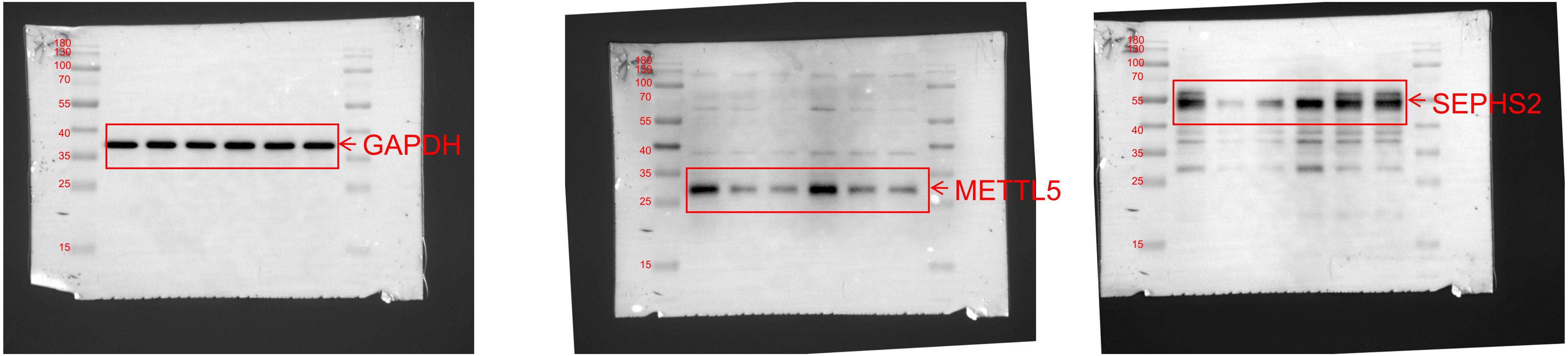

Figure S7D

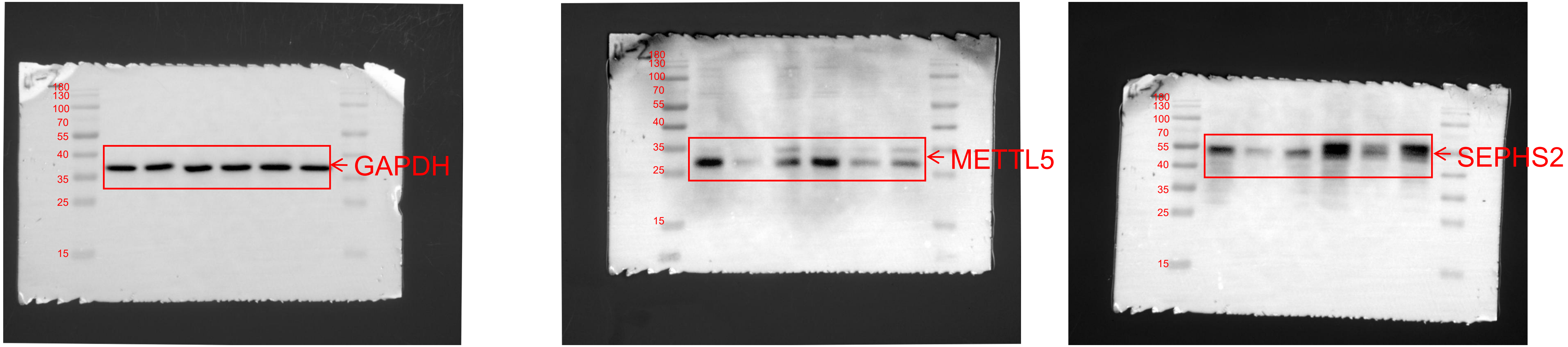

Figure S7K

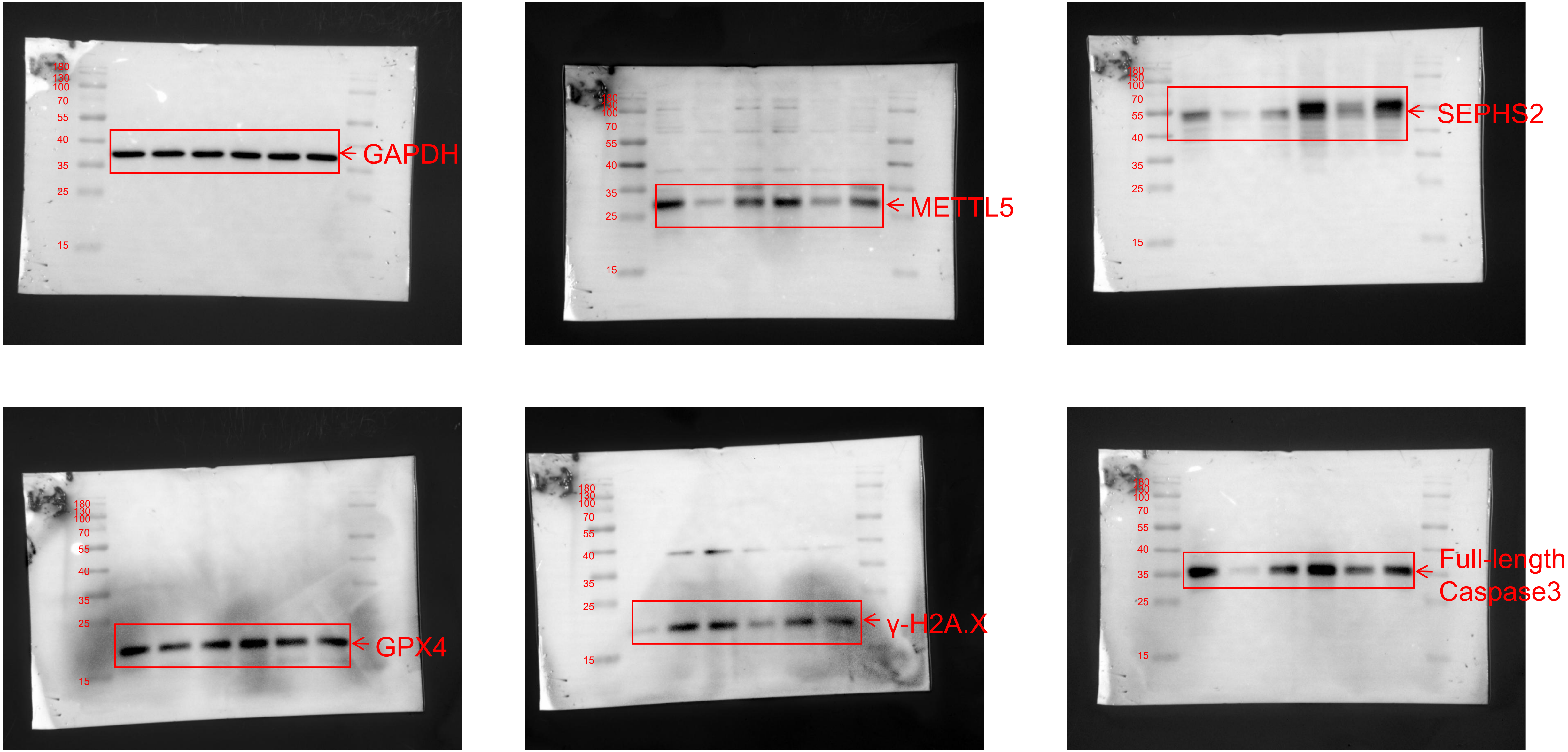

180
130
100
70
55
40
35
25
15
GAPDH
METTL5
SEPHS2
GPX4
γ-H2A.X
Full-length
Caspase3

Figure S7K

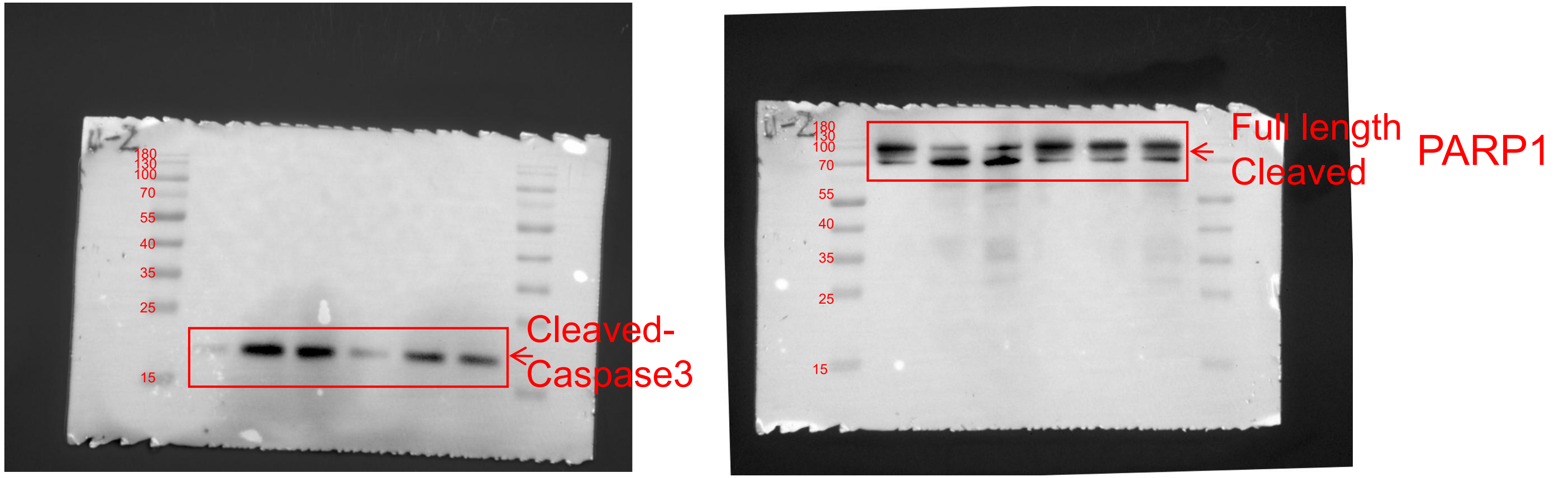

Figure S9K and L

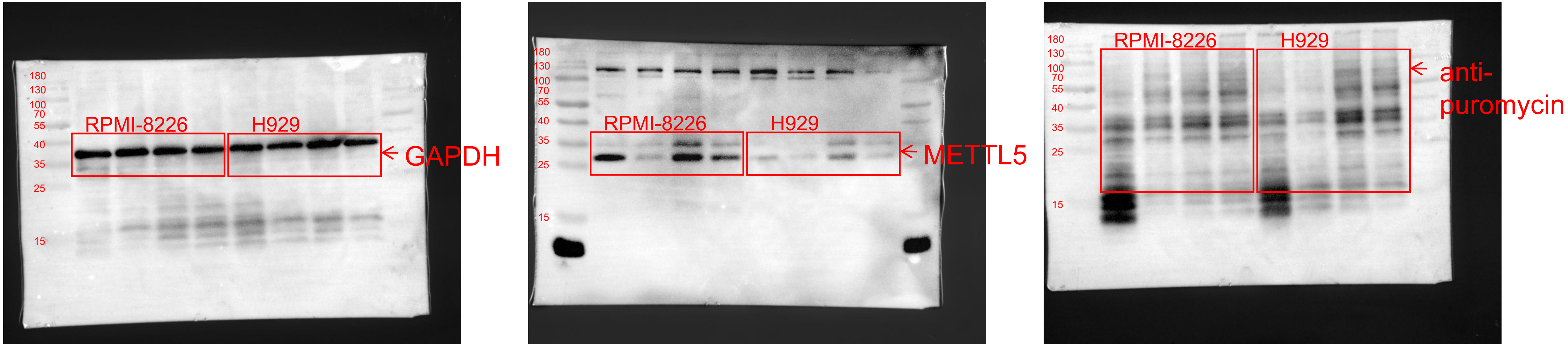

Figure S9K and L

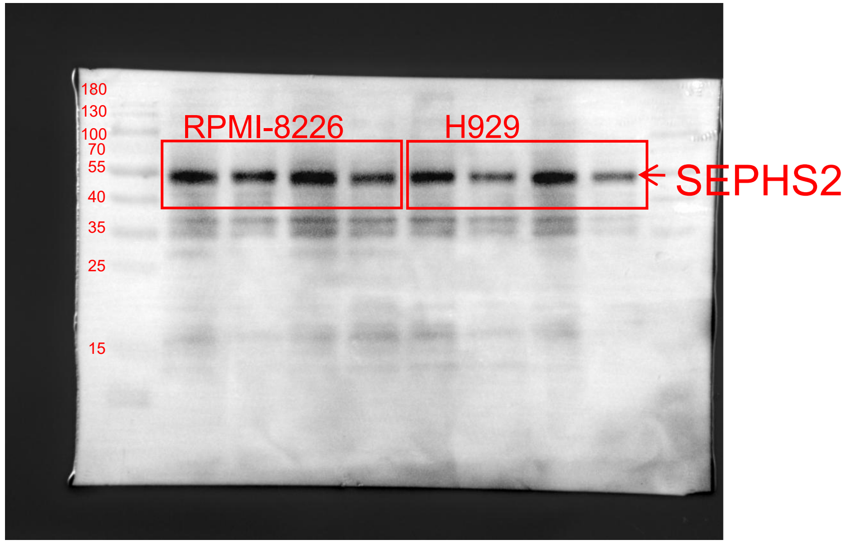

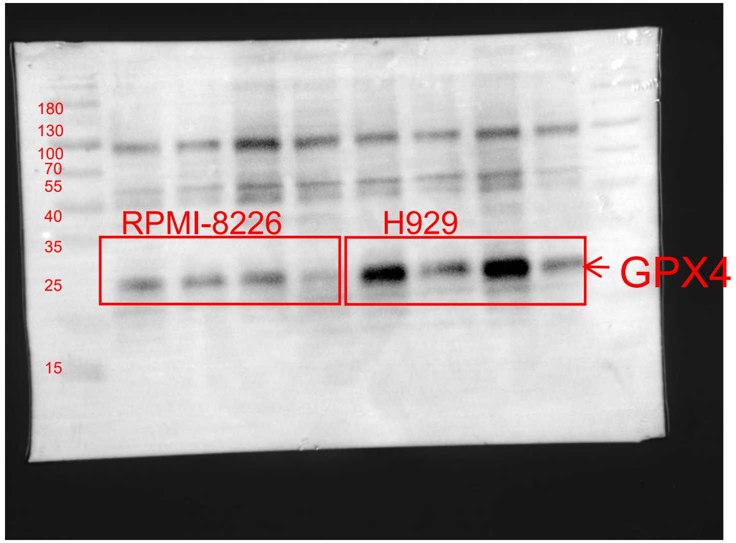

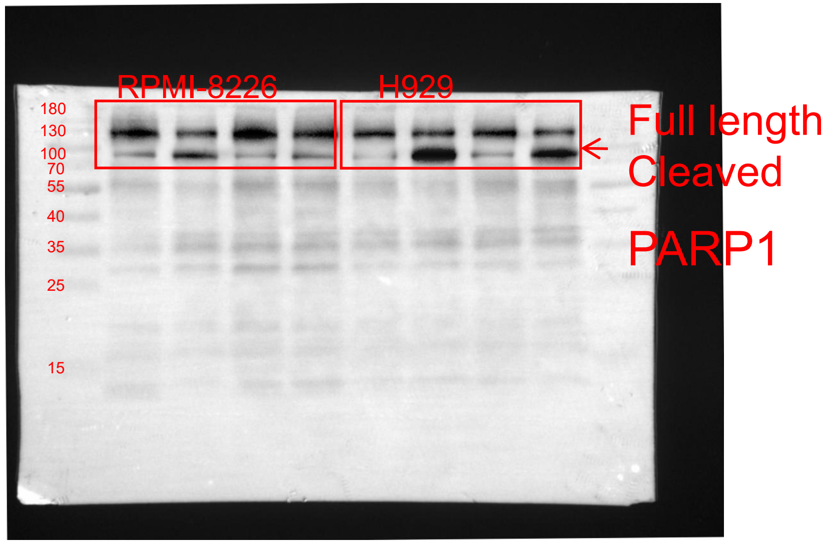

Figure 1B

MM-PD	GAPDH	METTL5	MM-CR	GAPDH	METTL5
M1	14.030	17.647	C1	13.976	21.315
M2	14.963	19.897	C2	14.715	23.222
M3	13.182	19.629	C3	14.366	23.263
M4	14.159	19.930	C4	14.777	23.173
M5	15.844	22.799	C5	14.936	23.610
M6	15.451	20.876	C6	14.855	22.881
M7	13.071	17.888	C7	14.881	23.310
M8	14.700	18.603	C8	15.091	22.222
M9	14.524	18.810	C9	15.008	26.688
M10	13.996	18.967	C10	14.823	22.999
M11	13.698	17.443	C11	13.993	24.915
M12	15.831	20.100			
M13	14.475	19.204			
M14	14.691	18.709			
M15	14.773	19.337			
M16	14.691	18.547			
M17	13.911	18.444			
M18	14.201	17.071			
M19	14.373	20.572			
M20	14.740	19.902			
M21	15.718	21.910			
M22	15.064	22.975			

Figure 1E

	GAPDH	METTL5
HS-5	16.209	19.832
	16.226	20.054
	16.350	20.129
MM1S	16.113	19.270
	16.292	19.262
	16.308	19.412
H929	16.182	19.672
	16.371	19.363
	16.358	19.423
U266	16.275	18.321
	16.426	18.346
	16.443	18.522
RPMI-8226	16.166	19.032
	16.206	18.924
	16.192	18.841

Figure 2A

	GAPDH	METTL5
LV	13.613	17.725
	13.787	17.809
	13.893	17.643
LV-METTL5	13.985	17.044
	13.968	17.073
	14.163	17.187

Figure 3A

	GAPDH	METTL5
NC	14.377	18.196
	14.023	18.155
	13.763	18.113
SH1	13.627	21.367
	13.700	21.100
	13.269	21.186
SH2	13.604	21.263
	13.768	20.953
	13.578	20.842

Figure 4F

	GAPDH	METTL5
NC	14.841	17.530
	14.649	17.823
	14.586	17.916
SH1	14.563	19.011
	14.496	19.059
	14.435	18.742
SH2	14.303	19.150
	14.349	19.185
	14.382	19.095

	GAPDH	SPEHS2
NC	14.841	20.179
	14.649	20.244
	14.586	20.142
SH1	14.563	20.186
	14.496	20.230
	14.435	20.239
SH2	14.303	20.281
	14.349	20.285
	14.382	20.307

Figure 4G

	GAPDH	METTL5
NC	14.186	17.661
	13.968	17.566
	14.291	17.609
SH1	14.263	19.984
	14.236	20.026
	14.269	19.940
SH2	14.390	19.983
	14.386	20.013
	14.393	19.985

	GAPDH	SPEHS2
NC	14.186	20.221
	13.968	20.154
	14.291	20.120
SH1	14.263	20.237
	14.236	20.199
	14.269	20.237
SH2	14.390	20.173
	14.386	20.169
	14.393	20.174

Figure 4J and K

NC

	<40	40	60	80	P
SEPHS2	22.929	20.212	23.761	25.108	21.172
	23.224	20.411	23.322	25.110	20.996
	22.587	20.072	23.181	24.902	21.162
GAPDH	16.934	14.890	18.606	19.904	15.202
	16.360	14.840	18.149	19.706	15.217
	16.860	14.742	18.493	20.019	15.205

SH1

<40	40	60	80	P
25.708	24.870	25.003	25.865	25.529
25.887	24.710	24.949	25.294	25.606
25.766	24.859	24.772	25.146	25.623
16.517	14.025	18.109	20.553	14.132
16.404	14.124	18.902	20.013	14.602
16.656	14.071	18.156	20.387	14.615

Figure S2A

	GAPDH	METTL5
LV	16.972	19.610
	16.700	19.653
	16.672	19.387
LV-METTL5	17.803	18.926
	17.735	18.730
	17.699	18.745

Figure S3A

	GAPDH	METTL5
NC	12.878	16.593
	13.308	16.659
	12.339	16.577
SH1	12.790	18.263
	12.688	18.283
	12.612	18.496
SH2	12.682	18.995
	13.429	18.746
	12.645	19.219

Figure S5E and F

NC

	<40	40	60	80	P
SEPHS2	25.144	23.060	26.287	27.425	23.160
	25.617	23.601	26.156	27.482	23.140
	25.405	23.104	26.236	27.336	23.123
GAPDH	18.075	14.823	19.925	21.915	15.499
	18.241	15.259	19.595	21.848	15.626
	18.206	15.325	19.673	21.977	15.697

SH1

	<40	40	60	80	P
	26.095	24.904	25.250	25.716	24.703
	26.319	24.803	25.245	25.431	24.576
	26.242	24.861	25.330	25.336	24.313
	17.996	14.918	20.106	22.846	15.297
	18.165	14.912	20.274	22.858	15.804
	18.197	14.919	20.587	23.006	15.367

Figure S5A

	GAPDH	METTL5
LV	16.284	19.440
	16.373	19.441
	16.336	19.364
LV-METTL5	15.994	17.214
	16.072	17.194
	16.028	17.353

	GAPDH	SEPHS2
LV	16.284	21.069
	16.373	21.075
	16.336	21.080
LV-METTL5	15.994	20.630
	16.072	20.799
	16.028	20.630

Figure S5B

	GAPDH	METTL5
H929-VEC	14.177	18.589
	14.140	18.556
	14.183	18.568
H929-OE	14.005	17.326
	14.038	17.238
	14.026	17.228

	GAPDH	SEPHS2
H929-VEC	14.177	19.595
	14.140	19.751
	14.183	19.663
H929-OE	14.005	19.468
	14.038	19.498
	14.026	19.631

Figure S6A

	GAPDH	METTL5
Vector+NC	15.621	18.861
	15.526	18.855
	15.346	18.791
Vector+shSEPHS2	15.051	17.464
	15.148	17.569
	15.009	17.368
METTL5OE+NC	16.043	19.685
	16.065	19.567
	16.120	19.669
METTL5+shSEPHS2	15.609	18.118
	15.578	17.889
	15.385	17.933

	GAPDH	SPEHS2
Vector+NC	15.621	20.590
	15.526	20.606
	15.346	20.589
Vector+shSEPHS2	15.051	22.476
	15.148	22.467
	15.009	22.368
METTL5OE+NC	16.043	20.970
	16.065	21.112
	16.120	21.130
METTL5+shSEPHS2	15.609	22.995
	15.578	22.990
	15.385	22.980

Figure S6B

	GAPDH	METTL5
H929-Vec+NC	14.052	18.386
	13.964	18.275
	13.998	18.293
H929-Vec+shSEPHS2	13.935	18.283
	13.964	18.273
	13.863	18.290
H929-METTL5+NC	13.841	16.967
	13.946	16.967
	13.637	16.951
H929-METTL5+shSEPHS2	13.681	16.608
	13.366	16.669
	13.368	16.651

	GAPDH	SEPHS2
H929-Vec+NC	14.052	17.982
	13.964	17.953
	13.998	17.989
H929-Vec+shSEPHS2	13.841	19.565
	13.946	19.637
	13.637	19.550
H929-METTL5+NC	13.681	17.713
	13.366	17.553
	13.368	17.590
H929-METTL5+shSEPHS2	13.841	19.565
	13.946	19.496
	13.637	19.655

Figure S7A

	GAPDH	METTL5
NC+Vector	14.172	18.793
	14.078	18.737
	14.071	18.610
NC+SEPHS2OE	14.237	18.749
	14.199	18.711
	14.242	18.792
shMETTL5-1+Vector	14.198	20.409
	14.032	20.569
	14.162	20.248
shMETTL5-1+SEPHS2OE	14.389	20.690
	14.165	20.401
	14.121	20.335
shMETTL5-2+Vector	14.148	20.870
	14.128	20.656
	14.331	20.460
shMETTL5-2+SEPHS2OE	14.395	20.634
	14.440	20.517
	14.356	20.329

	GAPDH	SPEHS2
NC+Vector	14.172	20.013
	14.078	20.042
	14.071	20.044
NC+SEPHS2OE	14.237	18.801
	14.199	18.778
	14.242	18.804
shMETTL5-1+Vector	14.198	20.174
	14.032	20.182
	14.162	20.292
shMETTL5-1+SEPHS2OE	14.389	18.855
	14.165	18.808
	14.121	19.032
shMETTL5-2+Vector	14.148	20.035
	14.128	20.169
	14.331	20.101
shMETTL5-2+SEPHS2OE	14.395	18.995
	14.440	18.976
	14.356	19.015

Figure S7B

	GAPDH	METTL5
NC+Vector	14.204	17.518
	14.135	17.349
	14.235	17.232
NC+SEPHS2OE	14.263	17.384
	14.506	17.200
	14.223	17.552
shMETTL5-1+Vector	14.036	19.638
	13.964	19.816
	13.836	19.667
shMETTL5-1+SEPHS2OE	14.058	19.487
	13.881	19.237
	13.636	19.102
shMETTL5-2+Vector	14.212	19.402
	14.240	19.382
	14.262	19.192
shMETTL5-2+SEPHS2OE	14.041	18.789
	14.040	18.791
	14.319	18.886

	GAPDH	SPEHS2
NC+Vector	14.204	18.168
	14.135	18.217
	14.235	18.326
NC+SEPHS2OE	14.263	17.357
	14.506	17.470
	14.223	17.477
shMETTL5-1+Vector	14.036	18.093
	13.964	17.962
	13.836	18.006
shMETTL5-1+SEPHS2OE	14.058	16.869
	13.881	16.821
	13.636	16.887
shMETTL5-2+Vector	14.212	18.550
	14.240	18.200
	14.262	18.417
shMETTL5-2+SEPHS2OE	14.041	16.923
	14.040	16.871
	14.319	17.293
